# Prevalence and correlates of common mental health problems and recent suicidal thoughts and behaviours among female sex workers in Nairobi, Kenya

**DOI:** 10.1186/s12888-021-03515-5

**Published:** 2021-10-14

**Authors:** Alicja Beksinska, Zaina Jama, Rhoda Kabuti, Mary Kungu, Hellen Babu, Emily Nyariki, Pooja Shah, Maisha Fiti Study Champions, Demtilla Gwala, Daisy Oside, Ruth Kamene, Agnes Watata, Agnes Atieno, Faith Njau, Elizabeth Njeri, Evelyn Orobi, Ibrahim Lwingi, Chrispo Nyabuto, Monica Okumu, Anne Mahero, Pauline Ngurukiri, Erastus Irungu, Wendy Adhiambo, Peter Muthoga, Rupert Kaul, Janet Seeley, Tara S. Beattie, Helen A. Weiss, Joshua Kimani

**Affiliations:** 1 Department of Global Health and Development, London School of Hygiene and Tropical Medicine, London, UK; 2 UK Partners for Health and Development in Africa (PHDA), UNITID, College of Health Sciences, Nairobi, Kenya; 3 University of Toronto, Toronto, Canada; 4 MRC International Statistics & Epidemiology Group, Department of Infectious Disease Epidemiology, London School of Hygiene and Tropical Medicine, London, UK

**Keywords:** Mental health, Female sex workers, Depression, Anxiety, Post-traumatic stress disorder, Suicide, Kenya

## Abstract

**Background:**

Adverse childhood experiences (ACEs), poverty, violence and harmful alcohol/substance use are associated with poor mental health outcomes, but few studies have examined these risks among Female Sex Workers (FSWs). We examine the prevalence and correlates of common mental health problems including suicidal thoughts and behaviours among FSWs in Kenya.

**Methods:**

Maisha Fiti is a longitudinal study among FSWs randomly selected from Sex Worker Outreach Programme (SWOP) clinics across Nairobi. Baseline behavioural-biological survey (*n* = 1003) data were collected June–December 2019. Mental health problems were assessed using the Patient Health Questionnaire (PHQ-9) for depression, the Generalised Anxiety Disorder tool (GAD-7) for anxiety, the Harvard Trauma Questionnaire (HTQ-17) for Post-Traumatic Stress Disorder (PTSD) and a two-item tool to measure recent suicidal thoughts/behaviours. Other measurement tools included the WHO Adverse Childhood Experiences (ACE) score, WHO Violence Against Women questionnaire, and the Alcohol, Smoking and Substance Involvement Screening Test (ASSIST). Descriptive statistics and multivariable logistic regression were conducted using a hierarchical modelling approach.

**Results:**

Of 1039 eligible FSWs, 1003 FSWs participated in the study (response rate: 96%) with mean age 33.7 years. The prevalence of moderate/severe depression was 23.2%, moderate/severe anxiety 11.0%, PTSD 14.0% and recent suicidal thoughts/behaviours 10.2% (2.6% suicide attempt, 10.0% suicidal thoughts). Depression, anxiety, PTSD and recent suicidal thoughts/behaviours were all independently associated with higher ACE scores, recent hunger (missed a meal in last week due to financial difficulties), recent sexual/physical violence and increased harmful alcohol/substance. PTSD was additionally associated with increased chlamydia prevalence and recent suicidal thoughts/behaviours with low education and low socio-economic status. Mental health problems were less prevalent among women reporting social support.

**Conclusions:**

The high burden of mental health problems indicates a need for accessible services tailored for FSWs alongside structural interventions addressing poverty, harmful alcohol/substance use and violence. Given the high rates of ACEs, early childhood and family interventions should be considered to prevent poor mental health outcomes.

**Supplementary Information:**

The online version contains supplementary material available at 10.1186/s12888-021-03515-5.

## Introduction

There is a high burden of untreated common mental health problems in low−/low-middle income countries (LMIC) resulting in short- and long-term impacts on quality of life, and increased risk of mortality from suicide as well as other physical health conditions [1]. According to the 2019 Global Burden of Disease study, among women in LMICs the prevalence of depression is 4.5% (95%CI: 4.0–5.0) (men: 3.0%; 95%CI: 2.7–3.4) and the prevalence of anxiety disorders is 4.3% (95%CI: 3.6–5.2%) (men: 2.8%; 95%CI: 2.3–3.3%) [2]. Depressive disorders are the third leading cause of years lost to disability globally [2, 3]. The prevalence of Post-Traumatic Stress Disorder (PTSD) symptoms in sub-Saharan Africa is estimated at 22.0% (95% CI 13.0–32.0%) [4], though estimates vary due to heterogeneity in measurement tools.

Sex work (defined as the receipt of money or goods in exchange for sexual services) is criminalised in most parts of the world [5, 6]. Female sex workers (FSWs) face intersecting socio-economic and structural inequalities starting in early childhood that may increase their risk of mental health problems across their life course. These inequalities include poverty, low education, childhood neglect and violence, gender inequality, police arrest, alcohol and substance use, discrimination, violence from clients and intimate partners and high prevalence of Human Immunodeficiency Virus (HIV)/Sexually Transmitted Infections (STIs) [6, 7]. Results from a recent systematic review and meta-analysis examining mental health among FSWs in LMICs show much higher levels of mental health problems among FSWs compared to the general population [8]. The pooled prevalence for depression is 41.8% (95% CI 35.8–48.0%), anxiety 21.0% (95% CI: 4.8–58.4%), PTSD 19.7% (95% CI: 3.2–64.6%), suicidal thoughts 22.8% (95% CI 13.2–36.5%) and recent suicide attempt 6.3% (95% CI 3.4–11.4%) [8]. However, the reliability of these estimates is limited by the use of varied measurement tools and inconsistent cut-off scores and time-frames.

The pathways established for risk associations with mental health problems in the general population are likely to be exacerbated among FSWs. A systematic review among FSWs found associations between depression and risk factors including violence experience (pooled OR: 2.3; 95%CI: 1.3–4.2), alcohol use (2.1; 95%CI: 1.4–3.2), inconsistent condom use with clients (1.6; 95%CI: 1.2–2.1) and HIV infection (1.4; 95%CI: 1.1–1.8) [8]. In addition, they reported associations of recent suicide attempt with violence ever (pooled unadjusted OR: 3.5; 95%CI: 2.2–5.5), recent suicidal thoughts and alcohol use (1.6; 1.0–2.5) and recent suicidal thoughts and HIV (1.4; 95%CI: 1.1–1.8).

Most studies examining mental health in FSWs have focussed on depression, with few exploring risk factors for anxiety, PTSD or suicide risk. Alcohol and substance use are common among FSWs [9] and are associated with mental health problems in the general population [10], but this is less well studied among FSWs. A small number of studies have reported on high levels of childhood physical and sexual violence among FSWs [11–13]. Adverse Childhood Experiences (ACEs) have a strong impact on adult mental health outcomes [14] in the general population, but again this has not been explored among FSWs. Most research among FSWs has focussed on addressing HIV/STI risk through behavioural and biomedical interventions [6], although more recently some interventions have targeted key structural drivers such as violence [15, 16]. However, there are currently no studies assessing mental health interventions among FSWs. Increased understanding of the prevalence and risk factors for specific mental health problems are needed to inform future interventions among this population.

In Nairobi County, Kenya, 39,000 women are estimated to sell sex [17]. The Maisha Fiti study aimed to examine associations between key structural and social risk factors, such as violence experience and mental health problems, and biological changes to the immune system which may increase HIV susceptibility. Baseline data was analysed to examine prevalence and risk factors for depression, anxiety, PTSD and suicidal behaviours using validated measurement tools. We hypothesise that structural and behavioural risk factors across the life course including childhood experiences, sexual risk behaviours and harmful alcohol/substance use will be associated with increased prevalence of mental health problems. We hope that these findings will be used to better understand the burden of mental health problems among FSWs and potential areas for targeted treatment and prevention.

## Methods

### Study design and sampling

We used baseline cross-sectional data collected from June–December 2019 in the Maisha Fiti study. The study was designed in consultation with FSWs in Nairobi and with community mobilisers and staff working at seven Sex Worker Outreach Programme (SWOP) clinics.

All women attending SWOP clinics have a unique clinic barcode. Barcodes were randomly selected from the SWOP clinic attendance lists of all attendees in the previous 12 months. Those randomly selected were contacted and invited to enrol in the study. There were 10, 292 women in the sampling frame, from whom potential participants were randomly sampled proportional to clinic size from the seven SWOP clinics. Assuming 2:1 exposure of recent sexual or physical violence, enrolling 750 HIV-negative women was calculated to detect a 10% difference in the proportion of women who have genital inflammation (25% vs. 15%) at 90% power. HIV prevalence among FSWs attending SWOP clinics in Nairobi is approximately 25%, therefore the target sample size was 1000 FSWs. In total 1200 FSWs were randomly sampled, of whom 1039 were eligible.

Eligibility criteria included women aged 18–45 years, who had attended one of the SWOP clinics in the past 12 months, were not pregnant or breast-feeding, and did not have an underlying chronic illness (other than HIV) that was likely to alter host immunology. Selected women were telephoned, told about the study and invited to attend the study clinic. Interested women were given an appointment at the study clinic where they were screened for eligibility and received detailed information about the study verbally and via the participant information leaflet. For women with low literacy, this information was read to them by study staff. Consenting participants completed a behavioural-biological survey. Those found to have mental health or alcohol/substance use problems were referred to a trained psychological counsellor based in the study clinic for assessment and treatment. All women who tested positive for HIV during the study were counselled and encouraged to enrol in HIV care. All women who tested positive for STIs were offered appropriate treatment free of charge.

### Behavioural-biological survey

Our main outcomes of interest were mental health problems and recent suicide risk. Women completed a baseline questionnaire about socio-demographics, sex work characteristics, mental health, ACEs, violence, alcohol/substance use and sexual risk behaviours (Supplementary file 1). Validated tools with high reliability and validity were used for measuring mental health problems included the Patient Health Questionnaire (PHQ-9) for depression (score ≥ 15 = moderate/ severe depressive disorder) (Cronbach’s α =0.89, sensitivity = 88%, specificity =88%) [18]; Generalised Anxiety Disorder (GAD-7) tool for anxiety (score ≥ 10 = moderate/severe anxiety) (Cronbachs α =0.92, sensitivity =89%, specificity = 82% [19] and the Harvard Trauma Questionnaire (HTQ-17) for PTSD (score ≥ 2.5 positive for PTSD) (Cronbachs α = 0.8–0.9) [20]. The PHQ-9, GAD-7, and HTQ-17 are all based on criteria from the Diagnostic and Statistical Manual of Mental Disorders (DSM-IV) [21].

Suicide risk was measured by a two-item questionnaire which included recent suicidal thoughts (‘*having thoughts about ending your life in the last 30 days*’) and recent suicide attempt (‘*having attempted to end your life in the last 30 days’).* Due to the small number of women reporting a recent suicide attempt, and the significant overlap in women reporting a recent suicide attempt and recent suicidal thoughts we combined these measures into a dichotomous variable including women who reported recent suicidal thoughts *and/or* recent suicide attempt under the term ‘recent suicidal thoughts/behaviours’. Previous suicidal thoughts *and/or* suicide attempts have been shown in meta-analysis to significantly increase the lifetime risk of further suicide attempts and completed suicide [22, 23] and about 60% of the transitions from suicidal thoughts to planning to suicide attempt, take place in the first year after first episode of suicidal thoughts [4]. The term ‘suicidal thoughts/behaviours’ encompasses a continuum of suicide risk from suicidal thoughts to planning to attempts and is a recognised definition in the psychiatric literature [24–27].

The WHO ASSIST (Alcohol, Smoking and Substance Involvement Screening Test) tool was used to measure harmful alcohol (cut-off scores: moderate risk > 11; high risk > 27) and other substance use (cut-off scores: moderate risk > 4; high risk > 27) in the last 3 months including amphetamines, cannabis, cocaine, hallucinogens, sedatives and inhalants [28].

ACEs were measured using the WHO Adverse Childhood Experiences International Questionnaire (ACE-IQ) [29]. Due to the length of the questionnaire, three questions from the WHO ACE-IQ tool were not included (one question on bullying from peers and two questions on emotional and physical neglect from parents/guardians were excluded). An additional question about street homelessness in childhood was incorporated as it was considered relevant for this population. We examined individual associations of specific ACEs with the outcomes and also created a three-item categorical ACE score variable which comprised 12 components (*1- household member depressed/institutionalised/suicidal; 2- household member misused alcohol/substances; 3- household member in prison; 4- parents divorced/separated; 5- parent/guardian died; 6 – witnessed emotional/physical violence between household members; 7-lived on the street in childhood; 8-experienced emotional violence; 9- experienced sexual violence; 10- experienced physical violence; 11- witnessed violence in the community; 12- experienced displacement/destruction of home/violence during war*). We generated three categories of increasing number of ACEs (≤4; 5–8; 9–12) with each ACE scoring one point. This approach was guided by a systematic review and meta-analysis that reports ACEs have a cumulative impact on subsequent mental health problems [14] and on the WHO ACE-IQ scoring tool [29].

The WHO Violence Against Women 13-item questionnaire which measures frequency and severity of Intimate Partner Violence (IPV) was adapted to include violence perpetrated by non-IPs (e.g. police, strangers, clients etc) [30]. We asked about violence experiences ever and in the past 6 months.

Social support was defined as women reporting yes to the following question ‘*Do you have someone who you can talk to about your problems?*’

### Laboratory methods

Urine samples were collected to test for pregnancy as well as *Neisseria gonorrhoeae* (NG) and *Chlamydia trachomatis* (CT) (using Gene Expert Assay). Blood was collected for *Treponema pallidum* (syphilis) diagnostics using the rapid plasma reagin assay. HIV status was screened by rapid HIV tests, with positive tests confirmed using HIV DNA Genexpert. Self-collected vaginal swabs were used to diagnose Trichomonas vaginalis (TV; OSOM Trichomonas Rapid Test; SEKISUI Diagnostics, LLC) and Bacterial Vaginosis (BV; Gram’s stain and Nugent scoring).

### Conceptual framework

**Fig. 1:**
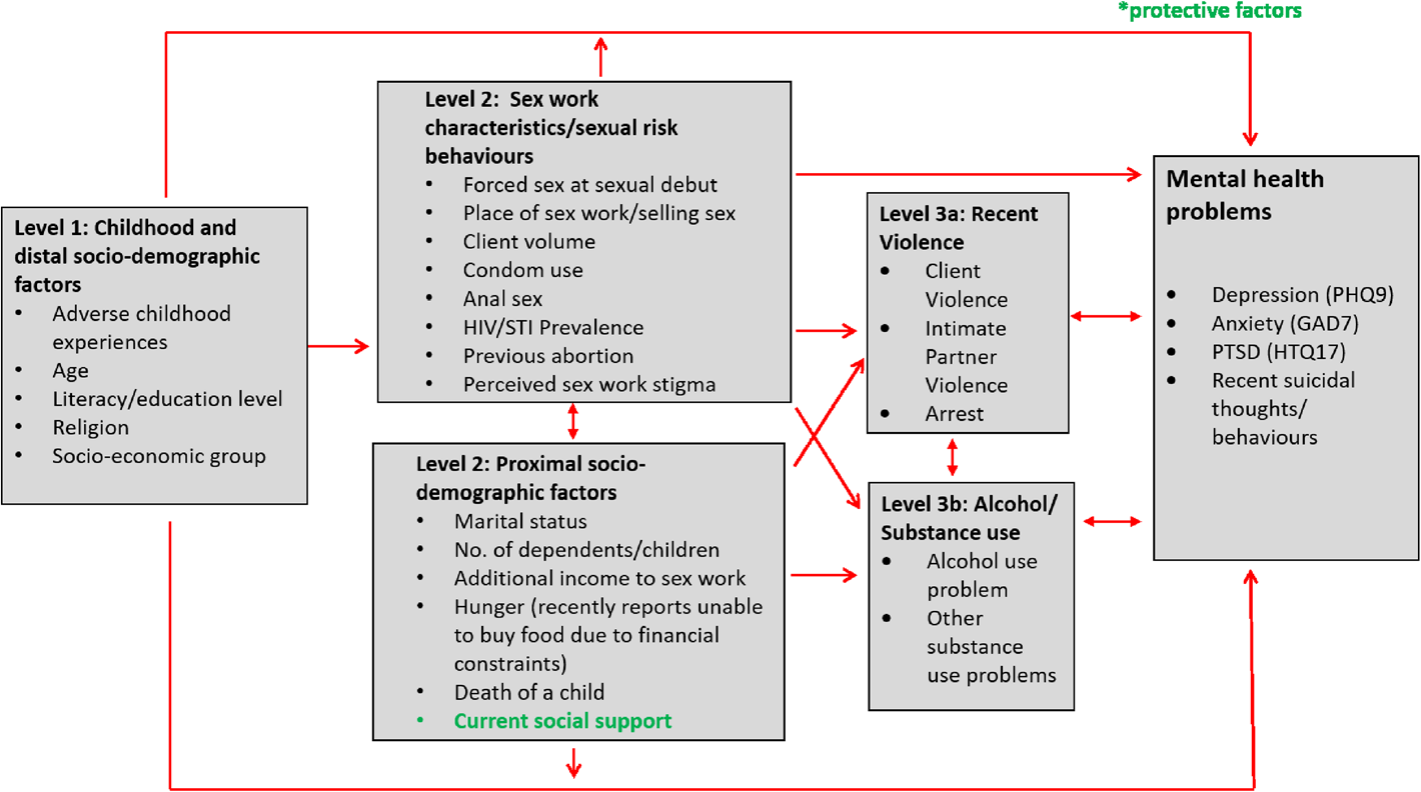
Conceptual framework for exploring risk factors for mental health problems across the life course

We developed a conceptual framework (Fig. 1) to explore correlates of mental health problems and recent suicidal behaviour using an eco-social life course theory [31]. Depression and anxiety were combined for analysis due to the overlap between reporting of these conditions and their common treatment pathway. Level 1 variables included ACEs and distal socio-demographic factors such as literacy level and Socio-economic Status (SES). Level 2 variables include proximal socio-demographic factors such as marital status and social support as well as sex work characteristics and HIV/STI risk behaviours. Level 3a and 3b variables included alcohol and substance use problems and recent violence experience.

### Statistical analyses

Data were double-entered and statistical analyses were conducted in STATA 16.1 (Stata Inc., College Station, TX, USA). As women < 25 years old were over-sampled data were weighted for age during analysis. To adjust for clustering by clinic, SWOP clinic was included as a fixed effect in all multivariable models. Socio-demographic and behavioural variables associated with a mental health problem or recent suicidal thoughts/behaviours (*p*-value < 0.1) in univariate analyses were included in multivariable logistic regression, as well as a core group of a priori level 1 socio-demographics including age, socioeconomic status and education status (literacy). Due to the large overlap in women experiencing either mental health problem and their common treatment pathway depression and anxiety were combined into moderate/severe depression *and/or* moderate/severe anxiety for analysis. Associations were estimated using odds ratios (OR), with *p*-values obtained using a joint hypothesis test via the adjusted Wald test (to allow for sampling weights). We used a hierarchical modelling approach [32] to build multivariable models for each outcome based on our conceptual framework, which describes the hierarchical relationship between distal and proximal determinants of mental health problems and suicidal thoughts/behaviours. Hierarchical modelling involves incorporating temporal, biological and social understandings about the relationship between determinants of disease and takes into account the effect of mediating variables. The overall effect of level 1 variables were examined in model 1 adjusted for other level 1 variables but not level 2 or 3 variables as these represent mediating factors. In model 2, level 2 variables were examined adjusted for level 1 and 2 variables and model 3a and 3b adjusted for level 1, 2 and either 3a or 3b variables.

We examined stratum specific estimates and used the adjusted Wald test to test for potential effect modification for specific variables (alcohol/substance use and recent violence experience). Missing data was reported if > 5% of observations were missing.

## Results

### Sample demographics and sex work characteristics

Of the 1039 eligible FSWs, 1003 FSWs took part in the study (baseline response rate: 96%). The median age was 32 years (range 18–45 years) with 11.7% aged < 25 years and 48.9% aged > 35 years. Most women had previously been married (81.2%), but over two thirds (69.2%) were currently living with children and no partner, with just 6.8% living with a male partner. Less than half (43.7%) of women reported an additional income to sex work and one third (33.9%) reported missing a meal in the last week due to financial constraints. The mean age at first sex was 16.3 years with 5.4% of women reporting sexual debut < 13 years. Most women (91.4%) worked from a lodge/hotel/rented room and reported a median client volume per week of 3 (range 0–70). HIV prevalence was 28.0%, with no new HIV diagnoses during the survey, and chlamydia was the most prevalent STI (5.7%) *(*Table 1*).*

**Table 1:** Study sample characteristics and associations with mental health problems and recent suicidal thoughts/behaviours

			N(%) (*N* = 1003)	% with depression and/or anxiety (*N* = 243)	% with PTSD (*N* = 138)	% with reported recent suicidal thoughts/behaviours (*N* = 101)
*Level 1: Adverse Childhood Experiences and distal socio-demographic characteristics*						
**Age (years)**	< 25		212 (11.7%)	8.4	9.4	10.3
	25–34		353 (39.4%)	37.5	35.8	41.6
	35+		438 (48.9%)	54.1	54.8	48.1
**Adverse Childhood Experience**	Lived on the street	No	878 (88.0%)	83.3	82.2	73.8
		Yes	125 (12.0%)	16.7	17.8	26.2
	Adverse family/household experience^a^	No	113 (11.3%)	5.6	4.5	3.9
		Yes	883 (88.7%)	94.4	95.5	96.1
	Experienced emotional abuse	No	324 (32.1%)	19.5	18.3	14.2
		Yes	678 (67.9%)	80.5	81.7	85.8
	sexual and/or physical violence	No	212 (20.7%)	9.6	8.7	7.7
		Yes	791 (79.3%)	90.4	91.3	92.4
	War/community violence	No	95 (10.1%)	4.7	4.8	8.7
		Yes	907 (89.9%)	95.3	95.2	91.3
	Total number of ACEs reported	< 4	281 (28.7%)	13.9	11.4	15.0
		5–8	540 (54.1%)	51.9	55.5	41.7
		9–12	170 (17.2%)	34.2	33.1	43.3
**Literacy**	Illiterate		166 (17.6%)	19.7	17.9	27.4
	Literate		837 (82.4%)	80.4	82.1	72.7
**Religion**	Catholic		375 (36.9%)	33.6	31.8	36.1
	Protestant		534 (54.4%)	55.0	60.3	48.6
	Muslim		46 (4.6%)	6.3	5.2	8.7
	Other/None		46 (4.6%)	5.1	2.8	6.5
**Socio-economic status (SES)**	Low/low-middle		401 (39.3%)	42.4	36.1	56.4
	Middle		200 (19.9%)	21.2	25.4	19.9
	Upper middle/upper		400 (40.8%)	36.4	38.5	23.8
*Level 2: Proximal socio-demographic characteristics, sex work characteristics, sexual risk behaviours, HIV and STI prevalence*						
**Marital status**	Ever married	No	216 (18.8%)	15.4	15.1	16.4
		Yes	787 (81.2%)	84.6	84.9	83.6
**No. of children**^b^	None		40 (3.4%)	3.6	5.7	6.7
	1–2		644 (66.1%)	61.5	62.2	50.5
	3+		264 (30.5%)	34.9	32.1	42.8
**Reports death of a child**^b^		No	829 (86.8%)	81.6	83.7	81.1
		Yes	119 (13.2%)	18.4	16.3	18.9
**Income**	Additional income to sex work	No	571 (56.4%)	49.1	46.0	48.1
		Yes	432 (43.7%)	50.9	54.0	51.9
**Hunger**	Missed a meal in the last 7 days due to financial constraints	No	670 (66.1%)	51.6	53.2	43.4
		Yes	331 (33.9%)	48.5	46.8	56.6
**Social support**	Someone to talk to about your problems	No	278 (27.5%)	34.2	37.7	37.7
		Yes	725 (72.5%)	65.9	62.3	62.3
**Non-consensual sexual debut**	Tricked, pressured or physical forced into first sex	No	695 (68.7%)	51.8	44.0	51.7
		Yes	306 (31.3%)	48.2	56.0	48.3
**Age first sex work**	Mean (years)		24.4	25.2	24.5	23.8
**Place of selling sex**	Phone/internet/friends		54 (5.4%)	6.5	7.6	6.1
	Home/middle men/markets		15 (1.6%)	1.8	3.2	0
	Brothel/escort service/massage parlour		14 (1.5%)	1.8	0.8	0
	Bar/club/lodge/social gatherings		620 (61.5%)	55.4	60.0	64.1
	Street/bus/taxis		294 (30.0%)	34.5	28.4	29.8
**Place of sex work**	Lodge/hotel/rented room		907 (91.4%)	90.3	85.6	86.2
	Other public place		28 (2.8%)	4.1	2.8	6.1
	Home		60 (5.8%)	5.6	11.6	7.7
**Client volume/week**	Median		3	3	3	4
	< 5		607 (60.9%)	64.7	62.9	59.2
	5–9		350 (24.8%)	20.6	21.0	27.9
	10+		137 (14.2%)	14.7	16.1	12.9
**Condom use last sex**		No	236 (22.8%)	27.2	27.4	22.1
		Yes	765 (77.2%)	72.8	72.6	77.8
**Anal sex with client**	Last 7 days	No	980 (98.4%)	96.9	92.4	95.0
		Yes	16 (1.6%)	3.2	7.6	5.0
**Sex work stigma**	Any perceived stigma related to sex work	No	140 (14.0%)	4.8	8.4	6.2
		Yes	855 (86.0%)	95.3	91.6	93.9
**Abortion/stillbirth**^c^	Pregnancy that ended in stillbirth or abortion	No	539 (55.4%)	46.3	39.8	47.8
		Yes	412 (44.6%)	53.8	60.2	42.3
**HIV status**		Negative	746 (72.0%)	72.6	72.2	72.1
		Positive	257 (28.0%)	27.3	27.8	27.9
***Chlamydia Trachomatis***		Negative	932 (94.3%)	94.9	91.6	93.5
		Positive	67 (5.7%)	5.1	8.4	6.5
**Neisseria Gonorrhoea**		Negative	975 (97.4%)	97.8	98.8	98.9
		Positive	67 (2.6%)	2.2	1.2	1.1
**Syphilis (*Treponema*** ***pallidum*)**		Negative	979 (97.9%)	97.5	97.2	96.7
		Positive	20 (2.1%)	2.5	2.8	3.3
**Bacterial vaginosis**		Negative	428 (42.1%)	46.6	48.4	40.4
		Positive	199 (20.0%)	20.3	17.9	16.9
		Intermediate	371 (37.9%)	33.1	33.7	42.7
**Trichomonas vaginalis**		Negative	969 (97.0%)	97.5	98.0	97.2
		Positive	31 (3.0%)	2.5	2.0	2.8
*Level 3a: Recent violence/arrest (last 6 months)*						
**Any recent violence (physical/sexual/emotional)**		No	198 (19.3%)	11.6	6.4	6.6
		Yes	805 (80.7%)	88.4	93.7	93.4
**Any recent sexual and/or physical non-IP violence**		No	456 (45.0%)	30.8	27.8	23.5
		Yes	547 (55.0%)	69.2	72.2	76.5
**Any recent sexual and/or physical IP violence**		No	693 (69.1%)	63.2	53.6	55.8
		Yes	310 (30.9%)	36.8	46.4	44.3
**Recent arrest**		No	701 (69.3%)	64.1	61.1	52.5
		Yes	302 (30.7%)	35.9	38.9	47.6
*Level 3b: Harmful alcohol and/or substance use last 3 months*						
**Alcohol use problem**	ASSIST risk level	Low/none	697 (70.1%)	47.3	40.1	48.4
		Moderate/high	302 (29.9%)	52.7	59.9	51.6
**Other substance use problem**^d^	ASSIST risk level	Low/none	681 (69.3%)	53.8	56.0	49.7
		Moderate/high	322 (30.7%)	46.2	44.0	50.3
		Moderate/high	455 (44.2%)	64.9	68.7	67.8

^a^at least one of: household member or guardian depressed/suicidal, household member/guardian arrested, household member/guardian had an alcohol use problem, parental death, parental divorce/separation

^b^ missing *n* = 55.

^c^missing *n* = 52.

^d^moderate/high risk cannabis/amphetamine/cocaine/sedative/inhalants/hallucinogen use.

### Mental health problems and recent suicidal thoughts/behaviours

**Fig. 2:**
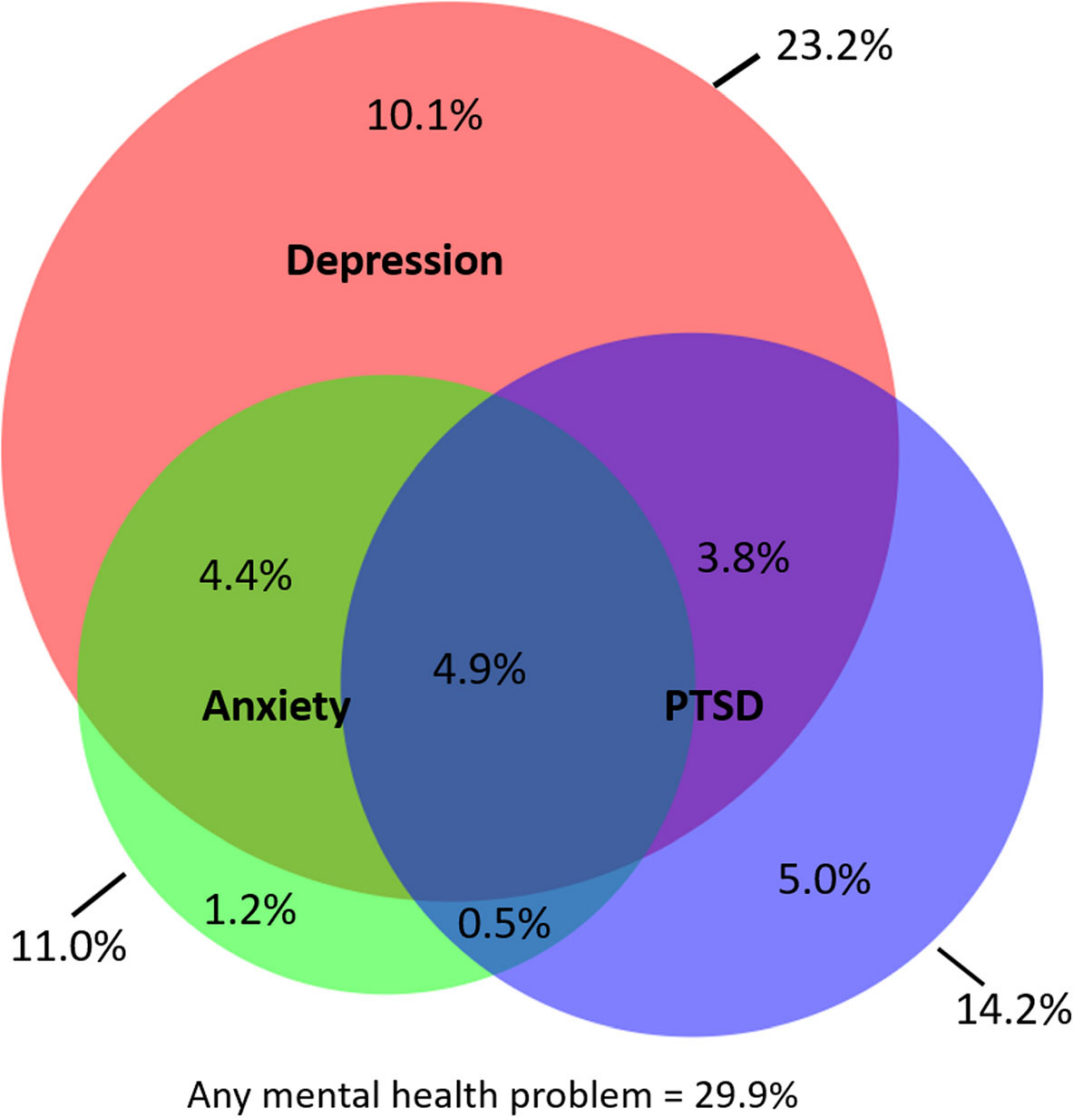
Overlap between common mental health problems [33]

Almost half of women (49.3%) reported any symptoms of depression (PHQ9 score ≥ 5) and 38.4% had any symptoms of anxiety (GAD-7 score ≥ 5), with 23.2% (95%CI: 20.7–25.9%) reporting moderate/severe depression and 11.0% (95%CI: 9.3–13.1%) reporting moderate/severe anxiety. One quarter (25.0%; 95%CI: 22.5–27.8%) of women had either moderate/severe depression or moderate/severe anxiety. The prevalence of PTSD was 14.2% (95% CI: 12.2–16.5%). Overall 4.6% of women reported the co-occurrence of depression, anxiety *and* PTSD (Fig. 2*).* Internal consistency was high for our main outcome measures (Cronbach’s α = 0.90 for PHQ-9; α = 0.94 for GAD-7; α = 0.87 for HTQ-17).

**Fig. 3:**
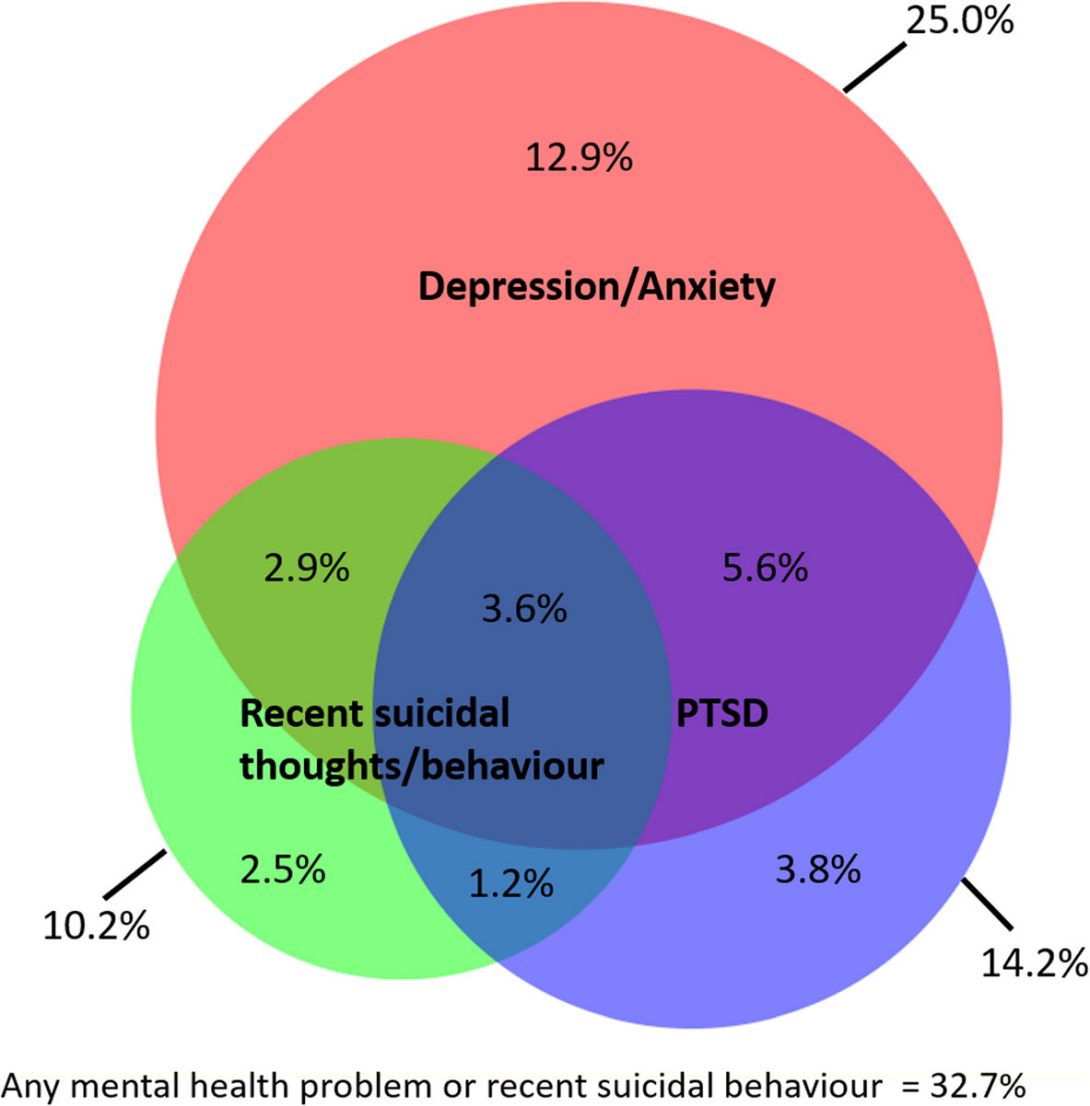
Overlap between mental health problems and recent suicidal thoughts/behaviours

**Fig. 4:**
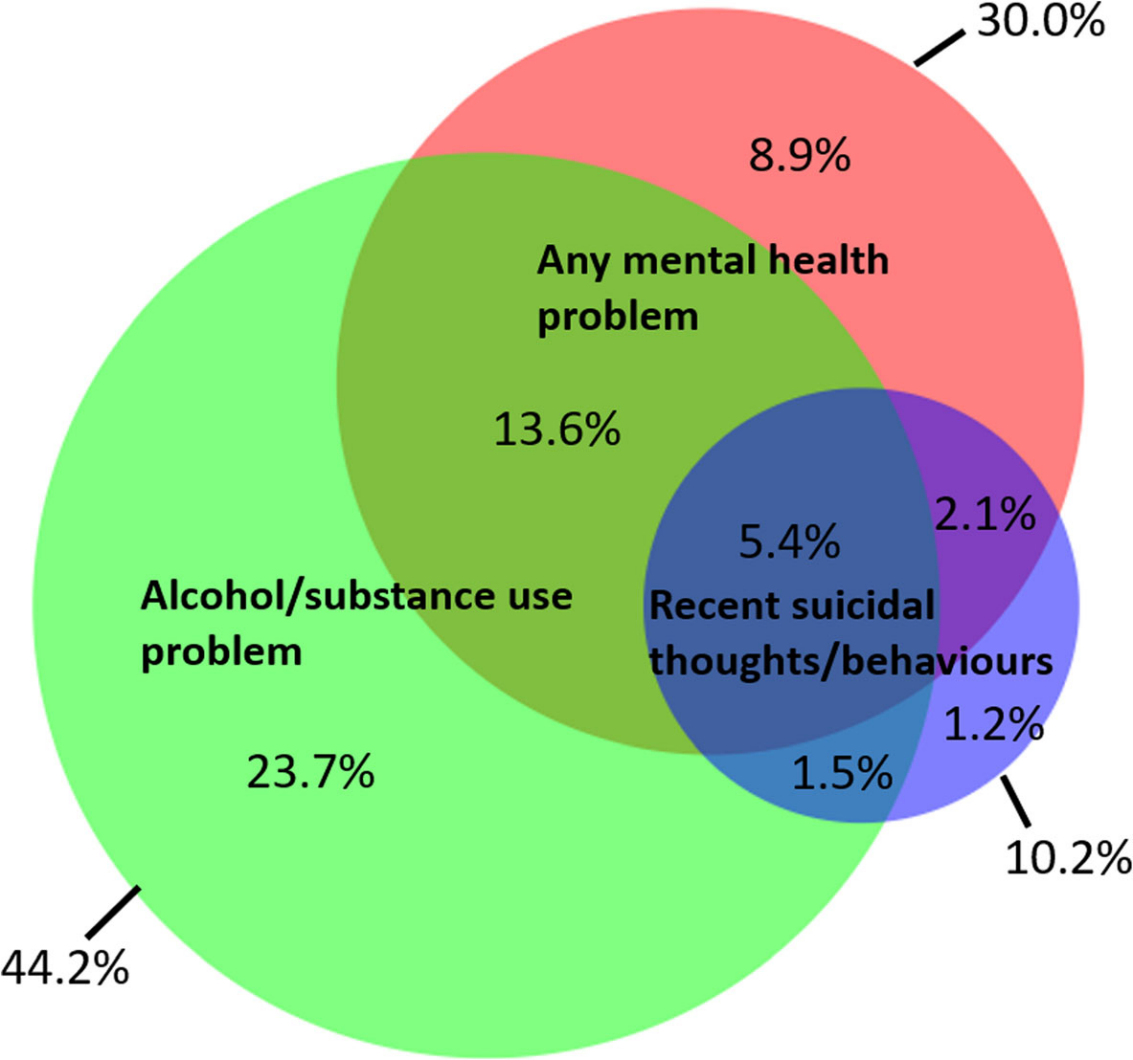
Overlap between any mental health problem, recent suicidal thoughts/behaviours and harmful alcohol/substance use

One in ten (10.2%; 95%CI: 8.5–12.2%) women reported recent (last 30 days) suicidal thoughts *and/or* behaviours (2.6% recent suicide attempt and 10.0% recent suicidal thoughts). Three quarters (75.5%) of participants with reported recent suicidal thoughts/behaviours had an underlying mental health problem *(*Fig. 3*).* Among women with any mental health problem (depression/anxiety/PTSD) almost two thirds (63.0%) also had a harmful alcohol/substance use problem. One in five women (20.5%) reported the co-occurrence of a mental health problem or suicidal thoughts/behaviours *and* an alcohol/substance use problem *(*Fig. 4).

### Associations with moderate/severe depression and/or anxiety

Women with moderate/severe depression and/or anxiety had a higher prevalence of ACEs (ACE score 9–12: 34.2% amongst women with depression/anxiety vs. 17.2% amongst all women) with strong evidence of an association (ACE score 9–12: aOR =7.00; 95%CI: 4.36–11.25; *p* < 0.001) in model 1*.* Specific ACEs associated with depression/anxiety included experience of sexual/physical violence and experience of war/community violence in childhood *(*Table 2*, model 1).* Older women > 35 (54.1% vs. 48.9% aOR: 2.08; 95%CI: 1.31–3.30; *p* = 0.007) were more likely to report depression and/or anxiety compared to younger women < 25.

**Table 2:** Multivariable logistic regression – associations with depression and/or anxiety

			Crude OR (95% CI)	Adjusted OR (95% CI)	*P*-value
*Model 1*^a^	Adverse family/household experience^b^		2.54 (1.45–4.48)	1.86 (1.03–3.36)	0.04
	Experience of war/community violence		2.74 (1.48–5.09)	1.89 (0.99–3.60)	0.05
	Experienced sexual or physical violence in childhood		3.06 (1.95–4.80)	2.06 (1.25–3.38)	0.004
	Experienced emotional abuse in childhood		2.35 (1.66–3.32)	1.53 (1.05–2.22)	0.03
	Street homeless as a child		1.73 (1.16–2.58)	1.45 (0.95–2.22)	0.08
	Number of ACEs	≤4	1.0	1.0	
		5–8	2.29 (1.52–3.44)	2.22 (1.47–3.34)	
		9–12	7.09 (4.46–11.3)	7.00 (4.36–11.25)	< 0.001
	Age	< 25	1.0	1.0	
		25–34	1.43 (0.94–2.18)	1.54 (0.97–2.46)	
		35+	1.76 (1.18–2.63)	2.08 (1.31–3.30)	0.007
*Model 2*	Additional income to sex work		1.49 (1.12–1.98)	1.76 (1.25–2.49)	0.001
	Hunger (skipped a meal in the last 7 days)		2.70 (2.01–3.62)	1.63 (1.13–2.36)	0.009
	Social support (someone to talk to about current problems)		0.66 (0.48–0.89)	0.59 (0.41–0.85)	0.005
	Reports death of a child		1.78 (1.21–2.68)	1.62 (1.00–2.62)	0.05
	Non-consensual first sex		2.29 (1.71–3.07)	1.90 (1.35–2.67)	< 0.001
	Perceived sex work stigma		4.13 (2.25–7.58)	3.44 (1.77–6.66)	< 0.001
*Model 3a*	Recent sexual and/or physical violence non IP		2.24 (1.65–3.03)	1.46 (1.00–2.15)	0.05
*Model 3b*	Alcohol use problem		4.04 (2.98–5.48)	2.67 (1.83–3.88)	< 0.001
	Other substance use problem		2.53 (1.89–3.40)	1.91 (1.29–2.81)	0.001

^a^Model 1 adjusted for level 1 variables (age, literacy, religion, socio-economic status) and clinic

Model 2 adjusted for all level 1 (cumulative ACEs, literacy, religion, SES) and level 2 variables (including marital status, no. of children, condom use at last sex with client, reported anal sex, place of selling sex, HIV status, any STIs, previous abortion/stillbirth).

Model 3a adjusted for level 1, 2 and 3a variables (including violence from IPs and arrest).

Model 3b adjusted for level 1, 2 and 3b variables.

^b^at least one of: household member or guardian depressed/suicidal, household member/guardian arrested, household member/guardian had an alcohol use problem, parental death, parental divorce/separation.

In model 2, proximal socio-economic factors associated with depression/anxiety included missing a meal in the last week due to lack of food (48.5% vs. 33.9%; aOR = 1.63; 95%CI: 1.13–2.36; *p* = 0.009) and reporting an additional income to sex work (50.9% vs. 43.7%; aOR = 1.76; 95%CI: 1.25–2.49; *p* = 0.001). Depression/anxiety was more common amongst women who reported having experienced the death of a child (aOR =1.62; 95%CI: 1.00–2.62; *p* = 0.05) and less common among women who reported social support (aOR = 0.59; 95%CI: 0.41–0.85; *p* = 0.005).

Women with depression/anxiety were more likely to report non-consensual sexual debut (48.2% vs. 31.3%; aOR = 1.90; 95%CI: 1.35–2.67; *p* < 0.001) and increased odds of perceived sex work stigma (95.3% vs. 86.0%; aOR = 3.44; 95%CI: 1.77–6.66; *p* < 0.001) in model 2. There was no evidence of an association with condom use or HIV/STIs.

Women with depression/anxiety were more likely to report recent physical and/or sexual violence from an a non-IP (69.2% vs. 55.0%; aOR: 1.46; 95%CI: 1.00–2.15; *p* = 0.05; model 3a) and had increased odds of harmful alcohol (aOR = 2.67; 95%CI: 1.83–3.88; *p* < 0.001; model 3b) or substance use (aOR = 1.91; 95%CI: 1.29–2.81; *p* = 0.001; model 3b) in adjusted analysis.

### Associations with PTSD

PTSD was also associated with a higher ACE score (ACE score 9–12: aOR = 7.19; 95%CI =3.84–13.45; *p* < 0.001) including having experienced sexual/physical violence and having lived on the street in childhood *(*Table 3*, model 1)*.

**Table 3:** Multivariable logistic regression – associations with PTSD

			Crude OR (95% CI)	Adjusted OR (95% CI)	*P*-value
*Model 1*^a^	Adverse family/household experience^b^		3.05 (1.33–7.01)	2.36 (1.01–5.52)	0.05
	Experienced sexual or physical violence in childhood		3.11 (1.70–5.68)	2.03 (1.03–3.99)	0.04
	Street homeless as a child		1.78 (1.11–2.86)	1.79 (1.08–2.95)	0.02
	Number of ACEs	≤4	1.0	1.0	
		5–8	2.85 (1.62–5.00)	2.92 (1.65–5.16)	
		9–12	6.34 (3.43–11.72)	7.19 (3.84–13.45)	< 0.001
*Model 2*	Additional income to sex work		1.65 (1.16–2.36)	1.68 (1.10–2.57)	0.02
	Hunger skipped a meal in the last 7 days		1.91 (1.33–2.73)	1.69 (1.05–2.72)	0.06
	Social support		0.58 (0.40–0.84)	0.59 (0.38–0.94)	0.03
	Children	None	1.0	1.0	
		1–2	0.48 (0.22–1.04)	0.31 (0.14–0.71)	
		3+	0.55 (0.25–1.24)	0.28 (0.11–0.73)	0.02
	Non-consensual first sex		3.40(2.37–4.89)	2.75 (1.77–4.28)	< 0.001
	Chlamydia prevalence		1.67 (0.89–3.15)	2.46 (1.15–5.27)	0.02
	Anal sex last 7 days		12.42 (4.47–34.55)	11.7 (3.62–37.59)	< 0.001
	Previous abortion/stillbirth		2.07 (1.44–2.99)	1.60 (1.03–2.49)	0.04
*Model 3a*	Recent sexual and/or physical violence non IP		2.42 (1.63–3.58)	1.72 (1.03–2.88)	0.04
	Recent sexual and/or physical violence from IP		2.22 (1.55–3.18)	1.68 (1.08–2.63)	0.02
*Model 3b*	Alcohol use problem		4.54 (3.14–6.56)	4.49 (2.73–7.36)	< 0.001

^a^Model 1 adjusted for level 1 variables (literacy, religion, socio-economic status) and clinic

Model 2 adjusted for level 1 (cumulative ACEs, literacy, religion, SES) and level 2 variables (including marital status, death of children, perceived sex work stigma, condom use at last sex with client, place of sex work, HIV status).

Model 3a adjusted for level 1, 2 and 3a variables (including violence from IPs and arrest).

Model 3b adjusted for level 1, 2 and 3b variables (including other substance use).

^b^at least one of: household member or guardian depressed/suicidal, household member/guardian arrested, household member/guardian had an alcohol use problem, parental death, parental divorce/separation.

In model 2, women with PTSD were also more likely to report recent hunger and an additional income to sex work *(*Table 3*).* PTSD was less common amongst women with social support (aOR = 0.59; 95% 0.38–0.94; *p* = 0.03) and amongst women who reported at least one child (1–2 children: aOR =0.31; 95%CI: 0.14–0.71; *p* = 0.02).

Women with PTSD reported increased prevalence of recent anal sex (7.6% vs. 1.6%; aOR =11.7; 95%CI: 3.62–37.59; *P* < 0.001) and chlamydia infection (8.4% vs 5.7%; aOR: 2.46; 95%CI: 1.15–5.27; *p* = 0.02) and were more likely to report having ever had an abortion/stillbirth (60.2% vs. 44.6%; aOR = 1.60; 95%CI: 1.03–2.49; *P* = 0.04).

Women with PTSD were more likely to report recent sexual and/or physical violence from IPs (46.4% vs. 30.9%; aOR = 1.68; 95%CI: 1.08–2.63; *p* = 0.02) and non-IPs (72.2% vs 55.0%; aOR = 1.72; 95%CI: 1.03–2.88; *p* = 0.04) in model 3a as well as increased odds of harmful alcohol use in model 3b.

### Associations with recent suicidal thoughts/behaviours

Recent suicidal thoughts/behaviours was associated with similar risk factors to depression/anxiety and PTSD including a higher ACE score (Table 4*, model 1),* additional income to sex work (*model 2*), recent violence from non-IPs (*model 3a*) and harmful alcohol and substance use (*model 3b)* in adjusted analysis. In addition, recent suicidal thoughts/behaviours was lowest amongst women in higher SES groups (upper-middle/upper: aOR =0.43; 95%CI: 0.25–0.76; *p* = 0.01) and amongst women with higher literacy levels (aOR =0.63; 95%C: 0.38–1.04; *p* = 0.07).

**Table 4:** Multivariable logistic regression – associations with recent suicidal thoughts/behaviours

			Crude OR (95% CI)	Adjusted OR (95% CI)	*P*-value
*Model 1*	Experienced emotional abuse in childhood		3.12 (1.76–5.53)	2.55 (1.32–4.92)	0.005
	Street homeless as a child		3.08 (1.90–4.99)	2.31 (1.41–3.81)	0.001
	Number of ACEs	≤4	1.0	1.0	
		5–8	1.51 (0.83–2.76)	1.40 (0.75–2.63)	
		9–12	6.13 (3.30–11.37)	5.41 (2.82–10.35)	< 0.001
	Literate	Yes	0.52 (0.33–0.84)	0.63 (0.38–1.04)	0.07
	Socioeconomic status	Low/low middle	1.0	1.0	
		Middle	0.66 (0.39–1.13)	0.73 (0.42–1.28)	
		Upper middle/upper	0.37 (0.22–0.61)	0.43 (0.25–0.76)	0.01
*Model 2*	Additional income to sex work		1.45 (0.96–2.18)	1.77 (1.09–2.88)	0.02
	Hunger (skipped a meal in the last 7 days)		2.86 (1.89–4.32)	1.72 (0.99–3.01)	0.06
	Social support		0.59 (0.39–0.90)	0.62 (0.36–1.04)	0.07
	Non-consensual first sex		2.24 (1.48–3.40)	1.77 (1.07–2.91)	0.03
	Children	None	1.0	1.0	< 0.001
		1–2	0.33 (0.14–0.77)	0.23 (0.09–0.61)	
		3+	0.66 (0.28–1.55)	0.30 (0.10–0.92)	0.01
*Model 3a*	Recent sexual and/or physical violence non IP		2.94 (1.83–4.73)	1.85 (1.01–3.41)	0.05
*Model 3b*	Alcohol use problem		2.85 (1.88–4.30)	1.72 (0.98–3.01)	0.06
	Other substance use problem		2.54 (1.69–3.83)	1.75 (1.02–3.01)	0.04

*Model 1 adjusted for level 1 variables (including age, literacy, religion, socio-economic status) and clinic

Model 2 adjusted for level 1 (cumulative ACEs, literacy, religion, SES) and level 2 variables (including marital status, death of children, perceived sex work stigma, condom use at last sex with client, recent anal sex, place of selling sex, HIV status, STIs, previous abortion/stillbirth).

Model 3a adjusted for level 1, 2 and 3a variables (including violence from IPs and arrest).

Model 3b adjusted for level 1, 2 and 3b variables

There was a strong association between recent suicidal thoughts/behaviours and an underlying mental health problem (depression/anxiety: AOR = 3.18; 1.79–5.67; *p* < 0.001 and PTSD: aOR = 3.71; 95%CI: 2.09–6.58; *p* < 0.001) in adjusted analysis.

## Discussion

We found a high prevalence of depression, anxiety, PTSD and recent suicidal thoughts/behaviours among FSWs in Nairobi. Common risk factors across the life course include ACEs, forced sexual debut, poverty (including low SES and hunger), harmful alcohol and substance use and recent violence experience. To our knowledge this is the first study among FSWs in a LMIC setting to examine associations between ACEs and mental health and suicidal thoughts/behaviour outcomes. We also found that social support may mitigate against the risk of mental health problems and suicidal thoughts/behaviours.

Our findings on the prevalence of mental health outcomes among FSWs are comparable to global estimates from a recent systematic review [8]. One quarter of women reported moderate/severe depression and approximately one in ten women reported moderate/severe anxiety, which is higher than among the general population in Kenya (depression: 4.4%, anxiety 3.1%) [34]. Previous suicidal thoughts/behaviours, reported by one in ten women in our study, have been shown in meta-analysis to significantly increase the lifetime risk of further suicide attempts and completed suicide [26, 27]. These findings emphasise the need for specific interventions to address mental health problems and suicide risk among FSWs.

A systematic review found that experiencing multiple ACEs was strongly associated with an increased risk of mental health problems in the general population, as well as a higher lifetime risk of violence and poor physical health outcomes [14]. In our study higher rates of ACEs were linked to an increased risk of mental health problems as well as having experienced specific ACEs; this was reflected in the Maisha Fiti qualitative interviews *(data not shown)*, with women reporting experiences of neglect, poverty and violence in childhood. Childhood experiences of violence have been shown to increase the risk of mental health problems [35] and the risk of re-victimisation and violence in adulthood [11, 36] in the general population. One previous study in the US found an association between PTSD and childhood physical and sexual violence [37] among FSWs. Experiences of trauma in childhood can impact brain development, affecting emotional stability and subsequent mental health outcomes [38]. Street homelessness and exposure to war and conflict in childhood, which were associated with mental health problems among FSWs in our study, have been shown in studies with non-FSW populations to affect mental health outcomes [39–42]. A recent systematic review of mental health problems in conflict settings reported that prevalence estimates of depression, PTSD, and anxiety are higher in conflict affected areas than amongst the general population [42]. In Kenya research on the micro-economic impacts of political unrest and civil conflict following the 2007 presidential election found that due to declines in income, there was an increase in women engaging in transactional sex during and after the conflict [43]. The pathways between ACEs and mental health outcomes are complex, particularly among women engaged in sex work. Another pathway to consider is that ACEs may drive women into sex work and sex work itself may increase the risk of poor mental health outcomes. This has been explored in a literature review which found that criminalisation, stigma, poor working conditions, isolation from peer and social networks, and financial insecurity negatively impact on sex workers’ mental health [44]. Longitudinal and qualitative findings from the Maisha Fit study will further explore the chronicity of these risk pathways across the life course.

We found that experiences of violence across the life course including sexual/physical violence in childhood, forced sexual debut and recent violence from IPs and clients, were strongly associated with mental health problems and recent suicidal thoughts/behaviours. This echoes findings from a recent systematic review among FSWs, reporting a pooled unadjusted OR between recent violence and depression of 2.3 (95%CI: 1.3–4.2) [45], and two previous studies among FSWs in South Africa [12] and Kenya [46] have linked recent violence and PTSD. Currently available studies among FSWs have been cross-sectional, but there is likely to be a bidirectional association between mental health problems and recent violence [47]. To our knowledge this is the first study among FSWs in a LMIC setting to examine violence at different stages of FSWs lives and their association with specific mental health problems and suicidal thoughts/behaviours.

We found a strong association between mental health problems, recent suicidal thoughts/behaviours and harmful alcohol/substance use. This is the first study among FSWs to find an association between PTSD and alcohol/substance use and to use a validated tool (ASSIST) [28] to measure substance use other than alcohol. This association is likely to be bi-directional, with harmful alcohol/substance use known to increase the risk of depression, anxiety and suicide risk [48], while mental health problems can drive alcohol/substance use as a coping mechanism [10]. These findings suggests that interventions to address mental health problems and harmful alcohol/substance use need to be designed in parallel.

Previous research has found associations between depression and HIV among FSWs, while we did not [45]. This is the first study among FSWs to have found an association between PTSD and increased STI prevalence (chlamydia infection) and sexual risk behaviours (recent anal sex). In the general population, there is evidence that poor mental health increases sexual risk behaviours [49, 50] and that treating mental health problems can reduce sexual risk behaviour and improve adherence to HIV care and treatment [51, 52]. We found that women with PTSD were more likely to report a previous abortion/stillbirth. Although this requires confirmation, it may relate to the fact that abortion is only legal in Kenya if the mother’s life is in danger, meaning that women may have resorted to illegal terminations, in secret with little or no support. There is evidence that forcing women to seek illegal terminations can have negative short and long term psycho-social impacts [53]. Our findings suggest that sexual and reproductive health services for FSWs need to address mental health as a key component of programming.

Poverty and low education were risk factors for mental health problems and suicidal thoughts/behaviours among FSWs. These socio-economic factors are known to be drivers of poor mental health among women in the general populations [54], but had not previously been examined among FSWs. The co-occurrence of multiple health and social risk factors, such as ACEs, poverty, violence, mental health and alcohol/substance use problems, which often perpetuate each other and contribute to increased burden of disease are known as syndemic factors [55, 56]. These syndemic risks create intergenerational cycles of poverty, adversity and poor mental health [14] and highlights the need for early childhood and family interventions. Our findings indicate that addressing or preventing adverse experiences in childhood through interventions which address psychological trauma, violence and homelessness would improve long term mental health outcomes Previous research from Kenya has found that community health workers can provide support to children who experience sexual abuse by helping them to report the abuse to police, to access healthcare services and alternative housing [57]. However lack of formal training and support from institutions often hindered them from providing optimal services to children, therefore improved training of community and other healthcare workers is a key area for policy makers to address. There are now an increasing number of interventions developed in Sub-Saharan Africa addressing parent–child relationships and child maltreatment, many focussing on tackling socio-cultural gender and sexual health norms. For example, in Rwanda, the MenCare+ programme, involved men in maternal and reproductive health, child health, couple communication, and violence prevention and the ‘Parenting for Respectability’ intervention with fathers and mothers in Kampala, Uganda addressed issues including alcohol and violence [58, 59]. Community and family programmes need to be complemented by structural interventions addressing the root causes of ACEs such as poverty – this will require political and economic changes which improve access to employment, housing and education.

We found evidence that social support may reduce the risk of mental health problems among FSWs. In addition, having children was found to reduce the risk of recent suicidal thoughts/behaviours, while loss of a child increased the risk of depression/anxiety. Previous qualitative research has shown that resilience factors such as social support can be protective against syndemic risks including substance use, mental health problems, violence re-victimisation, and HIV risk among FSWs [55, 60]. Interventions among FSWs should seek to understand and enhance resilience factors such as social support networks as these may empower women to break personal and intergenerational cycles of poor mental health.

### Strengths and limitations

A major strength of our study is the use of validated tools to assess mental health, alcohol/substance use, ACEs and violence, and qualitative interviews which were used to inform our conceptual pathways and triangulate our findings. Approximately 50% of the estimated FSW population in Nairobi are in active follow-up at one of the seven SWOP clinics, from where we drew our sample. Due to the stigmatised nature of sex work it is possible that the most vulnerable women may have been under-represented (as they have not visited a SWOP clinic). There was potential for recall bias and under-reporting of sensitive topics including ACEs, mental health, and alcohol/substance use as well as violence which was assessed at different time-points in women’s lives. Due to the heterogeneity in formal definitions of suicide risk and the small number of women reporting a recent suicide attempt there are limitations in our broad definition encompassing recent suicidal thoughts and behaviours. We did not have formal measurement tools for all variables including social support which reduces the reliability and validity of these measures. For exposures such as ACEs, we can generally assume the direction of association, however it is less clear for more proximal risk factors including sexual risk behaviours and recent violence. Longitudinal data will become available as the study progresses, enabling the directionality of association between exposures such as violence and mental health problems to be better ascertained.

## Conclusions

There are no published studies reporting mental health interventions for FSWs in LMICs and access to treatment for mental health problems for FSWs is limited. The high burden of mental health problems and suicidal thoughts/behaviours suggests that treatments are urgently needed, potentially embedded within existing HIV service provision. The identification of common risk factors across different mental health problems indicates key areas for developing focussed ‘upstream’ interventions which should aim to tackle structural and syndemic risk factors across the life course. These may include early childhood and family interventions to prevent the long term impact of ACEs as well as focussed interventions addressing violence and harmful alcohol/substance use as a key component of mental health programmes for FSWs.

## Supplementary Information

**Additional file 1.**

## Data Availability

The datasets generated and/or analysed during the current study are not publicly available as the study is still underway. However, the datasets will be available from the corresponding author from June 2023 (2 years after study data collection is completed).

## Abbreviations

A: Adverse Childhood Experiences
FSWs: Female Sex Workers
SWOP: Sex Worker Outreach Programme
PHQ-9: Patient Health Questionnaire
GAD-7: Generalised Anxiety Disorder tool
HTQ-17: Harvard Trauma Questionnaire
PTSD: Post-Traumatic Stress Disorder
ASSIST: Alcohol, Smoking and Substance Involvement Screening Test
SES: Socio-economic Status
LMIC: Low−/Low-Middle Income countries
HIV: Human Immunodeficiency Virus
STIs: Sexually Transmitted Infections
DSM-IV: Diagnostic and Statistical Manualof Mental Disorders 5th edition
IPV: Intimate Partner Violence
NG: *Neisseria Gonorrhoeae*
CT: *Chlamydia Trachomatis*
TV: Trichomonas vaginalis
BV: Bacterial Vaginosis
OR: Odds Ratios
CI: Confidence Interval
