# Supplementary material for: Prevalence and correlates of common mental health problems and recent suicidal thoughts and behaviours among female sex workers in Nairobi, Kenya
Source: BMC Psychiatry. 2021 Oct 14;21:503. doi: 10.1186/s12888-021-03515-5 (PMC8518166; doi:10.1186/s12888-021-03515-5)
Supplement: Supplementary file 1 — Additional file 1. [file 12888_2021_3515_MOESM1_ESM.doc]

**Supplementary File 1**

# **Prevalence and correlates of common mental health problems and recent suicidal thoughts and behaviours among female sex workers in Nairobi, Kenya**

Authors:

Alicja Beksinska^1^, Zaina Jama^2^, Rhoda Kabuti^2^, Mary Kungu^2^, Hellen Babu^2^, Emily Nyariki^2^, Pooja Shah^1^, Maisha Fiti Study Champions^2^, Chrispo Nyabuto^2^, Monica Okumu^2^, Anne Mahero^2^, Pauline Ngurukiri^2^, Erastus Irungu^2^, Wendy Adhiambo^2^, Peter Muthoga^2^, Rupert Kaul^3^, Janet Seeley^1^, Tara S. Beattie^1^, Helen A. Weiss^4^* Joshua Kimani^2^*

*Joint last authorship

Institutions

1. Department of Global Health and Development, London School of Hygiene and Tropical Medicine, London
2. UK Partners for Health and Development in Africa (PHDA), UNITID, College of Health Sciences, Nairobi, Kenya
3. University of Toronto, Toronto, Canada
4. MRC International Statistics & Epidemiology Group, Department of Infectious Disease Epidemiology, London School of Hygiene and Tropical Medicine, London

**Corresponding author details:**

Alicja Beksinska

Research Fellow

Department of Global Health and Development

London School of Hygiene and Tropical Medicine

Email: Alicja.Beksinska@nhs.net

**Keywords:** Mental Health, Female Sex Workers, Depression, Anxiety, Post-Traumatic Stress Disorder, Suicide, Keny

**
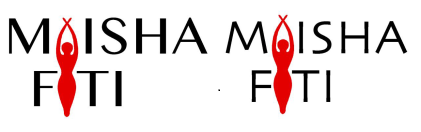
**

**The Maisha Fiti Study**

**Behavioural questionnaire for women in Nairobi, Kenya**

| SECTION I: PARTICIPANT INFORMATION |
| --- |
| Study number (Unique ID)…….………………………………………………………………..  Date of interview: Day Month Year  Name and code of interviewer: ________________________________________  ELIGIBILITY STATUS  Eligible 0  Not eligible (below 18yrs) 1 STOP  Not eligible (pregnant) 2 STOP  Not eligible (lactating) 3 STOP  CONSENT STATUS  Refused 0 STOP  Agreed for behavioural and biological but not hair sample 1 CONTINUE  Agreed for behavioural and biological including hair sample 2 CONTINUE    COMPLETION STATUS: BEHAVIOURAL  Completed interview 0  Did not complete interview 1    COMPLETION STATUS: BIOLOGICAL  Blood sample (Venipuncture) 0  Urine sample 1  Vaginal swabs x2 2  Vaginal SoftCup 3  Rapid HIV Test 4  COMPLETION STATUS: HAIR  Refused 0  Agreed 1    Language of the interview:___________________________________________________  Swahili=1, English=2, Other=3 |

| SECTION 2: BACKGROUND CHARACTERISTICS | | |
| --- | --- | --- |
| This questionnaire contains 14 sections. I am going to start by asking you some questions about your background.  **Dodoso hili lina sehemu kumi na nne. Nitaanza kwa kukuuliza maswali kuhusu historia yako.** | | |
|  | How old are you?  **Una umri wa miaka mingapi?** | Years  Decline to answer………………………….. 99 |
|  | What is your level of education?  **Kiwango cha juu cha elimu ulichofikisha ni kipi?** | Never attended school… 0  Attended primary school, but did not complete 1  Completed primary school 2  Attended secondary school, but did not complete3  Completed secondary school 4  Attended vocational school, college, or university after high school, but did not complete… 5  Completed vocational school, college or university after high school… …… 6  Other (specify):_________________________ 7  Decline to answer………………………….. 99 |
|  | Can you read this sentence?  The Quick brown fox jumps over the lazy dogs  **Je, unaweza soma sentensi hii?**  **Mwenda pole hajikwai** | No 0  Partially 1  Yes 2  Decline to answer………………………….. 99 |
|  | What is your religion?  **Dini yako ni ipi?**  ***If respondent answers “Christian,” ask***  ***“Are you Catholic or Protestant?”*** | Catholic 0  Protestants 1  Muslim………………………………… 2  No religion………………………………… 3  Other (specify)____________________­­­­_______4  Decline to answer………………………….. 99 |
|  | Which country were you born in?  **Ulizaliwa katika nchi gani?**  **DO NOT READ THE RESPONSE** | Kenya ……………………………………… 0  Ethiopia…………………………………….. 1  Tanzania…………………………………… 2  Uganda…………………………………….. 3  Somalia…………………………………….. 4  Burundi……………………………………... 5 Rwanda…………………………………….. 6  South sudan……………………………….. 7  Other (specify):___________________________8  Refuse to answer…………………………. 99 |
|  | Which sub-county do you currently live in in Nairobi?  **Unaishi katika kata (subcounty) ipi Nairobi?** | Embakasi Central………………………………… 0  Embakasi East………………………………… 1  Embasaki South………………………………… 2  Embakasi North………………………………… 3  Embakasi West………………………………… 4  Kamukunji………………………………… 5  Kasarani………………………………… 6  Kibra ………………………………… 7  Langata………………………………… 8  Makadara………………………………… 9  Mathare………………………………… 10  Roysambu………………………………… 11  Ruaraka ………………………………… 12  Starehe………………………………… 13  Westlands………………………………… 14  Dagoretti North……………………………………...15  Dagoretty South…………………………………….16  Other__________________________________97  Specify |
|  | How long have you been living in this sub-county?  **Umeishi kwa kata hii kwa miaka mingapi?**  ***Instruction: Record in years if greater than 12 months. Record in months* if less than 12 months** | Number of Months  Number of Years |
|  | Who are you currently living with?  **Unaishi na nani kwa wakati huu?**  **DO NOT READ RESPONSES**  **MULTIPLE ANSWERS** | Living alone………………………………. 0 **skip to 301**  Children………………………………. 1  Female friend or roommate (not sexual partner) 2  Male friend or roommate (not sexual partner) 3  Male sexual partner……………………………. 4  Female sexual partner……………………... 5  Parents………………..…………………… 6  Brother(s) and/or sister….……………. 7  Other family……………………………. 8  No answer ………………………………………….99 **skip to 301** |
|  | Not including yourself, how many adults currently live in your house with you (over the age of 18 years) and what is their gender?  **Kando na wewe, ni watu wangapi wazima (wenye Zaidi ya miaka 18) wanaishi na wewe na ni wa jinsia ipi?**  ***Instruction: Record number of other adults and also record gender*** | A. Number of other adults  B. Number of other female adults  C. Number of male adults |
|  | How many children (under the age of 18 years) currently live with you?  **Ni watoto wangapi (wenye chini ya miaka 18) wanaishi na wewe?** | Number of children |
|  | How many of these children are not your own children?  **Ni wangapi kati ya hawa si watoto wako?**  ***Instruction: i.e. how many children are children from other people that you have taken in to look after under your roof*** | Number of children |
|  | How many children (under the age of 6 years) currently live with you?  **Ni watoto wangapi chini ya miaka 6 wanaishi na wewe?**  **Check response with Q210** | Number of children <6 years |

| **SECTION 3. HOUSEHOLD INCOME / FINANCIAL STRESS** | | | | | | | |
| --- | --- | --- | --- | --- | --- | --- | --- |
| In this section I am going to ask you questions about your income and outgoings, for yourself and your household.  It’s important for the study that you answer these questions honestly. Anything you tell me will remain strictly confidential.  **Kwenye sehemu hii, nitakuuliza maswali kuhusu mapato na matumizi ya fedha kwa jamii yako. Ni muhimu kwa utafiti utupe majibu ya ukweli. Chochote utakachoniambia kitahifadhiwa kwa usiri mkuu.** | | | | | | | |
|  | In the last 3 months, what has been your main source of income?  **Kwa miezi mitatu iliyopita, kazi inayokupa mapato ya riziki imekuwa ipi?**  **Mark all that apply** | Sex work………………………………………….. 1  Business……….. ………………………………….. 2  Construction worker……………………………….. 3  Factory worker…………………………………… 4  Farming/agricultural worker……………………. 5  Government worker…………………………………6  Hairdresser/beautician/masseuse……………. 7  Shop worker……………………………………. 8  Street vendor/casual labourer…………………. 9  Tourism/travel agent/tour guide………………… 10  Waiter-waitress/bartender/hotel employee…..…..11  Parental/spousal support…………………………..12  OVC support…………………………………………13.  Other (specify):___________________________97 | | | | | |
|  | In the past month, how much was your income from all sources?  **Kwa mwezi uliopita, mapato yako kutoka shughuli/ kazi zote yalikuwa shilingi ngapi?** | Kshs  Don’t know…………………………………………..98  No answer……………………………………….. 99 | | | | | |
|  | In the past week, what has been your total income from all sources?  **Kwa wiki iliyopita, mapato yako yote yalikuwa shilingi ngapi?** | Kshs    Don’t know…………………………………………..98  No answer……………………………………….. 99 | | | | | |
|  | Not including yourself, how many people living in your household are dependent on your income?  **Bila kujihesabu, ni watu wangapi unaoishi na wao wanategemea mapato yako?** | Number of people  None…………………………… 00  No answer………………………………… 99 | | | | | |
|  | Not including yourself, how many people in your household are contributing financially?  **Bila kujihesabu, ni watu wangapi wanachangia kifedha nyumbani?** | Number of people  None…………………………… 00  No answer………………………………… 99 | | | | | |
|  | How many people living outside your household are dependent on your income?  **Ni watu wangapi usioishi na wao wanategemea mapato yako?**  **Instruction: by this we mean, how many people outside your household rely financially on you for money?** | Number of people  None…………………………… 00  No answer………………………………… 99 | | | | | |
|  | In the past 1 year, how many times have you moved house?  **Kwa mwaka mmoja uliopita, umehama mara ngapi kutoka kwa nyumba hadi nyingine?** | Number of times  None…………………………… 00  No answer………………………………… 99 | | | | | |
|  | Thinking about your current living situation, do you have a regular place where you stay and store your things?  **Ukifikiria unavyoishi, una pahali salama ambapo unaweza weka mali yako?** | No…………………………… ….0 **SKIP TO 318**  Yes………………………………. ….1  No answer…………………………………99 **SKIP TO 318** | | | | | |
|  | Thinking about the regular place where you currently stay and store your things, which of the following best describes the ***floor*** material of the building?  **Ukifikiria pahali hapo, ni ipi kati ya hizi inaelezea kwa karibu sakafu ya jengo hilo?** | Earth / Sand………………………………. 1  Dung………………………………………. 2  Wood ……………………………………... 3  Palm / bamboo…………………………… 4  Parquet or polished wood………………..5  Vinyl or asphalt strips…………………….6  Ceramic tiles……………………………… 7  Cement……………………………………. 8  Carpet…………………………………….. 9  Other (specify):____________________97 | | | | | |
|  | Thinking about the regular place where you currently stay and store your things, which of the following best describes the ***roof*** material of the building?  **Ukifikiria pahali hapo, ni ipi kati ya hizi inaelezea kwa karibu paa la jengo hilo?** | No roof………………………………. 1  Thatch / grass / makuti…………………. 2  Dung / mud / sod………………………... 3  Iron Sheets…..…………………………… 4  Tin cans…………………..………………..5  Asbestos sheet…………………………….6  Concrete…..……………………………… 7  Other (specify):____________________97 | | | | | |
|  | Thinking about the regular place where you currently stay and store your things, which of the following best describes the material of the external (outside) walls of the building?  **Ukifikiria pahali hapo, ni ipi kati ya hizi inaelezea kwa karibu kuta za jengo hilo?** | Cane / palm/ trunks………………………. 1  Dung / mud /sod…………………………. 2  Bamboo with mud………………………... 3  Stone with mud…………………………… 4  Uncovered abode………..………………..5  Plywood…………………………………….6  Cardboard………………………………… 7  Re-used wood……………………………. 8  Iron sheets.……………………………….. 9  Cement…………..………..………………..10  Stone with lime / cement..………….…….11  Bricks……………………………………… 12  Cement blocks……………………………. 13  Covered abode………………………….. 14  Wood plans / shingles………………….. 15  Other (specify):____________________97 | | | | | |
|  | Do you or a member of your household own this place, do you pay rent, or do you live here without paying rent?  **Pahali hapa panamilikiwa na familia/jamii yako, unalipa kodi ama unaishi pale bila kulipa kodi?** | Owns……………………………………. 1  Pays rent/lease………………………… 2  No rent with consent of owner……..…....3  No rent, squatting…………………..…… 4  Other (specify):____________________97 | | | | | |
|  | How many rooms are used for sleeping in this place?  **Kuna vyumba vingapi vya kulala pahali pale?** | Number of rooms | | | | | |
|  | What kind of toilet facility does your household use?  **Kuna choo cha aina ipi pahali pale?** | Own flush or pour flush toilet……………………. 1  Shared flush toilet………………………………. 2  Own pit latrine………………………………. 3  Shared pit latrine………………………………. 4  Composting toilet………………………………. 5  Bucket toilet ………………………………. 6  Hanging toilet / hanging latrine……………….…. 7  No facility/bush/field………………………. 8  Other (specify):__________________________97 | | | | | |
|  | What is the **main** source of lighting for  this place?  **Nini hutumika kuwapa mwangaza pahali pale?** | Electricity ................................................. 1  Kerosene ................................................. 2  Gas........................................................... 3  Other (specify):____________________97 | | | | | |
|  | What is the **main** type of fuel your household commonly uses for cooking?  **Nini hutumika kupikia nyumbani kwako?** | No cooking done in household…………………. 1  Electricity (mains) ………………………………. 2  Electricity (individual solar) ………………………. 3  LPG/ Natural Gas…………………………………. 4  Biogas………………………………………………..5  Paraffin/kerosene……………………..………. 6  Coal/Lignite……………………………………… 7  Charcoal………………………………….…. 8  Wood……………………………………….. 9  Straw/shrubs/grass…………………………….. 10  Agricultural crop…………………………….. 11  Animal Dung………………………………. 12  Other (specify):___________________________97 | | | | | |
|  | What is the **main** source of drinking water for the members of your household?  **Maji inayotumika kwako hutoka wapi?**  ***INSTRUCTION: IN TIMES OF DROUGHT, ASK WHAT WOULD USUALLY BE THEIR MAIN SOURCE OF DRINKING WATER***   |  |  | | --- | --- | | Piped into dwelling………………………………. 1  Piped to yard/plot……………………………….. 2  Communal tap/standpipe…………………………. 3  Tube well or Borehole………………………….. 4  Dug well ………………………………………….. 5  Water from spring……………………………….. 6  Rainwater………………………………. ……. 7  Tanker truck……………………………. ………..8  Cart with small tank……………………………….. 9  Surface water (river/dam/lake/stream)………….. 10  Bottled water………………….…………………….11  Other (specify):___________________________97 | | | | | |
|  | Do you or someone living in your household have:  **Nyumbani kwako kuna:**  **(READ EACH ITEM AND RECORD RESPONSES FOR EACH)** | **YES NO**   1. Electricity? .......................................... 1 0 2. A radio? .......................................... 1 0 3. A television? ....................................... 1 0 4. A mobile telephone?............................. 1 0 5. A non-mobile telephone?..................... 1 0 6. A refrigerator? ..................................... 1 0 7. A solar panel? ..................................... 1 0 8. A table? .......................................... 1 0 9. A chair? .......................................... 1 0 10. A sofa? .......................................... 1 0 11. A bed? .......................................... 1 0 12. A cupboard?......................................... 1 0 13. A clock? ...... .................................... 1 0 14. A microwave oven?.............................. 1 0 15. A DVD player? ..................................... 1 0 16. Stereo/cassette/other music player? ... 1 0 17. A motorcycle or scooter........................ 1 0 18. A car/truck? ..................................... 1 0 19. A bicycle? ... .................................. 1 0 20. A watch? ............. ........................ 1 0 21. A computer / laptop?............................. 1 0 22. Animal drawn cart?............................... 1 0 23. Boat with motor?.................................. 1 0 | | | | | |
|  | Does any member of your household own/lease any agricultural land?  **Kuna mtu yeyote kwa nyumba yako ambaye ako na au amekodisha shamba la kulima?** | No…………………………… 0  Yes………………………………. 1  Don’t know…………………………....98 | | | | | |
|  | Does your household own any livestock, herds, other farm animals, or poultry?  **Nyumbani kwako kuna wanyama wowote, mifugo ama kuku/ndege wa kufugwa?** | No…………………………… 0  Yes………………………………. 1  Don’t know…………………………....98 | | | | | |
|  | Does any member of your household have a bank account?  **Kuna mtu yeyote kwa nyumba yako anayemiliki akauinti kwa benki?** | No…………………………… 0  Yes………………………………. 1  Don’t know…………………………....98 | | | | | |
|  | Does your household receive a cash transfer or any social assistance from the government?  **Nyumba yako inapata fedha zozote ama usaidizi wowote kutoka kwa serikali?** | No…………………………… 0  Yes………………………………. 1  Don’t know…………………………....98 | | | | | |
| **Q323**    **A**  **B**  **C**  **D** | In the past 6 months, did you or anyone in your household have to….  **Kwa miezi sita iliyopita wewe ama mtu wa jamii yako...** | | | Yes | | No | No Answer |
|  | Borrow cash from friends because of lack of money?  **Alikopa pesa kutoka kwa Marafiki sababu hakuwa na pesa?** | | | 1 | | 0 | 99 |
|  | Borrow cash from shylocks because of lack of money?  **Alikopa pesa kutoka kwa Shylock sababu hakuwa na pesa?** | | | 1 | | 0 | 99 |
|  | Have to sell belongings, because of lack of money?  **Ilibidi auze mali yake ya nyumbani sababu hakuwa na pesa?** | | | 1 | | 0 | 99 |
|  | Borrow mobile cash because of lack of money?  **Alikopa pesa kutoka kwa simu sababu hakuwa na pesa?** | | | 1 | | 0 | 99 |
| **Q324** | Do you currently belong to any savings groups? If yes, how many?  **Uko katika kikundi chochote cha kuwekeza? Kama yuko, vikundi vingapi?** | No………………………………………….0 **SKIP TO 326**  Yes - one………………………………….1  Yes - two………………………………….2  Yes – three or more……………………..3  Don’t know………………………….......98 **SKIP TO 326**  No Answer………………………….......99 **SKIP TO 326** | | | | | |
| **Q325** | In the past month, how much money did you save as part of a savings group?  **Kwa mwezi uliopita, uliweka akiba ya pesa ngapi kwa kikundi cha kuwekeza?**  ***Instruction, in total how much money did you actually give to savings groups?*** | Kshs    Don’t know…………… 98 | | | | | |
| **Q326** | Nowadays, many families have a hard time making ends meet. I would like to learn more about how your household is coping.  **Siku hizi, familia nyingi hupata shida za kifedha, ningetaka kujua Zaidi vile unakabiliana na hizi shida kwa boma yako.**  ***Instruction, emphasize that you are talking about the past 3 months***  **In the past 3 months, how many times…….**  **Kwa miezi mitatu iliyopita, ni mara ngapi…….** | | | | | | |
|  | | Never | Once | | 2-3 times | 4 or more times | No answer |
| **a** | … were you very worried/stressed about your general financial situation.  **Ulipata wasiwasi (stress) kuhusu hali yako ya kifedha?** | 0 | 1 | | 2 | 3 | 99 |
|  | | Never | Once | | 2-3 times | 4 or more times | No answer |
| **b** | …have you had trouble buying food or other necessities for your family?  **Umeshindwa kununua chakula au kukimu mahitaji mengine ya familia yako?** | 0 | 1 | | 2 | 3 | 99 |
| **c** | … have you had to borrow money to pay rent or other bills ?  **Imebidi ukope pesa kulipia kodi ya ama bili nyingine?** | 0 | 1 | | 2 | 3 | 99 |
| **d** | … did any of your family members need to see a doctor but could not because you did not have enough money?  **Wewe ama mmoja wa jamii yako alihitaji kuona daktari lakini haungeweza sababu haukuwa na pesa ya kutosha?** | 0 | 1 | | 2 | 3 | 99 |
| **e** | … did your children miss days of school because you did not have money for school fees, uniforms or supplies eg pen ?  **Watoto wako walikosa kwenda shule sababu haukuwa na pesa ya karo ama sare au vifaa vingine kama vile kalamu?** | 0 | 1 | | 2 | 3 | 99 |
| **f** | … have you or any of your own children gone a whole day without eating anything because there was not enough food  **Watoto wako walishinda siku mzima bila kukula sababu hakukuwa na chakula cha kutosha?** | 0 | 1 | | 2 | 3 | 99 |
| **g** | …did you agree to have sex with someone you did not want to have sex with, or agree to have condomless sex, because you were financially stressed?    **Ulikubali kufanya ngono na mtu ambaye haukutaka ama ukakubali kufanya ngono bila mpira sababu ulikuwa unahitaji pesa?** | 0 | 1 | | 2 | 3 | 99 |
| **h** | Thinking now about the past 7 days, have you or anyone in your family skipped a meal because there was not enough food?  **Ukifikiria siku saba zilizopita, kuna mtu yeyote kwa jamii yako alikosa kula kwa sababu hakukuwa na chakula cha kutosha?** | No…………………… 0  Yes 1  No answer 99 | | | | | |

| **SECTION 4. FAMILY AND MARRIAGE** | | | |  |
| --- | --- | --- | --- | --- |
| **In this section, I’m going to ask you questions about your family and marriage**  **Kwenye sehemu hii, nitakuuliza maswali kuhusu familia na ndoa** | | | |  |
|  | Have you ever been married or cohabited/ lived with a sexual partner as if married?  **Umewahi kuolewa ama kuishi na mpenzi kana kwamba mmeoana?** | | No……………………………………0 **SKIP TO 403**  Yes………………………………….1  Don’t know…………………………98 **SKIP TO 403**  No answer…………………………99 **SKIP TO 403** |  |
|  | How old were you when you first married or cohabited/ lived with a sexual partner as if married?  **Ulikuwa na umri wa miaka mingapi ulipoolewa ama kuishi na mpenzi kana kwamba mmeoana?** | | Years  Other (specify):___________________________97 |  |
|  | What is your current marital status?  **Kwa wakati huu, hali yako ya ndoa ni ipi?** | | Single (never married)……………… 1  Married…………… 2  Cohabiting/living with a sexual partner….3  Separated………………………………….4  Divorced……………………………………5  Widowed…………………………… 6  Other (specify):____________________97  No answer……………………………… 99 |  |
|  | Have you ever conceived/been pregnant  **Umewahi pata mimba?** | | No……………………………………0 **SKIP TO 412**  Yes………………………………….1  Don’t know…………………………98 **SKIP TO 406**  No answer…………………………99 **SKIP TO 406** |  |
|  | How many times did you ever become pregnant?  **Umepata mimba mara ngapi?** | | Number of pregnancies  Don’t know…………………………98  No answer…………………………99 |  |
|  | How many children do you currently have?  **Kwa wakati huu, una watoto wangapi?** | | Number of children  No answer__________________ 99 |  |
|  | How many children have you had that were born living but have since died?  **Ni watoto wangapi umewahi pata lakini wakafariki?** | | Number of children: _______________  None…………………………… 00  No answer………………………………… 99 |  |
|  | What is the age of your eldest live child?  **Mtoto wako wa kwanza aliyehai ana miaka mingapi?** | | Years  **If only one child, skip to 410** |  |
|  | What is the age of your youngest alive child?  **Mtoto wako wa mwisho aliyehai ana miaka mingapi?** | | Years |  |
|  | Have you ever had a pregnancy that ended in still birth, spontaneous abortion or induced abortion?  **Umewahi pata mimba iliyoishia mtoto kuzaliwa akiwa amekufa ama kutolewa ama kuavya mimba?** | | No……………………………………0 **SKIP TO 412**  Yes………………………………….1  Don’t know…………………………98 **SKIP TO 412**  No answer…………………………99 **SKIP TO 412** |  |
|  | If there are, how many?  **Kama kunazo, ni mimba ngapi?** | | Number  No answer………………………………. 99 |  |
|  | Are you currently doing something or using any method to delay or avoid getting pregnant?  **Kwa wakati huu, unatumia njia zozote za kuzuia kupata mimba?** | | No……………………………………0 **SKIP TO 501**  Yes………………………………….1  Don’t know…………………………98 **SKIP TO 501**  No answer…………………………99 **SKIP TO 501** |  |
|  | What is the method you currently use to delay or avoid getting pregnant?  **Kwa wakati huu, unatumia njia gani kuzuia kupata mimba?**  **CROSS ALL THAT APPLY** | | Condoms 1  Birth control pill 2  IUD/copper coil 3  Injections 4  Implant 5  Female sterilization 6  Herbal methods 7  Natural method (safe days)………………8  Other (specify):____________________97  Don’t know 98  No answer………………………………. 99 |  |
|  | Where do you normally go for family planning services?  **Huwa unaenda wapi kwa huduma za kupanga uzazi?**  **DO NOT READ ANSWERS, RECORD ALL MENTIONED** | | SWOP clinic 1  Government hospital/clinic/health center 2  Pharmacy 3  Private clinic 4  Drop-in centre 5  Traditional healer 6  Other___________________________ 97  (Specify)  Don’t know 98 |  |
| SECTION 5. SEXUAL PRACTICES AND BEHAVIORS | | | | |
| In this section I am going to ask you questions about your sexual practices and behaviours. Again, I’d like to let you know that it is important for the study that you answer these questions honestly. And to reassure you that anything you tell me will remain strictly confidential. **Katika sehemu hii, nitakuuliza maswali kuhusu tabia na mienendo ya kingono. Ni muhimu kwa utafiti utupe majibu ya ukweli. Chochote utakachoniambia kitahifadhiwa kwa usiri mkuu.** | | | | |
|  | How old were you when you first had penile insertive vaginal sex with a male partner?  **Ulikuwa na umri wa miaka mingapi ulipofanya ngono ya kuma na mwanaume?** | Age in years  Don’t know………………………… 98  No answer …………………………… 99 | | |
|  | Who was the person with whom you first had sex?  **Mtu wa kwanza uliyefanya ngono naye alikuwa nani?** | Friend……… 1  Neighbour………………………… 2  Boyfriend……………………………… 3  Client…………………………………… 4  Spouse……………………………………..5  Family Member…………………………….6  Other(specify)_____________________ 97  No answer……………… 99 | | |
|  | Would you say you wanted to have sex that first time, or was it against your will?  **Unaweza sema ulikuwa unataka kufanya ngono mara hiyo ya kwanza ama ulilazimishwa**? | I wanted to have sex……………….…… 1  I was tricked into having sex………..… 2 I was pressured into having sex……… 3  I was was physically forced to have sex…4  Other (specify)_____________________97  No answer…………………………… 99 | | |
|  | How old were you when you first received money/goods in exchange for sex?  **Ulikuwa na miaka mingapi ulipopokea pesa ama bidhaa kwa mbadala wa ngono?** | Years  Don’t know………………………… 98  No answer……………………… 99 | | |
|  | How old were you when you started selling sex regularly?  **Ulikuwa na miaka mingapi ulipoanza kuuza ngono mara kwa mara.** | Years  Don’t know………………………… 98  No answer……………………… 99 | | |
|  | When was the last time you had vaginal sex with any man (client or partner)?  **Mara ya mwisho kushiriki ngono ya kuma na mwanaume yeyote (mteja ama mpenzi) ilikuwa lini?** | Within last 24 hours……………….…… 1  1-3 days ago……………….…… 2  4-6 days ago…………….…… 3  Within past 1 month …… 4  Within past 3 months……………………...5  Within past 6 months 6  >6 months ago……………….…… 7  Other (specify)____________________97  No answer……………….…… 99 | | |
|  | The last time you had vaginal sex, did you use a condom?  **Mara ya mwisho kushiriki ngono ya kuma, mpira wa kondomu ulitumika?** | No…………………… 0  Yes 1  Don’t know………………………… 98  No answer 99 | | |
|  | Where do you usually meet/ pickup/solicit your male clients?  **Huwa unapatana ama kutafuta wateja wa kiume wapi?**  **MULTIPLE RESPONSE, CROSS ALL MENTIONED** | Street………………………….. 1  Home………………………….. 2  Bus/taxi/truck stand…………. 3  Sex den/brothel.................... 4  Bar/club/restaurant....................... 5  Lodge/hotel…………………… 6  Escort services………………………… 7  Massage parlor………………. 8  Markets………………………… 9  Phone/mobile………………… 10  Social gatherings……………. 11  Through middleman…………. 12  Internet………………………… 12  Other specify)______________________97  No answer…………………………… 99 | | |
|  | Where do you usually have sex with your male clients  **Huwa unafanya ngono wapi na wateja wako wa kiume?** | Street………………………….. 1  Other public place (eg railway, park) 2  Home………………………….. 3  Bus/taxi/truck .…………. 4  Car…..……………………………………...5  Sex den/brothel.................... 6  Room at Bar/restaurant ...................... 7  Toilets at bar/restaurant…………………..8  Lodge/hotel…………………… 9  Other (specify)­­­­____________________97  No answer…………………………… 99 | | |
|  | How many clients did you have sexual intercourse with on the last day you worked?  **Siku ya mwisho uliuza ngono, ulifanya ngono na wateja wangapi?** | Number of clients  Don’t know …………………………… 98  No answer……………………………… 99 | | |
|  | How many clients did you have sexual intercourse with in the past one week (7 days)?  **Ulishiriki ngono na wateja wangapi kwa wiki moja iliyopita?** | Number of clients  Don’t know …………………………… 98  No answer……………………………… 99 | | |
|  | The last time you had sex with a client, what type of sex did you have?  **Mara ya mwisho ulifanya ngono na mteja, ulifanya ngono ya aina gani?**  **MULTIPLE RESPONSE, CROSS ALL MENTIONED** | Vaginal………………………………… 1  Anal……………………………………… 2  Oral……………………………………… 3  Other (specify)_____________________97  No answer…………………………… 99 | | |
|  | Did the client you last had vaginal sexual intercourse with use a condom?  **Mara ya mwisho ulipofanya ngono ya kuma na mteja, mlitumia mpira wa kondomu?** | No…………………………………………. 0  Yes………………………………………… 1  Don’t know……………………………….. 98  No answer………………………………... 99 | | |
|  | Have you had anal sex with a client in the past 6 months?  **Umefanya ngono ya mkundu na mteja kwa miezi sita iliyopita?** | No………………………………………… 0 **SKIP TO 518**  Yes…………………………………………1  Don’t know……………………………… 98 **SKIP TO 518**  No answer……………………………… 99 **SKIP TO 518** | | |
|  | Have you had anal sex with a client in the past 7 days?  **Umefanya ngono ya mkundu na mteja kwa siku saba zilizopita?** | No…………………………………………. 0  Yes………………………………………… 1  Don’t know……………………………… .. 98  No answer………………………………... 99 | | |
|  | Did the client you last had anal sexual intercourse with use a condom?  **Ulipofanya ngono ya mkundu na mteja mara ya mwisho, mlitumia mpira wa kondomu?** | No…………………………………………. 0  Yes………………………………………… 1  Don’t know……………………………….. 98  No answer………………………………... 99 | | |
|  | The last time you had anal sex with a client, did you use a lubricant?  **Ulipofanya ngono ya mkundu na mteja mara ya mwisho, mlitumia mafuta ya utelezi?** | No…………………………………………. 0  Yes………………………………………… 1  Don’t know……………………………….. 98  No answer………………………………... 99 | | |
| The next set of questions, I would like to ask you specifically about the casual male clients. Casual male clients mean clients who have come to you only once or twice and you do not know them.  **Kwa maswali yafutayo, ningetaka kukuuliza kuhusu wateja wa mara moja moja wanaume. Yaani, wateja wanaume ambao umefanya ngono na wao mara moja ama mbiili na hauwafahamu vizuri.** | | | | |
|  | Do you currently have casual clients?  **Uko na wateja wowote wa mara moja moja?** | No………………………………………0 **SKIP TO 525**  Yes……………………………………..1  No answer…………………………... 99 **SKIP TO 525** | | |
|  | Have you had sex with a casual client in the past 7 days?  **Umefanya ngono na mteja wa mara moja moja kwa siku saba zilizopita?** | No………………………… 0  Yes……………………….. 1  No answer………………… 99 | | |
|  | The last time you had vaginal sex with a casual client, did he use a condom?  **Mara ya mwisho ulipofanya ngono na mteja wa mara moja moja, ulitumia condom?** | No………………………… 0  Yes……………………….. 1  Don’t know……………………………….. 98  No answer………………… 99 | | |
|  | How often do your casual clients use condoms with you for vaginal sex?  **Ni mara ngapi ulitumia mpira na wateja wa mara moja?** | Everytime………………….. 1  Often……………………….. 2  Sometimes………………… 3  Never……………………….. 4  No answer………………… 99 | | |
|  | Have you had anal sex with a casual client in the past 7 days?  **Umefanya ngono ya mkundu na mteja wa mara moja kwa siku saba zilizopita?** | No………………………………………0 **SKIP TO 525**  Yes……………………………………..1  No answer…………………………... 99 **SKIP TO 525** | | |
|  | The last time you had anal sex with a  Casual client, did he use a condom?  **Mara ya mwisho ulipofanya ngono ya mkundu na mteja wa mara moja, ulitumia mpira?** | No………………………… 0  Yes……………………….. 1  Have not had anal sex with occ client…..3  No answer………………… 99 | | |
|  | The last time you had anal sex with a  Casual client, did you use lubricant?  **Mara ya mwisho ulipofanya ngono ya mkundu na mteja wa mara moja, ulitumia mafuta ya utelezi?** | No………………………… 0  Yes……………………….. 1  Have not had anal sex with occ client…..3  No answer………………… 99 | | |
| The next set of questions, I would like to ask you specifically about the regular male clients. Regular male client’s means clients you recognize well, who have come to you at least 3 or more times and/or you know them.  **Kwa maswali yafuatayo, ningepeda kukuuliza kuhusu wateja wa mara nyingi. Yaani, wateja ambao umefanya ngono nao Zaidi ya mara tatu na unawajua.** | | | | |
|  | Do you currently have regular clients?  **Uko na wateja wowote wa mara nyingi?** | No…………………………… 0 **SKIP TO 532**  Yes…………………………. 1 | | |
|  | Have you had sex with a regular client in the past 7 days?  **Umefanya ngono na mteja wa mara nyingi kwa siku saba zilizopita?** | No………………………… 0  Yes……………………….. 1  No answer………………… 99 | | |
|  | The last time you had vaginal sex with a  Regular client, did he use a condom?  **Mara ya mwisho uliposhiriki ngono ya kuma na mteja wa mara nyingi, ulitumia mpira wa kondomu?** | No………………………… 0  Yes……………………….. 1  Don’t know……………………………….. 98  No answer………………… 99 | | |
|  | How often do your regular clients use condoms with you for vaginal sex?  **Ni mara ngapi ulitumia mpira wa kondomu na wateja wa mara nyingi kushiriki ngono ya kuma?** | Everytime 1  Often 2  Sometimes 3  Never 4  No answer………………… 99 | | |
|  | Have you had anal sex with a regular client in the past 7 days?  **Umefanya ngono ya mkundu na mteja wa mara nyingi kwa siku saba zilizopita?** | No………………………… 0 **SKIP TO 532**  Yes……………………….. 1  No answer………………………………..99 **SKIP TO 532** | | |
|  | The last time you had anal sex with a regular client, did he use a condom?  **Mara ya mwisho ulipofanya ngono ya mkundu na mteja wa mara nyingi, ulitumia mpira?** | No………………………… 0  Yes……………………….. 1  Don’t know……………………………….. 98  No answer………………… 99 | | |
|  | The last time you had anal sex with a  regular client, did you use lubricant?  **Mara ya mwisho ulipofanya ngono ya mkundu na mteja wa mara nyingi, ulitumia mafuta ya utelezi?** | No………………………… 0  Yes……………………….. 1  Don’t know……………………………….. 98  No answer………………… 99 | | |
| The next sets of questions include both regular and casual male clients.  **Maswali yafuatayo ni kuhusu wateja wa mara moja moja na wateja wa mara mingi.** | | | | |
|  | The last time you had sex with a paying **male** client, how much money did you receive?  **Mara ya mwisho ulipofanya ngono na mteja mwanaume aliyekulipa, ulipokea pesa ngapi?** | [____|____|____|____|____] KSH (amount received)  Received another form of compensation (specify)  ________________________________ 97  Doesn’t remember………………… .98  No answer……………………………… 99 | | |
|  | In the last six months, how many times has a client refused or had to be forced to pay you for sex you have provided?  **Kwa miezi sita iliyopita, ni mara ngapi mteja amekataa ama amelazimishwa kukulipa baada ya ngono?** | Never...................................................... 0  Once....................................................... 1  2 to 5 Times ........................................... 2  6 or more times....................................... 3  Don’t know………………… .98  No answer……………………………… 99 | | |
|  | In the last 6 months, have you sold sex in any county outside of Nairobi?  **Umeuza ngono kaunti ingine nje ya Nairobi kwa miezi sita iliyopita?** | No………………………………..…………….0  Yes……………………………………….……1  Other (specify)_____________________97  Don’t know………………… .98  No answer……………………………… 99 | | |

| **SECTION 6. MAIN PARTNER** | | | | | | |
| --- | --- | --- | --- | --- | --- | --- |
| For the next set of questions, I would like to ask you specifically about your lovers or boyfriends who do not pay you for sex.  **Kwa maswali yafuatayo, ningependa kukuuliza hasa kuhusu wapenzi wako ambao huwa hawakulipi ili ushiriki** | | | | | | |
|  | Do you currently have any intimate partners (who are not paying clients)?  **Kwa wakati huu, una wapenzi wako ambao huwa hawakulipi ili ufanye ngono na wao?** | | | No…………………………….………….0 **SKIP TO 701**  Yes………….………………….………..1  Don’t know………………………………98  No answer………………………………99 | | |
|  | Thinking about the past one month, how many of these partners did you have sex with?  **Ukifikiria mwezi uliopita, ulifanya ngono na wangapi wa hawa wapenzi?** | | | One………………. 1  Two………………. 2  Three………………. 3  Four………………. 4  Other (specify)_____________________97  No answer……………. 99 | | |
|  | ***Instruction: complete Q603-618 for partner 1*** ***and then repeat for partner 2 and partner 3.***  Thinking about your intimate partner, how old is he?  **Ukifikiria mpenzi wako maalum (main), ako na miaka mingapi?** | | | Partner 1 | Partner 2 | Partner 3 |
|  | Do you know his HIV status?  **Unajua hali yake ya VVU?** | | Don’t know.…… .….........1  Last HIV test negative…..2  Last HIV test positive……3  No answer………………99  **(Mark answer in box)** | Partner 1 | Partner 2 | Partner 3 |
|  | How long have you been in a relationship with this partner?  **Umekuwa na uhusiano wa kimapenzi na yeye kwa muda upi?**  ***IF >12 MONTHS, RECORD IN YEARS, OTHERWISE RECORD IN MONTHS*** | | | Partner 1  Months  Years | Partner 2  Months  Years | Partner 3  Months  Years |
|  | Was this sexual partner a paying client before he became your partner?  **Huyu mpenzi alikuwa mteja wa kulipa kabla uanze uhusiano wa kimapenzi na yeye?**  No………………………….………..…………..0  Yes………………………….………..……...….1  No answer……………………………..…...…99 | | | Partner 1 | Partner 2 | Partner 3 |
|  | Do you and this sexual partner live together?  **Mnaishi pamoja na huyu mpenzi?**  No………………………….………..…………..0  Yes………………………….………..……...….1  No answer……………………………..…...…99 | | | Partner 1 | Partner 2 | Partner 3 |
|  | Do you and this sexual partner have children together?  **Wewe na huyu mpenzi mna watoto pamoja?**  No ………………………….………..…………..0  Yes………………………….………..……...….1  Don’t know……………………………..…...…98  No answer……………………………..…...…99 | | | Partner 1 | Partner 2 | Partner 3 |
|  | Does this partner know about your sex work?  **Huyu mpenzi anajua unauza ngono?**    No ………………………….………..…………..0  Possibly…………………………………………1  Yes………………………….………..……...….2  Don’t know……………………………..…...…98  No answer……………………………..…...…99 | | | Partner 1 | Partner 2 | Partner 3 |
|  | In the past one month, has this partner provided you with any financial support?  **Kwa mwezi mmoja uliopita, huyu mpenzi amekupatia usaidizi wowote wa kifedha?**  No .............................................. ............... 0  Yes some of the time……………………….. 1  Yes regularly………………………... ... 2  No answer ................................................ 99 | | | Partner 1 | Partner 2 | Partner 3 |
|  | In the past month, have you supported this partner financially (eg with food, clothing, or his other needs?)  **Kwa mwezi mmoja uliopita, umempatia huyu mpenzi usaidizi wowote kama vile chakula, nguo, ama mahitaji yoyote?**  No .............................................. ............... 0  Yes some of the time……………………….. 1  Yes regularly………………………... ... 2  No answer ................................................ 99 | | | Partner 1 | Partner 2 | Partner 3 |
|  | Do you receive love and affection from this partner?  **Huwa unapokea upendo na mapenzi kutoka kwa huyu mpenzi?**  No .............................................. ............... 0  Yes some of the time……………………….. 1  Yes regularly………………………... ... 2  No answer ................................................ 99 | | | Partner 1 | Partner 2 | Partner 3 |
|  | During the past one week, how many times did you have sexual intercourse with this partner?  **Kwa wiki moja iliyopita, ni mara ngapi umefanya ngono na huyu mpenzi?**  No answer…………………………….……. 99 | | | Partner 1  Number of times | Partner 2  Number of times | Partner 3  Number of times |
|  | The last time you had vaginal sex with this partner, did he use a condom?  **Mara ya mwisho ulipofanya ngono ya kuma na huyu mpenzi, alitumia mpira?**  No ………………………….………..…………..0  Yes………………………….………..……...….1  Don’t know……………………………..…...…98  No answer……………………………..…...…99 | | | Partner 1 | Partner 2 | Partner 3 |
|  | | Have you ever had anal sex with this partner?  **Umewahi fanya ngono ya mkundu na huyu mpenzi?**  No ………………………….………..…………..0  Yes………………………….………..……...….1  Don’t know……………………………..…...…98  No answer……………………………..…...…99  **If no, skip to 603 if she has other intimate partners**  **or skip to 701 if she has no other intimate partners** | | Partner 1 | Partner 2 | Partner 3 |
|  | Have you had anal sex with this partner in the past 7 days?  **Umefanya ngono ya mkundu na huyu mpenzi kwa siku 7 zilizopita?**  No ………………………….………..…………..0  Yes………………………….………..……...….1  Don’t know……………………………..…...…98  No answer……………………………..…...…99 | | | Partner 1 | Partner 2 | Partner 3 |
|  | The last time you had anal sex with this partner, did he use a condom?  **Mara ya mwisho ulipofanya ngono ya mkundu na huyu mpenzi, alitumia mpira?**  No ………………………….………..…………..0  Yes………………………….………..……...….1  Don’t know……………………………..…...…98  No answer……………………………..…...…99 | | | Partner 1 | Partner 2 | Partner 3 |
|  | The last time you had anal sex with this partner, did you use lubricant?  **Mara ya mwisho ulipofanya ngono ya mkundu na huyu mpenzi, alitumia mafuta ya utelezi?**  No ………………………….………..…………..0  Yes………………………….………..……...….1  Don’t know……………………………..…...…98  No answer……………………………..…...…99  ***Instruction: if more then one intimate partner, repeat Q603-618 for second and third partners.*** | | | Partner 1 | Partner 2 | Partner 3 |

| **SECTION 7. INTRAVAGINAL WASHING PRACTICES** | | |
| --- | --- | --- |
| **We know many sex workers wash inside their vaginas after sex.**  **Tunaelewa kuwa wauzaji ngono wengi huosha ndani ya kuma baada ya kushiriki ngono.** | | |
|  | In the past 30 days, have you cleaned **inside** the vagina (beyond the outside opening)?  **Kwa siku 30 zilizopita, umeosha ndani ya kuma?** | No…………………………0 **SKIP TO 703**  Yes……………………..…1  No answer………………. 99 |
|  | If yes, what did you clean with?  **Uliosha ukitumia nini?**  **Mark all that apply** | Water only……………………….. 1  Household soap or shower gel…………. 2  Household cleaners……………………… 3  Antiseptic solutions………………………..4  Vinegar ……………………….. 5  Lemon Juice……………………….. 6  Other (specify)____________________97  No answer………………. 99 |
|  | In the past 30 days, have you ever used a cloth, tissue, paper or cotton to wipe **inside** the vagina to remove fluids?  (does not include tampons or removal of menstrual blood)  **Kwa siku 30 zilizopita, umetumia kitambaa, pamba ama tissue kupanguza ndani ya kuma kando na vipengele vya kuzuia damu ya mwezi?** | No………………………… 0  Yes……………………….. 1  No answer………………. 99 |
|  | In the past 30 days, have you ever pushed or placed something **inside** the vagina before sexual intercourse to achieve a dry or tight sensation?  **Kwa siku 30 zilizopita, umejitia/kujiingiza kifaa chochote ndani ya kuma kabla ya ngono ili kukausha ama kufanya kuma kuwa kama imeijikaza Zaidi?** | No………………………… 0  Yes……………………….. 1  No answer………………. 99 |
|  | In the past 30 days, have you ever ingested (drinking, swallowing) substances perceived to affect the vagina and uterus. This includes the ingestion of substances/medicines to dry or lubricate the vagina.  **Kwa siku 30 zilizopita, umetumia dawa zozote ambazo zinasemekana zinauwezo kubadilisha kuma ama njia ya uzazi? Hii ni pamoja na dawa za kuongeza utelezi ama kufanya kuma kujikaza Zaidi.** | No………………………… 0  Yes……………………….. 1  No answer………………. 99 |
|  | In the past 30 days, have you ever ‘‘steamed’’ or ‘‘smoked’’ your vagina, by squatting or standing above a source of heat (fire, coals, hot rocks) on which water, herbs, or oils are placed to create steam or smoke?  **Kwa siku 30 zilizopita, umetumia moshi au mvuke kwa kuma yako kwa kuchutama juu ya moto(jiko, makaa au mawe moto) yaliyotiwa viungo na mafuta ili kutoa mvuke?** | No………………………… 0  Yes……………………….. 1  No answer………………. 99 |
|  | Have you ever had a surgical procedure used for modifying the vagina, or restoration of the hymen; including female genital circumcision, incision with insertion of substance into the lesion (scarification process, tattoos of the vulva or labia)?  **Je! Umewahi kuwa na utaratibu wa upasuaji uliotumika kwa kurekebisha uke, au kurejeshwa kwa hymen; ikiwa ni pamoja na kutahiriwa, kukatwa kwa kuingizwa dutu katika lezi ( utaratibu wa scarification, tattoos kwa vulva au labia)?** | No………………………… 0  Yes……………………….. 1  No answer………………. 99 |
|  | Do you have any piercings in your vulva or labia?  **Una vipini kwa kuma nje ama ndani?** | No……………………………………………0  No but I used to…………………………….1  Yes………………………………………….2  No answer………………. 99 |

| **SECTION 8. VIOLENCE** | | |
| --- | --- | --- |
| Many women tell us that they can sometimes have problems with their clients and others being violent. By violent I mean humiliating, scaring, beating, hitting, slapping, burning, choking, threatening with a knife (etc) or forcing a woman to have sexual intercourse with them, even though they don't want to.  **Wanawake wengi wanatuambia kwamba wanaweza wakati mwingine kuwa na matatizo na wateja wao na wengine kuwa na vurugu. Kwa vurugu ninamaanisha kuwadhalilisha, kuwapiga, kuwagonga, kupiga mateke, kupiga makofi, kuchoma, kuwanyonga, kutishia kwa kisu (nk) au kulazimisha mwanamke kufanya ngono na wao hata kama hawataki.** | | |
| **INTIMATE PARTNER VIOLENCE**  I would like to start by asking you some questions on experience of violence with your intimate (non-paying) partners. Later I will ask you about violence from others. We know that answering these questions can sometimes be difficult but we want to reassure you that anything you tell us will remain confidential and it is important for our study that you answer these questions honestly.  **Ningetaka kuanza kwa kukuuliza maswali kuhusu dhuluma na wapenzi wako maalum. Baadaye nitakuuliza kuhusu dhuluma kutoka kwa watu wengine. Ni muhimu kwa utafiti utupe majibu ya ukweli. Chochote utakachoniambia kitahifadhiwa kwa usiri mkuu.**  **Can you tell me, has an intimate partner ever…. ( If no current IP, assess for violence ever)**  **Naomba unieleze kama mpenzi wako maalum ashawai…** | | |
|  | **Has an intimate partner ever…** Said or done something to humiliate you in front of others?  **Mpenzi wako maalum ashawai sema ama kufanya kitu killicho kudhalilisha mbele ya watu?**  No……………………………..0 **Skip to Q802**  Yes……………………………1  No answer…..………………99 **Skip to Q802** | **Q801a**  If yes, how often has an intimate partner done this in the past 6 months?  **Kama ni ndio, kwa muda wa miezi 6 ililyopita, mpenzi wako amefanya hivi mara ngapi?**  Never……………………………………….0  Once………………………………………..1  Few Times………………………………….2  Many Times………………………………..4  No answer………………………………….99 |
|  | **Has an intimate partner ever…** Threatened to hurt or harm you or someone close to you?  **Mpenzi wako maalum ashawai tishia kukuumiza wewe ama wapendwa wako?**  No……………………………..0 **Skip to Q803**  Yes……………………………1  No answer…..………………99 **Skip to Q803** | **Q802a**  If yes, how often has an intimate partner done this in the past 6 months?  **Kama ni ndio, kwa muda wa miezi 6 ililyopita, mpenzi wako amefanya hivi mara ngapi?**  Never……………………………………….0  Once………………………………………..1  Few Times………………………………….2  Many Times………………………………..4  No answer………………………………….99 |
|  | **Has an intimate partner ever…** Insulted you repeatedly to make you feel bad about yourself?  **Mpenzi wako maalum ashawai kutusi ili kukufanya uhisi vibaya kujihusu?**  No……………………………..0 **Skip to Q804**  Yes……………………………1  No answer…..………………99 **Skip to Q804** | **Q803a**  If yes, how often has an intimate partner done this in the past 6 months?  **Kama ni ndio, kwa muda wa miezi 6 ililyopita, mpenzi wako amefanya hivi mara ngapi?**  Never……………………………………….0  Once………………………………………..1  Few Times………………………………….2  Many Times………………………………..4  No answer………………………………….99 |

|  | **Has an intimate partner ever…** Done things to scare or intimidate you on purpose, e.g., by the way of looking at you, by yelling or smashing things?  **Mpenzi wako maalum ashawai fanya jambo kukuhofisha kwa vile alikuangallia, kupiganisha vitu, ama kukuongelesha kwa sauti ya juu?**  No……………………………..0 **Skip to Q805**  Yes……………………………1  No answer…..………………99 **Skip to Q805** | **Q804a**  If yes, how often has an intimate partner done this in the past 6 months?  **Kama ni ndio, kwa muda wa miezi 6 ililyopita, mpenzi wako amefanya hivi mara ngapi?**  Never……………………………………….0  Once………………………………………..1  Few Times………………………………….2  Many Times………………………………..4  No answer………………………………….99 |
| --- | --- | --- |
|  | **Has an intimate partner ever…** Pushed you, shaken you, or thrown something at you?  **Mpenzi wako maalum ashawai kusukuma, kukutingisha, ama akakurushia kitu?**  No……………………………..0 **Skip to Q806**  Yes……………………………1  No answer…..………………99 **Skip to Q806** | **Q805a**  If yes, how often has an intimate partner done this in the past 6 months?  **Kama ni ndio, kwa muda wa miezi 6 ililyopita, mpenzi wako amefanya hivi mara ngapi?**  Never……………………………………….0  Once………………………………………..1  Few Times………………………………….2  Many Times………………………………..4  No answer………………………………….99 |
|  | **Has an intimate partner ever…** Slapped or shoved you?  **Mpenzi wako maalum ashawai kupiga kofi ama kukusukuma?**  No……………………………..0 **Skip to Q807**  Yes……………………………1  No answer…..………………99 **Skip to Q807** | **Q806a**  If yes, how often has an intimate partner done this in the past 6 months?  Kama ni ndio, kwa muda wa miezi 6 ililyopita, mpenzi wako amefanya hivi mara ngapi?  Never……………………………………….0  Once………………………………………..1  Few Times………………………………….2  Many Times………………………………..4  No answer………………………………….99 |
|  | **Has an intimate partner ever…** Hit you with his fist or something else that could hurt you?  **Mpenzi wako maalum ashawai kupiga ngumi ama na kifaa ambacho kingekuumiza?**  No……………………………..0 **Skip to Q808**  Yes……………………………1  No answer…..………………99 **Skip to Q808** | **Q807a**  If yes, how often has an intimate partner done this in the past 6 months?  **Kama ni ndio, kwa muda wa miezi 6 ililyopita, mpenzi wako amefanya hivi mara ngapi?**  Never……………………………………….0  Once………………………………………..1  Few Times………………………………….2  Many Times………………………………..4  No answer………………………………….99 |
|  | **Has an intimate partner ever…** Kicked you, dragged you or beat you up?  **Mpenzi wako maalum ashawai kupiga mateke, kukuvuta ama kukuchapa?**  No……………………………..0 **Skip to Q809**  Yes……………………………1  No answer…..………………99 **Skip to Q809** | **Q808a**  If yes, how often has an intimate partner done this in the past 6 months?  **Kama ni ndio, kwa muda wa miezi 6 ililyopita, mpenzi wako amefanya hivi mara ngapi?**  Never……………………………………….0  Once………………………………………..1  Few Times………………………………….2  Many Times………………………………..4  No answer………………………………….99 |

|  | **Has an intimate partner ever…** Tried to choke you or burn you on purpose  **Mpenzi wako maalum ashawai jaribu kukuchoma ama kukunyonga kimakusudi?**  No……………………………..0 **Skip to Q810**  Yes……………………………1  No answer…..………………99 **Skip to Q810** | | **Q809a**  If yes, how often has an intimate partner done this in the past 6 months?  **Kama ni ndio, kwa muda wa miezi 6 ililyopita, mpenzi wako amefanya hivi mara ngapi?**  Never……………………………………….0  Once………………………………………..1  Few Times………………………………….2  Many Times………………………………..4  No answer………………………………….99 | |
| --- | --- | --- | --- | --- |
|  | **Has an intimate partner ever…** Threatened to use or actually used a knife, gun or any other weapon?  **Mpenzi wako maalum ashawaitumia kisu, bunduki ama silaha ingine ama alitishia kuzitumia?**  No……………………………..0 **Skip to Q811**  Yes……………………………1  No answer…..………………99 **Skip to Q811** | | **Q810a**  If yes, how often has an intimate partner done this in the past 6 months?  **Kama ni ndio, kwa muda wa miezi 6 ililyopita, mpenzi wako amefanya hivi mara ngapi?**  Never……………………………………….0  Once………………………………………..1  Few Times………………………………….2  Many Times………………………………..4  No answer………………………………….99 | |
|  | **Has an intimate partner ever…** Physically forced you to have sex with him even when you did not want to?  **Mpenzi wako maalum ashawai kulazimisha kushiriki ngono naye hata kama haukutaka?**  No……………………………..0 **Skip to Q812**  Yes……………………………1  No answer…..………………99 **Skip to Q812** | | **Q811a**  If yes, how often has an intimate partner done this in the past 6 months?  **Kama ni ndio, kwa muda wa miezi 6 ililyopita, mpenzi wako amefanya hivi mara ngapi?**  Never……………………………………….0  Once………………………………………..1  Few Times………………………………….2  Many Times………………………………..4  No answer………………………………….99 | |
|  | **Has an intimate partner ever…** Used threats of violence or rejection to force you to have sex with him when you did not want to?  **Mpenzi wako maalum ashawai tishia kukuchapa ama kukuacha usiposhiriki ngono na yeye?**  No……………………………..0 **Skip to Q813**  Yes……………………………1  No answer…..………………99 **Skip to Q813** | | **Q812a**  If yes, how often has an intimate partner done this in the past 6 months?  **Kama ni ndio, kwa muda wa miezi 6 ililyopita, mpenzi wako amefanya hivi mara ngapi?**  Never……………………………………….0  Once………………………………………..1  Few Times………………………………….2  Many Times………………………………..4  No answer………………………………….99 | |
|  | **Has an intimate partner ever…** Forced you to do something sexual that you found degrading or humiliating  **Mpenzi wako maalum ashawai kulazimisha kufanya kitu ulicho hisi kilikudhalilisha wakati wa kufanya ngono.**  No……………………………..0 **Skip to Q814**  Yes……………………………1  No answer…..………………99 **Skip to Q814** | | **Q813a**  If yes, how often has an intimate partner done this in the past 6 months?  **Kama ni ndio, kwa muda wa miezi 6 ililyopita, mpenzi wako amefanya hivi mara ngapi?**  Never……………………………………….0  Once………………………………………..1  Few Times………………………………….2  Many Times………………………………..4  No answer………………………………….99 | |
|  | Has there been a time in the past 7 days when you were physically forced to have sex with your intimate partner even when you did not want to?  **Kwa siku saba zilizopita, kuna mtu aliye mpenzi wako maalum alikulazimisha kufanya ngono na yeye?** | | No............................................ 0  Yes.......................................... 1  Don’t know / don’t remember. 98  No answer.............................. 99 | |
|  | Thinking about the violence you have experienced from an intimate partner, did your children under 18 years ever witness this violence against you?  **Ukifikiria kuhusu aina yoyote ya vita umepitia kutoka kwa mpenzi wako haswa kwa miezi sita iliyopita, kuna wakati watoto wako walio chini ya miaka 18 walikuweko na kushuhudia?** | | No………………………… 0 **Skip to Q817**  Not applicable (no violence / children)… 1 **Skip to Q817**  Yes……………………………………..... 2  Don’t know……………… 98 **Skip to Q817**  No answer………………………... 99 **Skip to Q817** | |
|  | Did your children under 18 years witness violence against you from an intimate partner in the past 6 months?  **Ukifikiria kuhusu aina yoyote ya vita umepitia kutoka kwa mpenzi wako maalum haswa kwa miezi sita iliyopita, kuna wakati watoto wako walio chini ya miaka 18 walikuweko na kushuhudia?** | | Yes……………………………………..... 1  No………………………… 2  Not applicable (no violence / children)… 3  Don’t know……………… 98  No answer………………………... 99 | |
|  | In the last six months how many times has your intimate partner drugged you?  **Kwa miezi sita iliyopita, ni mara ngapi mpenzi wako maalum amekuwekea dawa/mchele?**  ***Instruction: Put something in your drink or food which has meant you do not remember or know what happened to you.*** | | Never.. ................................................... 0  Once....................................................... 1  Few times............................................... 2  Many times……….................................. 3  Not applicable (no Intimate Partner)… 97  Don’t know……………………………..…. 98  No answer............................................... 99 | |
|  | In the last six months how many times has your intimate partner imprisoned you?  **Kwa miezi sita iliyopita, ni mara ngapi mpenzi wako maalum amekufungia kinyume na hiari yako?**  ***Instruction: Locked you up somewhere against your will?*** | | Never.. ................................................... 0  Once...................................................... 1  Few times.............................................. 2  Many times………................................. 3  Not applicable (no Intimate Partner)… 97  Don’t know. ……………………..………98  No answer........................................... 99 | |
| **VIOLENCE FROM PEOPLE OTHER THAN AN INTIMATE PARTNER** | | | | |
| We know that women can also experience violence from people other than an intimate partner, including clients, the police, city askaris, pimps, madams, other sex workers, landlords, strangers, goons, and family members.These next questions are going to be about violence you have experienced from anyone who is not an intimate partner.  **Tunajua kwamba wanawake hupitia dhuluma kutoka kwa watu wasio wapenzi wao kama polisi, wateja, kanjo, majangili na wauzaji ngono wenzao. Maswali yafuatayo yatakuwa juu ya vurugu uliyopata kutoka kwa mtu yeyote ambaye si mpenzi wa karibu.** | | | | |
|  | Has someone other than an intimate partner ever….  Said or done something to humiliate you in front of others?  **Kuna wakati mtu (isipokuwa mpenzi wa karibu) ashawai...**  **kusema ama kufanya kitu killicho kudhalalisha mbele ya watu?**  No………………..0 **Skip to Q820**  Yes………………1  No answer… …99 **Skip to Q820** | **Q819a**  In the past 6 months, how many times has this happened?  **Kwa miezi sita iliyopita, jambo hili limefanyika mara ngapi?**  Never…………0 **Skip to Q820**  Once…………….1  Few Times………2  Many Times…….3  No answer…….99 **Skip to Q820** | | **Q819b**  Who did this to you (past 6 months)?  **Nani alifanya hivi? (miezi 6 iliyopita)**  **(record all mentioned)**  Police............................................ 1  City askari…………………………. 2  Client…......................................... 3  Pimp/Madam................................. 4  Another SW.................................. 5  Stranger........................................ 6  Goon....... ……............................. 7  Family member (not IP)………..... 8  Friend………….…………………...9  Someone in community………... 10  Other (specify)______________97  No answer………………………..…99 |

|  | Has someone other than an intimate partner ever….Threatened to hurt or harm you or someone close to you?  **Kuna wakati mtu (isipokuwa mpenzi wa karibu) ashawai...**  **kutishia kukuumiza wewe ama wapendwa wako?**  No………………..0 **Skip to Q821**  Yes………………1  No answer… …99 **Skip to Q821** | **Q820a**  In the past 6 months, how many times has this happened?  **Kwa miezi sita iliyopita, jambo hili limefanyika mara ngapi?**  Never…………0 **Skip to Q821**  Once…………….1  Few Times………2  Many Times…….3  No answer…….99 **Skip to Q821** | **Q820b**  Who did this to you (past 6 months)?  **Nani alifanya hivi? (miezi 6 iliyopita)**  **(record all mentioned)**  Police............................................ 1  City askari…………………………. 2  Client…......................................... 3  Pimp/Madam................................. 4  Another SW.................................. 5  Stranger........................................ 6  Goon....... ……............................. 7  Family member (not IP)………..... 8  Friend………….…………………...9  Someone in community………... 10  Other (specify)______________97  No answer………………………..…99 |
| --- | --- | --- | --- |
|  | Has someone other than an intimate partner ever….Insulted you repeatedly to make you feel bad about yourself?  **Kuna wakati mtu (isipokuwa mpenzi wa karibu) ashawai...**  **kutusi ili kukufanya uhisi vibaya kujihusu?**  No………………..0 **Skip to Q822**  Yes………………1  No answer… …99 **Skip to Q822** | **Q821a**  In the past 6 months, how many times has this happened?  **Kwa miezi sita iliyopita, jambo hili limefanyika mara ngapi?**  Never…………0 **Skip to Q822**  Once…………….1  Few Times………2  Many Times…….3  No answer…….99 **Skip to Q822** | **Q821b**  Who did this to you (past 6 months)?  **Nani alifanya hivi? (miezi 6 iliyopita)**  **(record all mentioned)**  Police............................................ 1  City askari…………………………. 2  Client…......................................... 3  Pimp/Madam................................. 4  Another SW.................................. 5  Stranger........................................ 6  Goon....... ……............................. 7  Family member (not IP)………..... 8  Friend………….…………………...9  Someone in community………... 10  Other (specify)______________97  No answer………………………..…99 |
|  | Has someone other than an IP ever….Done things to scare or intimidate you on purpose, e.g., by the way of looking at you, by yelling or smashing things?  **Kuna wakati mtu (isipokuwa mpenzi wa karibu) ashawai...**  **kufanya jambo kukuhofisha kwa vile alikuangallia, kupiganisha vitu, ama kukuongelesha kwa sauti ya juu?**  No………………..0 **Skip to Q823**  Yes………………1  No answer… …99 **Skip to Q823** | **Q822a**  In the past 6 months, how many times has this happened?  **Kwa miezi sita iliyopita, jambo hili limefanyika mara ngapi?**  Never…………0 **Skip to Q823**  Once…………….1  Few Times………2  Many Times…….3  No answer…….99 **Skip to Q823** | **Q822b**  Who did this to you (past 6 months)?  **Nani alifanya hivi? (miezi 6 iliyopita)**  **(record all mentioned)**  Police............................................ 1  City askari…………………………. 2  Client…......................................... 3  Pimp/Madam................................. 4  Another SW.................................. 5  Stranger........................................ 6  Goon....... ……............................. 7  Family member (not IP)………..... 8  Friend………….…………………...9  Someone in community………... 10  Other (specify)______________97  No answer………………………..…99 |

|  | Has someone other than an intimate partner ever….Pushed you, shaken you, or thrown something at you?  **Kuna wakati mtu (isipokuwa mpenzi wa karibu) ashawai...**  **Kukusukuma, kukutingisha, ama akakurushia kitu?**  No………………..0 **Skip to Q824**  Yes………………1  No answer… …99 **Skip to Q824** | **Q823a**  In the past 6 months, how many times has this happened?  **Kwa miezi sita iliyopita, jambo hili limefanyika mara ngapi?**  Never…………0 **Skip to Q824**  Once…………….1  Few Times………2  Many Times…….3  No answer…….99 **Skip to Q824** | **Q823b**  Who did this to you (past 6 months)?  **Nani alifanya hivi? (miezi 6 iliyopita)**  **(record all mentioned)**  Police............................................ 1  City askari…………………………. 2  Client…......................................... 3  Pimp/Madam................................. 4  Another SW.................................. 5  Stranger........................................ 6  Goon....... ……............................. 7  Family member (not IP)………..... 8  Friend………….…………………...9  Someone in community………... 10  Other (specify)______________97  No answer………………………..…99 |
| --- | --- | --- | --- |
|  | Has someone other than an intimate partner ever….Slapped or shoved you?  **Kuna wakati mtu (isipokuwa mpenzi wa karibu) ashawai...**  **kukupiga kofi ama kukusukuma?**  No………………..0 **Skip to Q825**  Yes………………1  No answer… …99 **Skip to Q825** | **Q824a**  In the past 6 months, how many times has this happened?  **Kwa miezi sita iliyopita, jambo hili limefanyika mara ngapi?**  Never…………0 **Skip to Q825**  Once…………….1  Few Times………2  Many Times…….3  No answer…….99 **Skip to Q825** | **Q824b**  Who did this to you (past 6 months)?  **Nani alifanya hivi? (miezi 6 iliyopita)**  **(record all mentioned)**  Police............................................ 1  City askari…………………………. 2  Client…......................................... 3  Pimp/Madam................................. 4  Another SW.................................. 5  Stranger........................................ 6  Goon....... ……............................. 7  Family member (not IP)………..... 8  Friend………….…………………...9  Someone in community………... 10  Other (specify)_________  _____97  No answer………………………..…99 |
|  | Has someone other than an intimate partner ever….Hit you with his fist or something else that could hurt you?  **Kuna wakati mtu (isipokuwa mpenzi wa karibu) ashawai...**  **kupiga ngumi ama na kifaa ambacho kingekuumiza?**  No………………..0 **Skip to Q826**  Yes………………1  No answer… …99 **Skip to Q826** | **Q825a**  In the past 6 months, how many times has this happened?  **Kwa miezi sita iliyopita, jambo hili limefanyika mara ngapi?**  Never…………0 **Skip to Q826**  Once…………….1  Few Times………2  Many Times…….3  No answer…….99 **Skip to Q826** | **Q825b**  Who did this to you (past 6 months)?  **Nani alifanya hivi? (miezi 6 iliyopita)**  **(record all mentioned)**  Police............................................ 1  City askari…………………………. 2  Client…......................................... 3  Pimp/Madam................................. 4  Another SW.................................. 5  Stranger........................................ 6  Goon....... ……............................. 7  Family member (not IP)………..... 8  Friend………….…………………...9  Someone in community………... 10  Other (specify)______________97  No answer………………………..…99 |

|  | Has someone other than an intimate partner ever….Kicked you, dragged you or beat you up?  **Kuna wakati mtu (isipokuwa mpenzi wa karibu) ashawai...**  **kupiga mateke, kukuvuta ama kukuchapa?**  No………………..0 **Skip to Q827**  Yes………………1  No answer… …99 **Skip to Q827** | **Q826a**  In the past 6 months, how many times has this happened?  **Kwa miezi sita iliyopita, jambo hili limefanyika mara ngapi?**  Never…………0 **Skip to Q827**  Once…………….1  Few Times………2  Many Times…….3  No answer…….99 **Skip to Q827** | **Q826b**  Who did this to you (past 6 months)?  **Nani alifanya hivi? (miezi 6 iliyopita)**  **(record all mentioned)**  Police............................................ 1  City askari…………………………. 2  Client…......................................... 3  Pimp/Madam................................. 4  Another SW.................................. 5  Stranger........................................ 6  Goon....... ……............................. 7  Family member (not IP)………..... 8  Friend………….…………………...9  Someone in community………... 10  Other (specify)______________97  No answer………………………..…99 |
| --- | --- | --- | --- |
|  | Has someone other than an intimate partner ever….Tried to choke you or burn you on purpose  **Kuna wakati mtu (isipokuwa mpenzi wa karibu) ashawai...**  **jaribu kukuchoma ama kukunyonga kimaksudi?**  No………………..0 **Skip to Q828**  Yes………………1  No answer… …99 **Skip to Q828** | **Q827a**  In the past 6 months, how many times has this happened?  **Kwa miezi sita iliyopita, jambo hili limefanyika mara ngapi?**  Never…………0 **Skip to Q828**  Once…………….1  Few Times………2  Many Times…….3  No answer…….99 **Skip to Q828** | **Q827b**  Who did this to you (past 6 months)?  **Nani alifanya hivi? (miezi 6 iliyopita)**  **(record all mentioned)**  Police............................................ 1  City askari…………………………. 2  Client…......................................... 3  Pimp/Madam................................. 4  Another SW.................................. 5  Stranger........................................ 6  Goon....... ……............................. 7  Family member (not IP)………..... 8  Friend………….…………………...9  Someone in community………... 10  Other (specify)______________97  No answer………………………..…99 |
|  | Has someone other than an intimate partner ever….Threatened to use or actually used a knife, gun or any other weapon?  **Kuna wakati mtu (isipokuwa mpenzi wa karibu) ashawai...**  **kutishia ama alitumia kisu, bunduki ama silaha yoyote ile?**  No………………..0 **Skip to Q829**  Yes………………1  No answer… …99 **Skip to Q829** | **Q828a**  In the past 6 months, how many times has this happened?  **Kwa miezi sita iliyopita, jambo hili limefanyika mara ngapi?**  Never…………0 **Skip to Q829**  Once…………….1  Few Times………2  Many Times…….3  No answer…….99 **Skip to Q829** | **Q828b**  Who did this to you (past 6 months)?  **Nani alifanya hivi? (miezi 6 iliyopita)**  **(record all mentioned)**  Police............................................ 1  City askari…………………………. 2  Client…......................................... 3  Pimp/Madam................................. 4  Another SW.................................. 5  Stranger........................................ 6  Goon....... ……............................. 7  Family member (not IP)………..... 8  Friend………….…………………...9  Someone in community………... 10  Other (specify)______________97  No answer………………………..…99 |

|  | Has someone other than an intimate partner ever….Physically forced you to have sex with him even when you did not want to?  **Kuna wakati mtu (isipokuwa mpenzi wa karibu) ashawai...**  **kulazimisha kufanya ngono naye hata kama haukutaka?**  No………………..0 **Skip to Q830**  Yes………………1  No answer… …99 **Skip to Q830** | **Q829a**  In the past 6 months, how many times has this happened?  **Kwa miezi sita iliyopita, jambo hili limefanyika mara ngapi?**  Never…………0 **Skip to Q830**  Once…………….1  Few Times………2  Many Times…….3  No answer…….99 **Skip to Q830** | **Q829b**  Who did this to you (past 6 months)?  **Nani alifanya hivi? (miezi 6 iliyopita)**  **(record all mentioned)**  Police............................................ 1  City askari…………………………. 2  Client…......................................... 3  Pimp/Madam................................. 4  Another SW.................................. 5  Stranger........................................ 6  Goon....... ……............................. 7  Family member (not IP)………..... 8  Friend………….…………………...9  Someone in community………... 10  Other (specify)______________97  No answer………………………..…99 |
| --- | --- | --- | --- |
|  | Has someone other than an intimate partner ever….Used threats of violence or rejection to force you to have sex with him when you did not want to?  **Kuna wakati mtu (isipokuwa mpenzi wa karibu) ashawai...**  **kutishia kukuchapa ama kukuacha usipofanya ngono na yeye?**  No………………..0 **Skip to Q831**  Yes………………1  No answer… …99 **Skip to Q831** | **Q830a**  In the past 6 months, how many times has this happened?  **Kwa miezi sita iliyopita, jambo hili limefanyika mara ngapi?**  Never…………0 **Skip to Q831**  Once…………….1  Few Times………2  Many Times…….3  No answer…….99 **Skip to Q831** | **Q830b**  Who did this to you (past 6 months)?  **Nani alifanya hivi? (miezi 6 iliyopita)**  **(record all mentioned)**  Police............................................ 1  City askari…………………………. 2  Client…......................................... 3  Pimp/Madam................................. 4  Another SW.................................. 5  Stranger........................................ 6  Goon....... ……............................. 7  Family member (not IP)………..... 8  Friend………….…………………...9  Someone in community………... 10  Other (specify)______________97  No answer………………………..…99 |
|  | Has someone other than an intimate partner ever….Forced you to do something sexual that you found degrading or humiliating  **Kuna wakati mtu (isipokuwa mpenzi wa karibu) ashawai...**  **kulazimisha kufanya kitu ulicho hisi kilikudhalilisha wakati wa kufanya ngono.**  No………………..0 **Skip to Q832**  Yes………………1  No answer… …99 **Skip to Q832** | **Q831a**  In the past 6 months, how many times has this happened?  **Kwa miezi sita iliyopita, jambo hili limefanyika mara ngapi?**  Never…………0 **Skip to Q832**  Once…………….1  Few Times………2  Many Times…….3  No answer…….99 **Skip to Q832** | **Q831b**  Who did this to you (past 6 months)?  **Nani alifanya hivi? (miezi 6 iliyopita)**  **(record all mentioned)**  Police............................................ 1  City askari…………………………. 2  Client…......................................... 3  Pimp/Madam................................. 4  Another SW.................................. 5  Stranger........................................ 6  Goon....... ……............................. 7  Family member (not IP)………..... 8  Friend………….…………………...9  Someone in community………... 10  Other (specify)______________97  No answer………………………..…99 |
|  | Thinking about the violence you have experienced from someone who is not an intimate partner, did your children under 18 years ever witness any of this violence against you?  **Ukifikiria kuhusu aina yoyote ya vita umepitia kutoka kwa mtu asiye mpenzi wako haswa kwa miezi sita iliyopita, kuna wakati watoto wako walio chini ya miaka 18 walikuweko na kushuhudia?** | | Yes………………………………..... 1  No………………………… 2  Not applicable (no violence / children)… 3  **IF NO / NA SKIP TO Q834**  Don’t know……………………………….98  No answer……………………………….99 |

|  | Did your children under 18 years witness any of this violence against you by someone who is not an intimate partner in the past 6 months?  **Ukifikiria kuhusu aina yoyote ya vita umepitia kutoka kwa mtu asiye mpenzi wako haswa kwa miezi sita iliyopita, kuna wakati watoto wako walio chini ya miaka 18 walikuweko na kushuhudia?** | Yes………………………………..... 1  No………………………… 2  Don’t know……………………………….98  No answer……………………………….99 |
| --- | --- | --- |
|  | In the last six months how many times has someone other than an intimate partner drugged you?  **Kwa miezi sita iliyopita, kuna mtu kando ya mpenzi wako maalum amekuwekea dawa/mchele?**  ***Instruction: Put something in your drink or food which has meant you do not remember or know what happened to you*** | Never.. .................................... 0  Once........................................ 1  Few times................................ 2  Many times………................... 3  Don’t know / don’t remember. 98  No answer.............................. 99 |
|  | In the last six months how many times has someone other than your intimate partner imprisoned you?  **Kwa muda wa miezi sita iliyopita, ni mara ngapi mtu asiye mpenzi wako maalum amekufungia kinyume cha hiari yako?**  ***Instruction: Locked you up somewhere against your will?*** | Never.. .................................... 0  Once........................................ 1  Few times................................ 2  Many times………................... 3  Don’t know / don’t remember. 98  No answer.............................. 99 |
|  | In the last six months how many times has a client or someone refused to pay you after sex (even though they had agreed to pay)?  **Kwa miezi sita iliyopita, ni mara ngapi mteja ama mtu yeyote alikataa kukulipa hata baada ya maelewano?**  ***Instruction: This question refers to economic violence.*** | Never.. .................................... 0  Once........................................ 1  Few times................................ 2  Many times………................... 3  Don’t know / don’t remember. 98  No answer.............................. 99 |
|  | Have you ever been gang raped?  **Ushawai najisiwa na kikundi cha watu?**  ***Instruction: More than one person forced you to have sex with them at the same time?*** | Yes…………………………1  No…………………………..2 **Skip to Q840**  Don’t know……………….98 **Skip to Q840**  No answer………….……99 **Skip to Q840** |
|  | How many times have you been gang raped in the past 6 months?  **Kwa miezi sita iliyopita, watu zaidi ya mmoja wamekulazimisha kufanya ngono na wao kwa pamoja?** | Never.. .................................... 0  Once........................................ 1  Few times................................ 2  Many times………................... 3  Don’t know / don’t remember. 98  No answer.............................. 99 |
|  | The last time you were gang raped, how many men physically forced you to have sex with them?  **Mara ya mwisho ulibakwa na kundi, ni wanaume wangapi walikulazimisha kufanya ngono na wao?** | Number of men |____|____|  Don’t know / don’t remember. 98  No answer.............................. 99 |
|  | Has there been a time in the past 7 days when you were physically forced to have sex with someone (other than your intimate partner) even when you did not want to?  **Kwa siku saba zilizopita, kuna mtu asiye mpenzi wako maalum alikulazimisha kufanya ngono na yeye?** | No............................................ 0  Yes.......................................... 1  Don’t know / don’t remember. 98  No answer.............................. 99 |

| **ARREST AND IMPRISONMENT** | | |
| --- | --- | --- |
|  | Have you ever been arrested because you are a sex worker?  **Umewahi kamatwa sababu wewe ni muuzaji ngono?** | Yes……………… 1  No…………………………………………..2 **SKIP TO 846**  No answer………………. 99 **SKIP TO 846** |
|  | Have you been arrested in the past 6 months?  **Umekamatwa kwa miezi sita iliyopita?** | Yes……………… 1  No…………………………………… 2  No answer………………. 99 |
|  | What was the main reason for the last incidence of arrest?  **Sababu kuu iliyofanya ukamatwe ilikuwa ipi?**  **CROSS all that are mentioned** | Soliciting/cruising………… 1  Carrying condom …………………… 2  Possession of drugs……………… 3  Stealing……………………………… 4  Loitering………………………………… 5  Being drunk and disorderly………… 6  Asking for money……………………… 7  Raid…………………………………… 8  Police swoop (Msako)…………………… 9  Others (Specify)____________________97  No answer……………………………… 99 |
|  | During your last arrest, did you have sex with or perform any sexual acts on any law enforcement officers  **Wakati wa mwisho ulipokamatwa, ulifanya ngono ya aina yoyote na wale askari?** | Yes……………… 1  No…………………………………… 2  No answer………………. 99 |
|  | What did you do when you were arrested the last time?  ,  **Mara ya mwisho ulipokamatwa, ulifanya nini?**  **CROSS all that are mentioned** | Sought support of NGO………… .1  Sought support of CBO……………… .2  Sought support of Network………… .3  Sought support from other sex workers .4  Sought support from sex worker group….5  Sought legal support…………………….…6  Sought support family/regular partner… .7  I paid and got released……………………8  I bribed and got released…………………9  Sought support of AIDS tribunal…… 10  Others(Spec.)_____________________97  No Answer……………………………… 99 |
|  | Have you ever avoided police arrest by having sex or performing sexual acts on any law enforcement officers  **Umewahi epuka kukamatwa kwa kufanya ngono na askari ama walinda usalama?** | Yes……………… 1  No…………………………………… 2 **SKIP TO 848**  No answer………………. 99 **SKIP TO 848** |
|  | Has this happened in the past 6 months?  **Hili limefanyika kwa miezi sita iliyopita?** | Yes……………… 1  No…………………………………… 2  No answer………………. 99 |
|  | Have you ever been placed on remand or imprisoned because you are a sex worker?  **Umewahi fungwa jela sababu wewe ni muuza ngono?** | Yes……………………….... 1  No ………………………………………. 2 **SKIP TO 854**  No answer………………. 99 **SKIP TO 854** |
|  | In the past 6 months, have you been placed on remand or imprisoned?  **Kwa miezi sita iliyopita, umefungwa jela ama kuwekwa rumande?** | Yes……………………….... 1  No …………………………………. 2  No answer………………. 99 |
|  | What triggered the last remand or imprisonment?  **Nini kilifanya ufungwe jela mara ya mwisho?**  **CROSS all that are mentioned** | Soliciting/cruising………… 1  Carrying condom …………………… 2  Possession of drugs……………… 3  Stealing……………………………… 4  Loitering………………………………… 5  Being drunk and disorderly………… 6  Asking for money……………………… 7  Raid…………………………………… 8  Police swoop (Msako)…………………… 9  Others (Specify)___________________97  No answer……………………………… 99 |
|  | What did you do when you were last placed on remand or imprisoned?  **Mara ya mwisho ulipokamatwa, ulifanya nini?**  **CROSS all that are mentioned** | Sought support of NGO………… 1  Sought support of CBO……………… 2  Sought support of Network………… 3  Sought support from other sex workers 4  Sought support from sex worker group…5  Sought legal support………………………6  Sought support family/regular partner… 7  I bribed and got released……………… 8  Sought support of AIDS tribunal…… 9  Others(Spec.)_____________________97  No Answer……………………………… 99 |
|  | During your last remand or imprisonment did you perform any sexual acts on any law enforcement officers?  **Mara ya mwisho ulipofungwa jela, ulilazimishwa kufanya ngono na walinda jela wowote?** | Yes……………………….... 1  No …………………………………. 2  No answer………………. 99 |
|  | When you were last placed on remand or imprisoned, who looked after your children?  **Mara ya mwisho ulipofungwa jela, nani alikuwa anawachunga watoto wako?**  **CROSS all that are mentioned** | No-one………… 1  Other sex workers …………………… 2  Partner…………………… 3  Family……………… 4  NGO/Shelter……………………………… 5  Don’t have children 6  Other__________________________… 97  Don’t know……………… 98  No answer………………. 99 |
|  | Has there been a time in the past 6 months when the police have protected you from any kind of violence?  **Kwa miezi sita iliyopita, kuna wakati polisi walikulinda kutokana na vita?**  ***Instruction: For example protected you from client or mob violence*** | Never.. .............................................. 0  Once.................................................. 1  Few times....... .. ............................... 2  Many times………………................... 3  No answer……………………………..99 |
|  | Has there been a time in the past 6 months when the city askaris have protected you from any kind of violence?  **Kwa miezi sita iliyopita, kuna wakati askari wa jiji (Kanjo) walikulinda kutokana na vita?** | Never.. .............................................. 0  Once.................................................. 1  Few times....... .. ................................ 2  Many times………………................... 3  No answer……………………………..99 |
|  | Has there been a time in the past 6 months when a client has protected you from any kind of violence?  **Kwa miezi sita iliyopita, kuna wakati mteja alikulinda kutokana na vita?** | Never.. .............................................. 0  Once.................................................. 1  Few times....... .. ................................ 2  Many times………………................... 3  No answer……………………………..99 |
|  | Has there been a time in the past 6 months when someone else has protected you from any kind of violence?  **Kwa miezi sita iliyopita, kuna wakati mtu mwengine alikulinda kutokana na vita?** | Never.. .............................................. 0 **SKIP TO Q901**  Once.................................................. 1  Few times....... .. ................................ 2  Many times………………................... 3  No answer……………………………..99 |

|  | Who was this?  **Huyu mtu alikuwa nani?**  **Cross any that are mentioned** | Main partner/lover 1  Other Female Sex Worker 2  Employee at a bar (including bouncers) 3  Member of the public/stranger 4  Other____________________________97  No answer………………. 99 | |
| --- | --- | --- | --- |
| **SECTION 9. ADVERSE CHILDHOOD EXPERIENCES**  **WHO ACE-1Q** | | | |
| This next section asks you about your life when you were a child. For our study to work we need participants to answer questions honestly. I’d like to reassure you again that all your answers will be kept strictly confidential.  **Katika sehemu inayofuata, nitakuuliza kuhusu maisha yako ya utotoni. Ili ushiriki ufanyike tulivyokusudia, tutahitaji washiriki wajibu maswali kwa njia ya kweli. Nakuhakikishia kwamba majibu yako yatahifadhiwa kwa usiri mkuu.**  While you were growing up, during your first 18 years of life… **Ulipokuwa unakua, kwa miaka 18 ya kwanza ya maisha yako…** | | | |
|  | Did you live with a household member who was a problem drinker or alcoholic, or misused street or prescription drugs?  **Uliwahi ishi na mmoja wa jamii yako aliyekuwa mlevi sana ama alikuwa anatumia vibaya dawa zozote zile?**  ***The term "problem drinker" is used here to indicate a household member whose drinking of alcohol affected their functioning (employment, household tasks, family relationships) but who was not given the label of "alcoholic". The "misuse" of drugs refers to the taking of street or prescription drugs for non-therapeutic purposes.*** | | No…………………………………………0  Yes………………………………………1  No parent/guardian……………… ….…...97  Decline to answer……………………….99 |
|  | During the first 18 years of your life . . Did you live with a household member who was depressed, mentally ill or suicidal?  **Ulipokuwa unakua, kwa miaka 18 ya kwanza ya maisha yako,**  **Uliwahi ishi na mmoja wa jamii yako aliyekuwa na shida ya akili ama alitaka kujiua?** | | No…………………………………………0  Yes………………………………………1  No parent/guardian……………… ….…...97  Decline to answer……………………….99 |
|  | During the first 18 years of your life . . Did you live with a household member who was ever sent to jail or prison?  **Ulipokuwa unakua, kwa miaka 18 ya kwanza ya maisha yako Uliwahi ishi na mmoja wa jamii yako aliyefungwa jela?** | | No…………………………………………0  Yes………………………………………1  No parent/guardian……………… ….…...97  Decline to answer……………………….99 |
|  | During the first 18 years of your life . . Were your parents ever separated or divorced?  **Ulipokuwa unakua, kwa miaka 18 ya kwanza ya maisha yako Wazazi wako walitalikiana?**  ***This question refers to whether the participant's parents were separated or divorced while they were growing up. If the participant's parents were never married then cross "not applicable”.*** | | No……………………………………………..0  Yes……………………………………………1  No parent/guardian……………… ….…...97  Not Applicable……………..……………..98  Decline to answer……………………….99 |
|  | During the first 18 years of your life . . Did your mother, father or guardian die?  **Ulipokuwa unakua, kwa miaka 18 ya kwanza ya maisha yako Mamako, babako ama mlezi wako alifariki?**  ***If the participant was raised in care and does not know whether their parents died then cross "Don't know/Not sure".*** | | No……………………………………………..0  Yes……………………………………………1  Don’t know / Not sure…..………… ….…98  Decline to answer……………………….99 |
|  | During the first 18 years of your life . ., did you ever live on the streets?  **Ulipokuwa unakua, kwa miaka 18 ya kwanza ya maisha yako Kuna wakati wowote uliishi mitaani?** | | No……………………………………………..0  Yes……………………………………………1    Don’t know / Not sure…..………… ….…98  Decline to answer……………………….99 |
| **These next questions are about certain things you may actually have heard or seen IN YOUR HOME. These are things that may have been done to another household member but not necessarily to you.**  **Maswali yafuatayo ni kuhusu vitu ambavyo pengine uliona ama uliskia zikifanyika nyumbani kwenu. Haya ni mambo ambayo yanaweza kuwa yalifanyika lakini hayakukufanyikia wewe mwenyewe.**  When you were growing up, during the first 18 years of your life… **Wakati ulipokuwa mdogo, kabla ufikishe miaka 18……** | | | |
|  | Did you see or hear a parent or household member in your home being yelled at, screamed at, sworn at, insulted or humiliated?  **Uliwahi ona au kusikia mzazi ama mmoja wa jamii mnaoishi na wao wakikorofishana, kudhalilishwa, kutusiwa ama kudharauliwa?**  ***This question is asking about verbal abuse only.*** | | Many times………………………...……1  A few times……………………….……..2 Once…..…………………………..……..3  Never……………………………….……4  No parent/guardian……………… …….97  Decline to answer…………………….99 |
|  | During the first 18 years of your life . . Did you see or hear a parent or household member in your home being slapped, kicked, punched or beaten up?  **Ulipokuwa unakua, kwa miaka 18 ya kwanza ya maisha yako…..Uliwahi ona au kusikia mzazi ama mmoja wa jamii mnaoishi na wao akipigwa kofi, kuchapwa mateke, ngumi ama kupigwa?**  ***This question is asking about physical abuse without the use of a weapon or implement.*** | | Many times………………………...……1  A few times……………………….……..2 Once…..…………………………..……..3  Never……………………………….……4  No parent/guardian……………… …….97  Decline to answer…………………….99 |
|  | During the first 18 years of your life . . Did you see or hear a parent or household member in your home being hit or cut with an object, such as a stick (or cane), bottle, club, knife, whip etc.?  **Ulipokuwa unakua, kwa miaka 18 ya kwanza ya maisha yako…..Uliwahi ona au kusikia mzazi ama mmoja wa jamii mnaoishi na wao akichapwa au kukatwa na kifaa chochote kile kama vile kiboko, chupa, kisu n.k.?**  ***This question is asking about physical abuse with the use of a weapon or implement.*** | | Many times………………………...……1  A few times……………………….……..2 Once…..…………………………..……..3  Never……………………………….……4  No parent/guardian……………… …….97  Decline to answer…………………….99 |
| **These next questions are about certain things YOU may have experienced. When you were growing up, during the first 18 years of your life . . .**  **Maswali yafuatayo ni kuhhusu vitu ambavyo pengine ulipitia. Wakati ulipokuwa mdogo, kabla ufikishe miaka 18……** | | | |
|  | During the first 18 years of your life . . Did a parent, guardian or other household member yell, scream or swear at you, insult or humiliate you?  **Ulipokuwa unakua, kwa miaka 18 ya kwanza ya maisha yako…..Kuna mzazi, mlezi ama mmoja wa jamii yako aliwahi kukukelelesha, kutusi ama kukudhalilisha?**  ***This question is asking about verbal abuse only.*** | | Many times………………………...……1  A few times……………………….……..2 Once…..…………………………..……..3  Never……………………………….……4  No parent/guardian……………… …….97  Decline to answer…………………….99 |

|  | During the first 18 years of your life . . Did a parent, guardian or other household member threaten to, or actually, abandon you or throw you out of the house?  **Ulipokuwa unakua, kwa miaka 18 ya kwanza ya maisha yako…..Kuna mzazi, mlezi ama mmoja wa jamii yako aliwahi kutishia ama kukutupa nje ya nyumba?**  ***This question is asking both about verbal threats of abandonment, and actual acts of abandonment, made to the participant.*** | Many times………………………...……1  A few times……………………….……..2 Once…..…………………………..……..3  Never……………………………….……4  No parent/guardian……………… …….97  Decline to answer…………………….99 |
| --- | --- | --- |
|  | During the first 18 years of your life . . Did a parent, guardian or other household member spank, slap, kick, punch or beat you up?  **Ulipokuwa unakua, kwa miaka 18 ya kwanza ya maisha yako…..Kuna mzazi, mlezi ama mmoja wa jamii yako aliwahi kuchapa, kupiga makofi, ngumi ama mateke au kukutandika?**  ***This question is asking about physical abuse without the use of a weapon or implement.*** | Many times………………………...……1  A few times……………………….……..2 Once…..…………………………..……..3  Never……………………………….……4  No parent/guardian……………… …….97  Decline to answer…………………….99 |
|  | During the first 18 years of your life . . Did a parent, guardian or other household member hit or cut you with an object, such as a stick (or cane), bottle, club, knife, whip etc?  **Ulipokuwa unakua, kwa miaka 18 ya kwanza ya maisha yako…..Kuna mzazi, mlezi ama mmoja wa jamii yako aliwahi kukuchapa na kifaa cha aina yoyote kama fimbo, rungu, chupa kisu,mjeledi n,k?**  ***This question is asking about physical abuse with the use of a weapon or implement.*** | Many times………………………...……1  A few times……………………….……..2 Once…..…………………………..……..3  Never……………………………….……4  No parent/guardian……………… …….97  Decline to answer…………………….99 |
|  | During the first 18 years of your life . . Did someone touch or fondle you in a sexual way when you did not want them to?  **Ulipokuwa unakua, kwa miaka 18 ya kwanza ya maisha yako…..kuna mtu alikuguza kwa njia ya kuashiria ngono ilhali haukuwa unataka?**  ***Instruction: These next questions are not just about a family or household member - it could have been anyone, known or unknown, to the participant.*** | Many times………………………...……1  A few times……………………….……..2 Once…..…………………………..……..3  Never……………………………….……4  Decline to answer…………………….99 |
|  | During the first 18 years of your life . . Did someone make you touch their body in a sexual way when you did not want them to?  **Ulipokuwa unakua, kwa miaka 18 ya kwanza ya maisha yako…..Kuna mtu yeyote aliwahi kulazimisha kumguza kwa njia ya kuashiria ngono ilhali haukutaka?** | Many times………………………...……1  A few times……………………….……..2 Once…..…………………………..……..3  Never……………………………….……4  Decline to answer…………………….99 |
|  | During the first 18 years of your life . . Did someone attempt oral, anal, or vaginal intercourse with you when you did not want them to?  **Ulipokuwa unakua, kwa miaka 18 ya kwanza ya maisha yako…..Kuna mtu yeyote aliwahi jaribu ngono ya aina yoyote na wewe ihali haukutaka?** | Many times………………………...……1  A few times……………………….……..2 Once…..…………………………..……..3  Never……………………………….……4  Decline to answer…………………….99 |
|  | During the first 18 years of your life . . Did someone actually have oral, anal, or vaginal intercourse with you when you did not want them to?  **Ulipokuwa unakua, kwa miaka 18 ya kwanza ya maisha yako…..Kuna mtu yeyote aliwahi fanya ngono ya aina yoyote na wewe ihali haukutaka?** | Many times………………………...……1  A few times……………………….……..2 Once…..…………………………..……..3  Never……………………………….……4  Decline to answer…………………….99 |
| **WITNESSING COMMUNITY VIOLENCE**  These next questions are about how often, when you were a child, YOU may have seen or heard certain things in your NEIGHBOURHOOD OR COMMUNITY (not in your home or on TV, movies, or the radio).  **Maswali haya yafuatayo ni kuhusu mara ngapi, wakati ulipokuwa mtoto, huenda umeona au kusikia mambo fulani katika JIRANI WAKO AMA JUMUIA (si nyumbani kwako au kwenye televisheni, sinema, au redio).**  **When you were growing up, during the first 18 years of your life . . .**  **Ulipokua unakua**, **kwa miaka 18 ya kwanza ya maisha yako**. . . | | |
|  | Did you see or hear someone being beaten up in real life?  **Uliwahi ona au kuskia mtu akichapwa moja kwa moja?**  *These questions refers to things that the participant actually witnessed for themselves - it is not asking if these things happened in their neighbourhood or community in general, or whether the participant saw or heard them on TV, movies or the radio.* | Many times………………………...……1  A few times……………………….……..2 Once…..…………………………..……..3  Never……………………………….……4  Decline to answer…………………….99 |
|  | During the first 18 years of your life . . Did you see or hear someone being stabbed or shot in real life?  **Umewahi shuhudia mtu akidungwa kisu ama kupigwa risasi?** | Many times………………………...……1  A few times……………………….……..2 Once…..…………………………..……..3  Never……………………………….……4  Decline to answer…………………….99 |
|  | During the first 18 years of your life . . Did you see or hear someone being threatened with a knife or gun in real life?  **Umewahi ona ama kusikia mtu akitishiwa na kisu ama bunduki?** | Many times………………………...……1  A few times……………………….……..2 Once…..…………………………..……..3  Never……………………………….……4  Decline to answer…………………….99 |
| **EXPOSURE TO WAR/COLLECTIVE VIOLENCE**  These questions are about whether YOU did or did not experience any of the following events when you were a child. The events are all to do with collective violence, including wars, terrorism, political or ethnic conflicts, genocide, repression, disappearances, torture and organized violent crime such as banditry and gang warfare.  **Maswali haya ni juu ya kama ulifanya au haujapata matukio yoyote yafuatayo wakati ulipokuwa mtoto. Matukio haya yote yanahusiana na vurugu, pamoja na vita, ugaidi, migogoro ya kisiasa au kikabila, mauaji ya kimbari, ukandamizaji, kutoweka, mateso na uhalifu wa uhalifu kama vile bandari na vita vya genge.** | | |
|  | During the first 18 years of your life . . Were you forced to go and live in another place due to any of these events?  **Ulipokuwa unakua, kwa miaka 18 ya kwanza ya maisha yako…..Umewahi lazimishwa kuhama juu ya matukio kama haya?**  ***This question is only referring to whether any of the events above resulted in the participant being forced to leave their home and live elsewhere (this is not about the participant and their family voluntarily fleeing from a danger zone).*** | Many times………………………...……1  A few times……………………….……..2 Once…..…………………………..……..3  Never……………………………….……4  Decline to answer…………………….99 |
|  | During the first 18 years of your life . . Did you experience the deliberate destruction of your home due to any of these events?  **Ulipokuwa unakua, kwa miaka 18 ya kwanza ya maisha yako…..Makao yako yamewahi haribiwa kimakusudi na matukio haya?**  ***This question is only referring to whether any of the events above resulted in the deliberate destruction of the participant's home.*** | Many times………………………...……1  A few times……………………….……..2 Once…..…………………………..……..3  Never……………………………….……4  Decline to answer…………………….99 |

|  | During the first 18 years of your life . . Were you beaten up by soldiers, police, militia, or gangs?  **Ulipokuwa unakua, kwa miaka 18 ya kwanza ya maisha yako…..Uliwahi chapwa na polisi ama militia ama vikundi haramu vyovyote?** | Many times………………………...……1  A few times……………………….……..2 Once…..…………………………..……..3  Never……………………………….……4  Decline to answer…………………….99 |
| --- | --- | --- |
|  | During the first 18 years of your life . . Was a family member or friend killed or beaten up by soldiers, police, militia, or gangs?  **Ulipokuwa unakua, kwa miaka 18 ya kwanza ya maisha yako…..Kuna mmoja wa jamii yako ama rafiki aliyeuliwa ama kuchapwa na walinda usalama ama vikundi vyovyote haramu?** | Many times………………………...……1  A few times……………………….……..2 Once…..…………………………..……..3  Never……………………………….……4  Decline to answer…………………….99 |

| SECTION 10. STI/HIV TESTING AND TREATMENT | | |
| --- | --- | --- |
| This next section is going to ask you about your knowledge and experiences around HIV and STI testing and treatment. For our study to work we need participants to answer questions honestly. I’d like to reassure you again that all your answers will be kept strictly confidential. **Sehemu inayofuata itakuuliza juu ya ujuzi na uzoefu wako kuhusu kupima VVU na magonjwa ya zinaa na matibabu. Kwa ajili ya utafiti wetu kufanya kazi tunahitaji washiriki kujibu maswali kwa uaminifu. Napenda kukuhakikishia tena kwamba majibu yako yote yatahifadhiwa kwa siri** | | |
|  | In the past 30 days, have you taken antibiotics to treat a sexually transmitted or reproductive tract infection?  **Kwa siku 30 zilizopita, umekunywa dawa za kutibu ugonjwa wa zinaa?** | Yes……………………………………1  No……………………………………..2 **Skip to Q1003**  Decline to answer………………….99 **Skip to Q1003** |
|  | If yes, did you get these from a SWOP clinic?  **Kama ndio, ulipokea hizi dawa kutoka kwa kliniki za SWOP?** | Yes…………………………………………………1  No…………………………………………………..2  Decline to answer……………………………….99 |
|  | Have you ever taken an HIV test?  **Umewahi pimwa VVU?** | Yes……………………….. 1  No………………………… 2  Don’t know……………… 98  No answer……………… 99 |
|  | When was the last time you had an HIV test?  **Mara ya mwisho ulipopimwa VVU ni lini?** | < 3 months ago……………………… 1  3 to <6 months ago……………………… 2  6 to <12 months ago……………… 3  12 or more months ago……………… 4  No answer……………………………… 99 |
|  | What is your HIV status?  **Hali yako ya VVU ni gani?**  ***Instruction: For people who answer don’t’ know, probe what was the result of your last HIV test*** | Positive……………………….. 1  Negative………………………………2 **SKIP TO 1018**  Don’t know……………………………98 **SKIP TO 1018**  No answer……………………………99 **SKIP TO 1018** |
|  | How old were you when you had your first positive HIV test?  **Ulikuwa na miaka mingapi ulipopokea matokeo kuwa una VVU?** | Years  Born Positive…………………..…………..97  Don’t know……………………………….. 98  No answer…………………………………99 |
|  | Have you ever been prescribed ARVs?  **Umewahi andikiwa dawa za kupumguza makali ya VVU na daktari?** | Yes………………………………. 1  No………………………………… 2  Don’t know……………………………….. 98  No answer…………………………………99 |

|  | Have you ever taken ARVs?  **Umewahi meza dawa za kupunguza makali ya VVU?** | | | Yes………………………………….….1  No………………………………………2  **SKIP TO 1013**  Don’t know……………………………98 **SKIP TO 1013**  No answer……………………………99 **SKIP TO 1013** | | | |
| --- | --- | --- | --- | --- | --- | --- | --- |
|  | How old were you the first time you ever took ARVs?  **Ulikuwa na umri wa miaka mingapi ulipomeza dawa za kupunguza makali ya VVU mara ya kwanza?** | | | Years  Since birth……………………………..…..96  Since childhood (<10 years)………..……97  Don’t know……………………………….. 98  No answer…………………………………99  ***Compare with response in section 1006 above*** | | | |
|  | Since that first time, has there been a period of time of more than one month when you stopped taking ARVs?  **Tangu huo wakati, umewahi kaa zaidi ya mwezi mmoja ambapo uliasi kutumia dawa hizo?** | | | Yes………………………………. 1  No………………………………… 2  Don’t know……………………………….. 98  No answer…………………………………99 | | | |
|  | Are you currently taking ARVs?  **Kwa wakati huu, unakunywa dawa za kupunguza makali ya VVU?** | | | Yes……………………………..………….1  **SKIP TO 1014** No………………………………… 2  Don’t know……………………………….. 98  No answer…………………………………99 | | | |
|  | How long ago did you last take ARVs?  **Mara ya mwsho ulipokunywa dawa za kupunguza makali ya VVU ni lini?** | | | Months…………………..  Years…………………….  No answer……………. 99 | | | |
|  | If you are not currently taking ARV medicine, what are the main reasons for not taking ARVs?  **Kama umeasi kunywa dawa, sababu kuu ni ipi?**  **Multiple responses apply. CROSS all.** | | | Side effects of medication…………..… … 1  Scared someone will find pills ……………… 2  Ran out of pills……………….………… ….. 3  Packaging of pills……………………………… 4  Did not like the ARV facility staff…………….. 5  Unable to attend ARV facility ………………. 6  Want to die…………..……………………….. 7  Prayers/beliefs………………………………. 8  Other__________________________________ 97  No answer………………………………… 99  **SKIP TO 1101** | | | |
|  | Do you currently get your ARVs from a SWOP clinic?  **Unapata dawa za kupunguza makali ya VVU kliniki ya SWOP?** | | | No………………………………… 0  Yes………………………………. 1  Don’t know…………… 98  No answer……………. 99 | | | |
|  | Was there an occasion in the last 1 month where you missed taking any of your ARV pills?  **Kwa mwezi uliopita, kuna wakati uliasi kunywa dawa zako za kupunguza makalli ya VVU?** | | | No………………………………… 0  Yes………………………………. 1  Don’t know…………… 98  No answer……………. 99 | | | |
|  | Was there an occasion in the last seven days where you missed taking any of your ARV pills?  **Kwa siku saba zilizopita, kuna wakati uliasi kunywa dawa zako za kupunguza makalli ya VVU?** | | | No………………………………… 0  Yes………………………………. 1  Don’t know…………… 98  No answer……………. 99 | | | |
|  | What was the **main** reason you missed taking any of your ARV pills in the past 7 days?  **Kama umeasi kunywa dawa kwa siku saba zilizopita, sababu kuu ni ipi?** | | | Side effects of medication…………..… … 1  Scared someone will find pills ……………… 2  Ran out of pills……………….………… ….. 3  Packaging of pills……………………………… 4  Did not like the ARV facility staff…………….. 5  Unable to attend ARV facility ………………. 6  Want to die…………..……………………….. 7  Prayers/beliefs………………………………. 8  Other__________________________________ 97  No answer………………………………… 99  **SKIP TO 1101** | | | |
| ***Ask these questions for women who report last HIV test was negative:*** | | | | | | | |
|  | Have you ever heard of PrEP (Pre-Exposure Prohpylaxis)?  **Umewahi sikia kuhusu dawa ya kukinga kutokana VVU ya PrEP?** | | | No…………………………….................0 **SKIP TO 1025** Yes………………………………………1  Don’t know……………………………….98  No answer……………………………….99 | | | |
|  | Have you ever taken PrEP?  **Umewahi kunywa PrEP?** | | | No……………………………....................0 **SKIP TO 1021**  Yes………………………………. 1  Unsure………………………………..……2  No answer………………………. 99 | | | |
|  | Are you currently taking PrEP?  **Kwa wakati huu unakunywa PrEP?** | | | No………………………………… 0  Yes………………………………………...1 **SKIP TO 1022** Don’t know……………………………….. 98  No answer…………………………………99 | | | |
|  | If you are not currently taking PREP what are the main reasons for not taking PREP?  **Kama hautumii PrEP, ni sababu gani zinakufanya usiitumie?**  **DO NOT READ OPTIONS. Multiple responses apply. CROSS all mentioned.** | | | Side effects of medication…………..… … 1  Scared someone will find pills ……………… 2  Ran out of pills……………….………… ….. 3  Packaging of pills……………………………… 4  Did not like the ARV facility staff…………….. 5  Unable to attend ARV facility ………………. 6  Want to die…………..……………………….. 7  Prayers/beliefs………………………………. 8  Do not feel at risk of HIV…………………………..9  Other__________________________________ 97  No answer………………………………… 99  **SKIP TO 1025** | | | |
|  | Was there an occasion in the last 1 month where you missed taking any of you’re PREP pills?  **Kuna wakati kwa mwezi mmoja uliopita ulikosa kunywa PrEP?** | | | No………………………………… 0  Yes………………………………. 1  Don’t know……………………………….. 98  No answer…………………………………99 | | | |
|  | Was there an occasion in the last 7 days where you missed taking any of your PREP pills?  **Kuna wakati kwa wiki moja iliopita ulikosa kunywa PrEP?** | | | No…………………… 0 **SKIP TO 1025**  Yes………………………………. 1  Don’t know………………………………98  No answer………………………………99 | | | |
|  | What was the **main** reason you missed taking any of your PREP pills in the past 7 days?  **Sababu gani kuu ilikufanya kuasi kunywa PrEP kwa siku saba zilizopita** | | | Side effects of medication…………..… … 1  Scared someone will find pills ……………… 2  Ran out of pills……………….………… ….. 3  Packaging of pills……………………………… 4  Did not like the ARV facility staff…………….. 5  Unable to attend ARV facility ………………. 6  Want to die…………..……………………….. 7  Prayers/beliefs………………………………. 8  Do not feel at risk of HIV…………………………..9  Other__________________________________ 10  No answer………………………………… 99 | | | |
|  | Have you ever heard of PEP (Post-exposure prophylaxis)  **Umewahi sikia kuhusu PEP?** | | | No…………………………………………0 **SKIP TO 1101**  Yes………………………………………..1  Don’t know……………………………….. 98  No answer…………………………………99 | | | |
|  | Have you ever taken PEP?  **Umewahi tumia PEP?** | | | No…………………………………………0 **SKIP TO 1101**  Yes………………………………………..1  Don’t know……………………………….. 98  No answer…………………………………99 | | | |
|  | What was the reason for taking PEP the last time you took it?  **Kwa nini ulitumia PEP mara ya mwisho ulipoitumia?**  **DO NOT READ OPTIONS. Multiple responses apply. CROSS all mentioned.** | | | Condom burst/slippage/breakage…… 1  Rape/ Sexual assault……………… 2  Unprotected sex……………………… 3  Sharing needles………………………… 4  Other specify____________________ 97 | | | |
|  | The last time you took PEP, did you miss taking any of your pills?  **Mara ya mwisho ulipotumia PEP, uliasi kunywa tembe zozote?** | | | No………………………..……………….…0  Yes………………………………..…….….1  Currently on PEP………. 3  No answer……………… 99 | | | |
|  | Are you currently taking PEP?  **Kwa wakati huu unatumia PEP?** | | | No………………………..………………0 **SKIP TO 1101**  Yes……………………………………….1  No answer……………… 99 | | | |
|  | In the last 7 days, did you miss taking any of your PEP pills?  **Kwa siku 7 zillizopita, Kuna wakati ulikosa kunywa PEP zako?**  ***INSTRUCTION: IF STARTED PEP WITHING THE PAST 7 DAYS, ASK IF MISSED ANY PILLS SINCE STARTING THIS PEP MEDICATION*** | | | No…………………………………………..0 **SKIP TO 1101**  Yes…………………………………………1  No answer……………… 99 | | | |
|  | What was the main reason you missed taking any of your PEP pills in the past 7 days?  **Sababu kuu ya kutotumia PEP kwa siku 7 zilizopita ni ipi?** | | | Side effects of medication…………..… … 1  Scared someone will find pills ……………… 2  Ran out of pills……………….………… ….. 3  Packaging of pills……………………………… 4  Did not like the ARV facility staff…………….. 5  Unable to attend ARV facility ………………. 6  Want to die…………..……………………….. 7  Prayers/beliefs………………………………. 8  Do not feel at risk of HIV…………………………..9  Other__________________________________ 97  No answer………………………………… 99 | | | |
| **SECTION 11. STIGMA AROUND SEX WORK AND HIV** | | | | | | | |
| The next few questions are about shame, stigma, harassment, or discrimination you may have experienced.  Would you say the following has happened to you, either ever or in the last 12 months?  **Maswali machache ijayo ni juu ya aibu, unyanyapaa, unyanyasaji, au ubaguzi ambao unaweza kuwa na uzoefu. Je! Unasema zifuatazo zimekutokea, ama milele au miezi 12 iliyopita?** | | | | | | | |
|  | I have lost respect or standing in the community because I sell sex  **Nimepoteza heshima au kupoteza sauti katika jamii kwa sababu ninauza ngono** | | | | No………………………………… 1  No because no-one knows I sell sex……2  Yes, in the last 12 months ……………… 3  Yes, but not in the last 12 months …… 4  Don’t know……………………………….. 98  No answer…………………………………99 | | |
|  | I think less of myself because I sell sex  **Najidharau kwa sababu ninauza ngono** | | | | No………………………………… 1  Yes, in the last 12 months ……………… 2  Yes, but not in the last 12 months …… 3  Don’t know……………………………….. 98  No answer…………………………………99 | | |
|  | I have felt ashamed because I sell sex  **Nimehisi aibu kwa sababu ninauza ngono** | | | | No………………………………… 1  Yes, in the last 12 months ……………… 2  Yes, but not in the last 12 months …… 3  Don’t know……………………………….. 98  No answer…………………………………99 | | |
|  | People have talked badly about me because I sell sex  **Watu wamezungumza vibaya juu yangu kwa sababu ninauza ngono** | | | | No………………………………… 1  No because no-one knows I sell sex……2  Yes, in the last 12 months ……………… 3  Yes, but not in the last 12 months …… 4  Don’t know……………………………….. 98  No answer…………………………………99 | | |
|  | I have been verbally insulted, harassed or threatened because I sell sex?  **Nimekuwa nikidhulumiwa, kusumbuliwa au kutishiwa kwa sababu ninauza ngono?** | | | | No………………………………… 1  Yes, in the last 12 months ……………… 2  Yes, but not in the last 12 months …… 3  Don’t know……………………………….. 98  No answer…………………………………99 | | |
|  | I have felt excluded from or rejected by my family because I sell sex?  **Nimehisi kama natengwa au kukataliwa na familia yangu kwa sababu ninauza ngono?** | | | | No………………………………… 1  No because no-one knows I sell sex……2  Yes, in the last 12 months ……………… 3  Yes, but not in the last 12 months …… 4  Don’t know……………………………….. 98  No answer…………………………………99 | | |
|  | I have avoided seeking health services because I am worried someone may learn I sell sex?  **Nimekosa kwenda kutafuta huduma za afya kwa sababu nina wasiwasi mtu anaweza jua kuwa ninauza ngono?** | | | | No………………………………… 1  Yes, in the last 12 months ……………… 2  Yes, but not in the last 12 months …… 3  Don’t know……………………………….. 98  No answer…………………………………99 | | |
|  | I have been denied health services or experienced an increase in cost because I sell sex?  **Nimewahi kosa kupewa huduma za afya au Nikaongezewa gharama kwa sababu ninauza ngono?** | | | | No………………………………… 1  No because no-one knows I sell sex……2  Yes, in the last 12 months ……………… 3  Yes, but not in the last 12 months …… 4  Don’t know……………………………….. 98  No answer…………………………………99 | | |
| **STIGMA AROUND HIV**  ***Q1109-1123 to be asked to HIV POSITIVE WOMEN Q1124- 1131 to be asked to HIV NEGATIVE WOMEN*** | | | | | | | |
|  | | | I have lost respect or standing in the community because of my HIV status  **Nimepoteza heshima au kupoteza sauti katika jamii kwa sababu ya hali yangu ya VVU** | | No………………………………… 1  No because no-one knows my status…..2  Yes, in the last 12 months ……………… 3  Yes, but not in the last 12 months …… 4  Don’t know……………………………….. 98  No answer…………………………………99 | | |
|  | | | I think less of myself because of my HIV status  **Najidharau kwa sababu ya hali yangu ya VVU** | | No………………………………… 1  Yes, in the last 12 months ……………… 2  Yes, but not in the last 12 months …… 3  Don’t know……………………………….. 98  No answer…………………………………99 | | |
|  | | | I have felt ashamed because of my HIV status  **Nimesikia aibu kwa sababu ya hali yangu ya VVU** | | No………………………………… 1  Yes, in the last 12 months ……………… 2  Yes, but not in the last 12 months …… 3  Don’t know……………………………….. 98  No answer…………………………………99 | | |
|  | | | People have talked badly about me because of my HIV status  **Watu wamezungumza vibaya juu yangu kwa sababu ya hali yangu ya VVU** | | No………………………………… 1  No because no-one knows my status…..2  Yes, in the last 12 months ……………… 3  Yes, but not in the last 12 months …… 4  Don’t know……………………………….. 98  No answer…………………………………99 | | |
|  | | | I have been verbally insulted, harassed or threatened because of my HIV status  **Nimekuwa nikidhulumiwa, kusumbuliwa au kutishiwa kwa sababu ya hali yangu ya VVU** | | No………………………………… 1  No because no-one knows my status…..2  Yes, in the last 12 months ……………… 3  Yes, but not in the last 12 months …… 4  Don’t know……………………………….. 98  No answer…………………………………99 | | |
|  | | | I have felt that people have not wanted to sit next to me, for example on public transport, at church or in a waiting room because of my HIV status  **Nimehisi kwamba watu hawakutaka kukaa karibu nami, kwa mfano kwa usafiri wa umma(matatu), kanisani au chumba cha kusubiri kwa sababu ya hali yangu ya VVU** | | No………………………………… 1  No because no-one knows my status…..2  Yes, in the last 12 months ……………… 3  Yes, but not in the last 12 months …… 4  Don’t know……………………………….. 98  No answer…………………………………99 | | |
| QQ | | | Someone else disclosed my HIV status without my permission  **Mtu mwingine alitangaza hali yangu ya VVU bila idhini yangu** | | No………………………………… 1  Yes, in the last 12 months ……………… 2  Yes, but not in the last 12 months …… 3  Dont know……………………………….. 98  No answer…………………………………99 | | |
| ***Q1116 – 1123 TO BE ASKED TO HIV POSITIVE WOMEN.***  ***Do you agree, neither agree or disagree, or disagree with the following statements?***  ***Unakubaliana, uko hapo katikati ama haukubaliani na maelezo yafuatayo?*** | | | | | | | |
|  | |  | | | Agree | Neither agree or disagree | Disagree |
|  | | I feel ashamed of using ARVs  **Ninahisi aibu kuhusu kutumia ARVs** | | | 1 | 2 | 3 |
|  | | I would feel embarrassed if others knew I was using ARVs  **Ninahisi kudhalalika kuhusu kutumia ARVs** | | | 1 | 2 | 3 |
|  | | I feel empowered to use ARVs  **Ninahisi kuwa na ujuzi wa kutosha kuniwezesha kutumia ARVs** | | | 1 | 2 | 3 |
|  | | I think I am not following the ‘rules' (expectations) of my community if I take ARVs to prevent HIV / illness  **Nadhani sifuati 'sheria' (matarajio) ya jumuiya yangu ikiwa natumia ARVs kuzuia VVU / ugonjwa** | | | 1 | 2 | 3 |
|  | | **I think people will give me a hard time (such as make fun of me, or talk badly about me) if I tell them I am taking ARVs**  **Nadhani watu watanipa wakati mgumu (kama kunidharau, au kuzungumza vibaya kunihusu) nikiwaambia ninatumia ARVs** | | | 1 | 2 | 3 |
|  | | I think people will judge negatively if I take ARVs  **Nadhani watu watanihukumu visivyo nikiwaambia natumia ARVs** | | | 1 | 2 | 3 |
|  | | I think I am at greater risk for physical violence or rape if I take ARVs  **Nadhani niko kwa hatari kubwa zaidi kunyanyaswa kimwili au kubakwa ikiwa natumia ARVs** | | | 1 | 2 | 3 |
|  | | People will think I am behaving responsibly by taking ARVs  **Watu watafikiri kuwa ninawajibika kwa kuchukua ARVs**  **SKIP TO Q1201** | | | 1 | 2 | 3 |
| ***Q1124-1131 TO BE ASKED TO HIV NEGATIVE WOMEN.***  ***Do you agree, neither agree or disagree, or disagree with the following statements?***  ***Unakubaliana, uko hapo katikati ama haukubaliani na maelezo yafuatayo?*** | | | | | | | |
|  | | | | | Agree | Neither agree or disagree | Disagree |
|  | I feel ashamed of using PrEP  **Nina aibu ya kutumia PrEP** | | | | 1 | 2 | 3 |
|  | I would feel embarrassed if others knew I was using PrEP  **Ninahisi kudhalilishwa watu wakijua natumia PrEP** | | | | 1 | 2 | 3 |
|  | I feel empowered to use PrEP  **Ninahisi kuwa na ujuzi wa kutosha kuniwezesha kutumia PrEP** | | | | 1 | 2 | 3 |
|  | I think I am not following the ‘rules' (expectations) of my community if I take PrEP to prevent HIV / illness  **Nadhani sifuati 'sheria' (matarajio) ya jumuiya yangu ikiwa ninachukua PrEP kuzuia VVU / ugonjwa** | | | | 1 | 2 | 3 |
|  | I think people will give me a hard time (such as make fun of me, or talk badly about me) if I tell them I am taking PrEP  **Nadhani watu watanipa wakati mgumu (kama kunidharau, au kuzungumza vibaya juu yangu) nikiwaambia ninatumia PrEP** | | | | 1 | 2 | 3 |
|  | I think people will judge negatively if I take PrEP  **Nadhani watu watanihukumu visivyo nikiwaambia natumia PrEP** | | | | 1 | 2 | 3 |
|  | I think I am at greater risk for physical violence or rape if I take PrEP  **Nadhani niko kwa hatari kubwa zaidi kunyanyaswa kimwili au kubakwa ikiwa natumia PrEP** | | | | 1 | 2 | 3 |
|  | People will think I am behaving responsibly by taking PrEP  **Watu watafikiri kuwa ninawajibika kwa kutumia PrEP** | | | | 1 | 2 | 3 |

| SECTION 12. MENTAL HEALTH | | | | | | | | | | |
| --- | --- | --- | --- | --- | --- | --- | --- | --- | --- | --- |
| **Common Mental Disorders (PHQ-9 and GAD-7 tools)**  **In this section I am going to ask you questions to understand your mental health. Please know that there are no right or wrong answers to any of these questions. And that anything you tell me will remain confidential.**  **Katika sehemu hii nitakuuliza maswali ili kuelewa afya yako ya akili. Tafadhali jua kwamba hakuna jibu sahihi au lisilo sawa kwa maswali yoyote haya. Na kwamba kitu chochote unaniambia kitabaki siri.**  Over the last two weeks, how often have you been bothered by any of the following problems?  **Kwa muda wa wiki mbili za mwisho, ni mara ngapi umesumbuliwa na matatizo yoyote yafuatayo?**  ***Instruction to interviewers: several days means 1-7; more than half the days means 7-11 days; nearly every day means 12-14 days*** | | | | | | | | | | |
|  | | Not at all | | Several days | | More than half the days | | Nearly every day | No answer | |
|  | Little interest or pleasure in doing things?  **Umehisi umekosa hamu au furaha ya kufanya vitu?** | 0 | | 1 | | 2 | | 3 | 99 | |
|  | Feeling down, depressed or hopeless?  **Umejisikia kuwa mdhaifu, kuhuzunika au bila matumaini?** | 0 | | 1 | | 2 | | 3 | 99 | |
|  | Trouble falling or staying asleep, or sleeping too much?  **Umekuwa na shida kupata usingizi au kutolala au kulala sana?** | 0 | | 1 | | 2 | | 3 | 99 | |
|  | Feeling tired or having little energy?  **Umehisi kuchoka ama kuwa na nguvu kidogo?** | 0 | | 1 | | 2 | | 3 | 99 | |
|  | Poor appetite or overeating?  **Umekosa hamu ya chakula au ukakula zaidi?** | 0 | | 1 | | 2 | | 3 | 99 | |
|  | Feeling bad about yourself – or that you are a failure or have let yourself or your family down?  **Umesikia vibaya juu yako mwenyewe-au kwamba umekosa kufaulu au umejidhalilisha au jamii yako**? | 0 | | 1 | | 2 | | 3 | 99 | |
|  | Trouble concentrating on things, such as reading the newspaper or watching television?  **Umekuwa na shida ya kumakinika kwa vitu kama vile kusoma gazeti au kuangalia runinga?** | 0 | | 1 | | 2 | | 3 | 99 | |
|  | Moving or speaking so slowly that other people could have noticed? Or the opposite – being so fidgety or restless that you have been moving around a lot more than usual?  **Umejipata ukitembea au kuongea polepole sana kiasi cha kwamba watu wangegundua? Au kinyume-kuwa na wasiwasi au kuhangaika hata ya kwamba umekuwa ukizungukazunguka zaidi ya kawaida?** | 0 | | 1 | | 2 | | 3 | 99 | |
|  | Thoughts that you would be better off dead or of hurting yourself in some way?  **Umefikiria ingekuwa bora zaidi ungekufa au kujiumiza katika njia fulani?** | 0 | | 1 | | 2 | | 3 | 99 | |
| COLUMN TOTALS:________+__________+_________= | | | | | | | | | | |
|  | Feeling nervous, anxious or on edge?  **Kuhisi wasiwasi, kukosa utulivu ama kuhisi kama umekasirika Zaidi?** | 0 | | 1 | | 2 | | 3 | 99 | |
|  | Not being able to stop or control worrying  **Umeshindwa kuacha kuhisi wasiwasi ama kudhibiti kuhisi wasiwasi.** | 0 | | 1 | | 2 | | 3 | 99 | |
|  | Worrying too much about different things  **Kuwa na wasi wasi kuhusu vitu vingi tofauti** | 0 | | 1 | | 2 | | 3 | 99 | |
|  | Trouble relaxing  **Shida kutulia** | 0 | | 1 | | 2 | | 3 | 99 | |
|  | Being so restless that it is hard to sit still  **Kukosa utulivu** | 0 | | 1 | | 2 | | 3 | 99 | |
|  | Becoming easily annoyed or irritable  **Kuwa na hasira kwa urahisi** | 0 | | 1 | | 2 | | 3 | 99 | |
|  | Feeling afraid as if something awful might happen  **Kuhisi hofu kama kitu kibaya kitatendeka au kutokea** | 0 | | 1 | | 2 | | 3 | 99 | |
| **Sometimes when people feel overwhelmed by life or by a situation they can intentionally cause harm to themselves.**  **Wakati mwingine wakati watu wanahisi kusumbuliwa na maisha au kwa hali waliyomo mpaka wanaweza kujiumiza wenyewe kimakusudi.** | | | | | | | | | | |
|  | Have you **ever** intentionally or on purpose, cut yourself or done anything else to harm yourself (without intending to kill yourself)?  **Umewahi kujikata au kujifanyia kitu chochote kingine ili kujiumiza kimakusudi (bila kutaka kujiua)?** | Yes………………………………..1  No…………………………………2 **SKIP TO Q1219**  No answer……………………….99 **SKIP TO Q1219** | | | | | | | | |
|  | Has this happened in the past 30 days?  **Je, hili limetokea siku 30 zilizopita?** | Yes……………………………….1  No………………………………..2  No answer………………………99 | | | | | | | | |
|  | Have you **ever** thought about ending your life?  **Umewahi fikiria kujitoa uhai?** | Yes………………………………..1  No…………………………………2 **SKIP TO Q1221**  No answer………………… 99 **SKIP TO Q1221** | | | | | | | | |
|  | Has this happened in the past 30 days?  **Umefikiria kujitoa uhai siku 30 zilizopita?** | Yes……………………………….1  No………………………………..2  No answer………………………99 | | | | | | | | |
|  | Have you **ever** attempted to end your life (kill yourself)?  **Umewahi jaribu kujitoa uhai?** | Yes………………………………..1  No…………………………………2 **SKIP TO Q1223**  No answer………………… 99 **SKIP TO Q1223** | | | | | | | | |
|  | Has this happened in the past 30 days?  **Umejaribu kujtoa uhai kwa siku 30 zilizopita?** | Yes……………………………….1  No………………………………..2  No answer………………………99 | | | | | | | | |
| **POST TRAUMATIC STRESS DISORDER (HTS-17 Tool)** | | | | | | | | | | |
| Below is a list of problems and complaints that women sometimes have in response to stressful life experiences.  **In the past one month, how much have you been bothered by….**  **Ifuatayo ni orodha ya matatizo na malalamiko ambayo wanawake wakati mwingine hupitia kutokana na uzoefu wa maisha magumu. Tafadhali sikiliza kila mmoja kwa uangalifu na sema ni kiasi gani umesumbuliwa na tatizo hili katika mwezi mmoja uliopita.** | | | | | | | | | | |
|  | | | Not at all | | A little | Quite a lot | Extremely often | | | No answer |
|  | Repeated, disturbing *memories, thoughts, or images* of a stressful experience from the past?  **Kumbukumbu zinazojirudia, kumbukumbu za kusumbua, mawazo, au picha za uzoefu wa kusumbua kutoka zamani?** | | 0 | | 1 | 2 | 3 | | | 99 |
|  | Repeated, disturbing *dreams* of a stressful  experience from the past?  **Ndoto za kutisha zinazojirudia kuhusu jambo mbaya lililotendeka zamani?** | | 0 | | 1 | 2 | 3 | | | 99 |
|  | Suddenly *acting* or *feeling* as if a stressful experience *were happening* again (as if you were reliving it)?  **Kushtuka au kujisikia kama jambo lililokuletea wasiwasi linatendeka tena (kama unalipitia tena)** | | 0 | | 1 | 2 | 3 | | | 99 |
|  | | | Not at all | | A little | Quite a lot | Extremely often | | | No answer |
|  | Feeling *very upset* when *something reminded* you of a stressful experience from the past?  **Kuhisi wasiwasi na kukasirika sana wakati kitu kinakukumbusha jambo mbaya lililotokea zamani?** | | 0 | | 1 | 2 | 3 | | | 99 |
|  | Having *physical reactions* (e.g., heart pounding, trouble breathing, or sweating) when *something reminded* you of a stressful experience from the past?  **Kuwa na athari za kimwili (k.m., moyo kupiga zaidi, shida kupumua, au kutoa jasho) wakati kitu kinakumkumbusha jambo mbaya lililotendeka zamani?** | | 0 | | 1 | 2 | 3 | | | 99 |
|  | Avoid *thinking about* or *talking about* a stressful experience from the past or avoid *having feelings* related to it?  **Kuepuka kufikiria au kuzungumza juu ya mambo yaliyotendeka zamani au kuepuka kuwa na hisia zinazohusiana nayo?** | | 0 | | 1 | 2 | 3 | | | 99 |
|  | Avoid *activities* or *situations* because they *remind you* of a stressful experience from the past?  **Kuepuka shughuli au hali kwa sababu zinakukumbusha uzoefu mambo yasiyopendeza yaliyotendeka zamani?** | | 0 | | 1 | 2 | 3 | | | 99 |
|  | Trouble *remembering important parts* of a stressful experience from the past?  **Shida kukumbuka sehemu muhimu za mambo mabaya yaliyotendeka zamani?** | | 0 | | 1 | 2 | 3 | | | 99 |
|  | Loss of *interest in things that you used to enjoy?*  **Kupoteza hamu katika mambo ambayo ulikuwa unafurahia?** | | 0 | | 1 | 2 | 3 | | | 99 |
|  | *Difficulty in falling or staying asleep?*  **Ugumu wa kupata usingizi ama kutolala vizuri** | | 0 | | 1 | 2 | 3 | | | 99 |
|  | Feeling *distant* or *cut* off from other people?  **Kuhisi kama umejitenga sana na watu wengine?** | | 0 | | 1 | 2 | 3 | | | 99 |
|  | Feeling *emotionally numb* or being unable to have loving feelings for those close to you?  **Kuhisi kama mwenye hana hisia za upendo kwa walio karibu nawe?** | | 0 | | 1 | 2 | 3 | | | 99 |
|  | Feeling as if your *future* will somehow be *cut short*?  **Kuhisi kama maisha yako ya usoni yatakatizwa/kumalizwa kwa namna fulani?** | | 0 | | 1 | 2 | 3 | | | 99 |
|  | Feeling *irritable* or having *angry outbursts*?  **Kuhisi kuwa na hasira au kuwa na ghadhabu?** | | 0 | | 1 | 2 | 3 | | | 99 |
|  | Having *difficulty concentrating*?  **Kuwa na ugumu kumakinika?** | | 0 | | 1 | 2 | 3 | | | 99 |
|  | Being *“super alert”* or watchful on guard?  **Kuwa macho au kutahadhari kila wakati?** | | 0 | | 1 | 2 | 3 | | | 99 |
|  | Feeling *jumpy* or easily startled?  **Kuhisi kushtuliwa kwa urahisi?** | | 0 | | 1 | 2 | 3 | | | 99 |

| SECTION 13. ALCOHOL AND DRUG USE (WHO ASSIST TOOL) | | | | | | | |
| --- | --- | --- | --- | --- | --- | --- | --- |
| Some people consume alcohol and drugs for no-medical reasons to feel good, get high, fly, trip or fantasties. These next questions are about your use of tobacco, alcohol and drugs. We will keep your answers completely confidential.Watu wengine hutumia madawa kwa sababu zisizo za kimatibabu kujisikia vizuri, kupata high, kuruka, safari au fantasies. Maswali haya yafuatayo ni kuhusu matumizi yako ya tumbaku, pombe na madawa ya kulevya. Tutaweka majibu yako kwa siri kabisa. | | | | | | | |
| **Q1301** | **Tobacco products (cigarettes, chewing tobacco, cigars, sheesha etc.)?** | | | | | | |
|  | In your life have you ever used Tobacco products?  **Maishani mwako, ushawaitumia bidhaa zozote za tumbaku?** | No……………………………0 **Skip to Q1302**  Yes…………………………..1  No answer………………….99 | | | | | |
|  |  | Never | Once or twice | Monthly | Weekly | Daily or almost daily | No Answer |
| a. | In the past 3 months, how often have you used tobacco?  **Kwa muda wa miezi mitatu iliyopita, umetumia tumbaku mara ngapi?** | 0 | 2 | 3 | 4 | 6 | 99 |
| b. | During the past 3 months, how often have you had a strong desire to use tobacco?  **Kwa muda wa miezi mitatu iliyopita, umekuwa na hamu kali ya kutumia tumbaku mara ngapi?** | 0 | 3 | 4 | 5 | 6 | 99 |

| **Q1302** | **Alcoholic beverages (beer, wine, spirits, chang’aa busaa etc.)** | | | | | | |
| --- | --- | --- | --- | --- | --- | --- | --- |
|  | In your life have you ever used Alcohol?  **Maishani mwako, ushaitumia pombe?** | No……………………………0 **Skip to Q1303**  Yes…………………………..1  No answer………………….99 | | | | | |
|  |  | Never | Once or twice | Monthly | Weekly | Daily or almost daily | No Answer |
| a | In the past 3 months, how often have you used alcohol?  **Kwa miezi mitatu iliyopita, umetumia pombe mara ngapi?** | 0  **skip to e** | 2 | 3 | 4 | 6 | 99 |
| b | During the past 3 months, how often have you had a strong desire to use alcohol?  **Kwa miezi mitatu iliyopita, umehisi hamu kali ya kutumia pombe mara ngapi?** | 0 | 3 | 4 | 5 | 6 | 99 |
| c | During the past 3 months how often has your use of alcohol led to health, social, legal or financial problems, such as bad hangovers, vomiting, stomach pain, sleeping poorly, violence, doing things while you are drunk that you later regret?  **Katika miezi 3 iliyopita, ni mara ngapi matumizi yako ya pombe yamesababisha matatizo ya afya kama hangie, kutapika, kuumwa na tumbo shida ya kupata usingizi, vurugu na kufanya mambo unayojuta baadaye, kijamii, kisheria au kifedha?** | 0 | 4 | 5 | 6 | 7 | 99 |
| d | During the past 3 months how often have you failed to do what was normally expected of you because of your use of alcohol, such as missing work or neglecting to look after your child or house properly?  **Katika kipindi cha miezi mitatu iliyopita, ni mara ngapi umeshindwa kufanya kile ambacho ulitarajia kwa sababu ya matumizi yako ya pombe?** | 0 | 5 | 6 | 7 | 8 | 99 |
|  |  | Never | Yes, but not in the past 3 months | | Yes, in the past 3 months | | No Answer |
| e | Have you ever tried to cut down or stop using alcohol but failed?  **Je, umewahi kujaribu na kushindwa kudhibiti, kupunguza au kuacha kutumia pombe?** | 0 | 3 | | 6 | | 99 |
| f | Has a friend or relative or anyone else *ever* expressed concern about your use of alcohol?  **Rafiki au jamaa au mtu yeyote amewahi kuelezea kuwa ana wasiwasi juu ya matumizi yako ya pombe?** | 0 | 3 | | 6 | | 99 |
|  | **Alcohol Total (a-f):____________+____________=**___ | | | | | | |
| **Q1303** | **Cannabis (marijuana, pot, grass, hash, bhang, weed etc.)** | | | | | | |
|  | In your life have you ever used Cannabis?  **Maishani mwako, ushawaitumia bangi?** | No……………………………0 **Skip to Q1304**  Yes…………………………..1  No answer………………….99 | | | | | |

|  |  | Never | Once or twice | Monthly | Weekly | Daily or almost daily | No Answer |
| --- | --- | --- | --- | --- | --- | --- | --- |
| a | In the past 3 months, how often have you used cannabis?  **Kwa miezi mitatu iliyopita, umetumia bangi mara ngapi?** | 0  **skip to e** | 2 | 3 | 4 | 6 | 99 |
| b | During the past 3 months, how often have you had a strong desire to use cannabis?  **Kwa miezi mitatu iliyopita, umehisi hamu ya kutumia bangi mara ngapi?** | 0 | 3 | 4 | 5 | 6 | 99 |
| c | During the past 3 months how often has your use of cannabis led to health, social, legal or financial problems, *such as forgetting to do things, difficulty paying attention or getting motivated, problems getting organised, feeling depressed or anxious… ?”*  **Katika miezi 3 iliyopita, ni mara ngapi matumizi yako ya bangi yamesababisha matatizo ya kijamii, Kisheria, kifedha au ki afya kama vile kusahau kufanya mambo, ugumu kumakinika kwa jambo au kupata motisha, matatizo ya kujipaga, hisia huzuni au wasiwasi? "** | 0 | 4 | 5 | 6 | 7 | 99 |
| d | During the past 3 months how often have you failed to do what was normally expected of you because of your use of cannabis, such as missing work or neglecting to look after your child or house properly?  **Katika kipindi cha miezi mitatu iliyopita, ni mara ngapi umeshindwa kufanya kile ambacho ulitarajiwa kama kuenda kazini,kutochunga mtoto ama nyumba yako vizuri kwa sababu ya matumizi yako ya bangi?** | 0 | 5 | 6 | 7 | 8 | 99 |
|  |  | Never | Yes, but not in the past 3 months | | Yes, in the past 3 months | | No Answer |
| e | Has a friend or relative or anyone else *ever* expressed concern about your use of cannabis?  **Rafiki au jamaa au mtu yeyote amewahi kuelezea kuwa ana wasiwasi juu ya matumizi yako ya bangi?** | 0 | 3 | | 6 | | 99 |
| f | Have you ever tried to cut down or stop using cannabis but failed?  **Je, umewahi kujaribu na kushindwa kudhibiti, kupunguza au kuacha kutumia bangi?** | 0 | 3 | | 6 | | 99 |
| **Q1304** | **Cocaine (coke, crack, unga etc.)** | | | | | | |
|  | In your life have you ever used cocaine?  **Maishani mwako, ushawaitumia cocaine?** | No……………………………0 **Skip to Q1305**  Yes…………………………..1  No answer………………….99 | | | | | |
|  |  | Never | Once or twice | Monthly | Weekly | Daily or almost daily | No Answer |
| a | In the past 3 months, how often have you used Cocaine?  **Kwa miezi mitatu iliyopita, umetumia cocaine mara ngapi?** | 0  **skip to e** | 2 | 3 | 4 | 6 | 99 |
| b | During the past 3 months, how often have you had a strong desire to use Cocaine?  **Kwa miezi mitatu iliyopita, umehisi hamu kali ya kutumia cocaine mara ngapi?** | 0 | 3 | 4 | 5 | 6 | 99 |
|  |  | Never | Once or twice | Monthly | Weekly | Daily or almost daily | No Answer |
| c | During the past 3 months how often has your use of Cocaine led to health, social, legal or financial problems?  **Katika miezi 3 iliyopita, ni mara ngapi matumizi yako ya cocaine imesababisha matatizo ya afya, kijamii, kisheria au kifedha?** | 0 | 4 | 5 | 6 | 7 | 99 |
| d | During the past 3 months how often have you failed to do what was normally expected of you because of your use of Cocaine, , such as missing work or neglecting to look after your child or house properly?  **Katika kipindi cha miezi mitatu iliyopita, ni mara ngapi umeshindwa kufanya kile ambacho ulitarajiwa kama vile kutoenda kazini, kutochunga mtoto wako ama nyumba vizuri kwa sababu ya matumizi yako ya cocaine?** | 0 | 5 | 6 | 7 | 8 | 99 |
|  |  | Never | Yes, but not in the past 3 months | | Yes, in the past 3 months | | No Answer |
| e | Has a friend or relative or anyone else *ever* expressed concern about your use of Cocaine?  **Rafiki au jamaa au mtu yeyote amewahi kuelezea kuwa ana wasiwasi juu ya matumizi yako ya cocaine?** | 0 | 3 | | 6 | | 99 |
| f | Have you ever tried to cut down or stop using Cocaine but failed?  **Je, umewahi kujaribu na kushindwa kudhibiti, kupunguza au kuacha kutumia cocaine?** | 0 | 3 | | 6 | | 99 |
| **Q1305** | **Amphetamine type stimulants (Khat/miraa, speed, diet pills, ecstasy, etc.)** | | | | | | |
|  | In your life have you ever used Amphetamines?  **Maishani mwako, umeshawitumia amphetamines?** | No……………………………0 **Skip to Q1306**  Yes…………………………..1  No answer………………….99 | | | | | |
|  |  | Never | Once or twice | Monthly | Weekly | Daily or almost daily | No Answer |
| a | In the past 3 months, how often have you used Amphetamines?  **Kwa miezi mitatu iliyopita, umetumia amphetamines mara ngapi?** | 0  **skip to e** | 2 | 3 | 4 | 6 | 99 |
| b | During the past 3 months, how often have you had a strong desire to use Amphetamines?  **Kwa miezi mitatu iliyopita umehisi hamu ya kutumia amphetamines mara ngapi?** | 0 | 3 | 4 | 5 | 6 | 99 |
| c | During the past 3 months how often has your use of Amphetamines led to health, social, legal or financial problems, such as having a bad ‘come-down’ and feeling depressed, anxious and irritable the day or so after you have used, feeling angry, aggressive or uptight, getting headaches, sleeping poorly, dental problems from grinding your teeth?  **Katika miezi 3 iliyopita, ni mara ngapi matumizi yako ya amphetamines yamesababisha matatizo ya afya, kijamii,**  **kisheria au kifedha kwa mfano come-down mbaya, kuhisi**  **wasiwasi na kiwewe, kukereka, kuumwa na kichwa, matatizo ya usingizi na shida ya meno inayotokana na kuyasaga?** | 0 | 4 | 5 | 6 | 7 | 99 |

|  |  | | Never | Once or twice | Monthly | Weekly | Daily or almost daily | No Answer |
| --- | --- | --- | --- | --- | --- | --- | --- | --- |
| D | During the past 3 months how often have you failed to do what was normally expected of you because of your use of Amphetamines, , such as missing work or neglecting to look after your child or house properly?  **Katika kipindi cha miezi mitatu iliyopita, ni mara ngapi umeshindwa kufanya kile ambacho ulitarajiwa kwa mfano kutoenda kazini ama kutochunga mtoto ama nyumba yako vizuri kwa sababu ya matumizi yako ya amphetamines?** | | 0 | 5 | 6 | 7 | 8 | 99 |
|  |  | | Never | Yes, but not in the past 3 months | | Yes, in the past 3 months | | No Answer |
| e | Has a friend or relative or anyone else *ever* expressed concern about your use of Amphetamines?  **Rafiki au jamaa au mtu yeyote amewahi kuelezea kuwa ana wasiwasi juu ya matumizi yako ya amphetamines?** | | 0 | 3 | | 6 | | 99 |
| f | Have you ever tried to cut down or stop using Amphetamines but failed?  **Je, umewahi kujaribu na kushindwa kudhibiti, kupunguza au kuacha kutumia amphetamines?** | | 0 | 3 | | 6 | | 99 |
| **Q1306** | **Inhalants (nitrous, glue/ gum, petrol, paint thinner, etc.)** | | | | | | | |
|  | In your life have you ever used Inhalants?  **Maishani mwako, ushawaitumia inhalants?** | | No……………………………0 **Skip to Q1307**  Yes…………………………..1  No answer………………….99 | | | | | |
|  |  | | Never | Once or twice | Monthly | Weekly | Daily or almost daily | No Answer |
| a | In the past 3 months, how often have you used Inhalants?  **Kwa miezi mitatu iliyopita, umetumia inhalants mara ngapi?** | | 0  **skip to e** | 2 | 3 | 4 | 6 | 99 |
| b | During the past 3 months, how often have you had a strong desire to use Inhalants?  **Kwa miezi mitatu iliyopita, umehisi hamu kali ya kutumia inhalants mara ngapi?** | | 0 | 3 | 4 | 5 | 6 | 99 |
| c | During the past 3 months how often has your use of Inhalants led to health, social, legal or financial problems?  **Katika miezi 3 iliyopita, ni mara ngapi matumizi yako ya inhalants imesababisha matatizo ya afya, kijamii, kisheria au kifedha?** | | 0 | 4 | 5 | 6 | 7 | 99 |
| d | During the past 3 months how often have you failed to do what was normally expected of you because of your use of Inhalants, , such as missing work or neglecting to look after your child or house properly?  **Katika kipindi cha miezi mitatu iliyopita, ni mara ngapi umeshindwa kufanya kile ambacho ulitarajiwa kama kutoenda kazini, kutochunga mtoto wako ama nyumba vizuri kwa sababu ya matumizi yako ya inhalants?** | | 0 | 5 | 6 | 7 | 8 | 99 |
|  |  | | Never | Yes, but not in the past 3 months | | Yes, in the past 3 months | | No Answer |
| e | Has a friend or relative or anyone else *ever* expressed concern about your use of Inhalants?]  **Rafiki au jamaa au mtu yeyote amewahi kuelezea kuwa ana wasiwasi juu ya matumizi yako ya inhalants?** | | 0 | 3 | | 6 | | 99 |
|  |  | | Never | Yes, but not in the past 3 months | | Yes, in the past 3 months | | No Answer |
| f | Have you ever tried to cut down or stop using Inhalants but failed?  **Je, umewahi kujaribu na kushindwa kudhibiti, kupunguza au kuacha kutumia inhalants?** | | 0 | 3 | | 6 | | 99 |
| **Q1307** | **Sedatives or Sleeping Pills (Valium, Serepax, Rohypnol, cough syrup, mchele, taptap etc.)** | | | | | | | |
|  | In your life have you ever used Sedatives or Sleeping Pills?  **Maishani mwako, ushawaitumia madawa ya kulala ?** | | No……………………………0 **Skip to Q1308**  Yes…………………………..1  No answer………………….99 | | | | | |
|  |  | | Never | Once or twice | Monthly | Weekly | Daily or almost daily | No Answer |
| a | In the past 3 months, how often have you used Sedatives or Sleeping Pills?  **Kwa miezi mitatu iliyopita, umetumia madawa ya kulala mara ngapi?** | | 0  **skip to e** | 2 | 3 | 4 | 6 | 99 |
| b | During the past 3 months, how often have you had a strong desire to use Sedatives or Sleeping Pills?  **Kwa miezi mitatu iliyopita, umehisi hamu kali ya kutumia madawa ya kulala mara ngapi?** | | 0 | 3 | 4 | 5 | 6 | 99 |
| c | During the past 3 months how often has your use of Sedatives or Sleeping Pills led to health, social, legal or financial problems?  **Katika miezi 3 iliyopita, ni mara ngapi matumizi yako ya madawa ya kulala imesababisha matatizo ya afya, kijamii, kisheria au kifedha?** | | 0 | 4 | 5 | 6 | 7 | 99 |
| d | During the past 3 months how often have you failed to do what was normally expected of you because of your use of Sedatives or Sleeping Pills, such as missing work or neglecting to look after your child or house properly?  **Katika kipindi cha miezi mitatu iliyopita, ni mara ngapi umeshindwa kufanya kile ambacho ulitarajiwa kama kutoenda kazini, kutochunga mtoto ama nyumba yako vizuri kwa sababu ya matumizi yako ya madawa ya kulala?** | | 0 | 5 | 6 | 7 | 8 | 99 |
|  |  | | Never | Yes, but not in the past 3 months | | Yes, in the past 3 months | | No Answer |
| e | Has a friend or relative or anyone else *ever* expressed concern about your use of Sedatives or Sleeping Pills?  **Rafiki au jamaa au mtu yeyote amewahi kuelezea kuwa ana wasiwasi juu ya matumizi yako ya madawa ya kulala?** | | 0 | 3 | | 6 | | 99 |
| f | Have you ever tried to cut down or stop using Sedatives or Sleeping Pills but failed?  **Je, umewahi kujaribu na kushindwa kudhibiti, kupunguza au kuacha kutumia madawa ya kulala?** | | 0 | 3 | | 6 | | 99 |
| **Q1308** | **Hallucinogens (LSD, acid, magic mushrooms, PCP, Special K, Tropical etc.)** | | | | | | | |
|  | In your life have you ever used Hallucinogens?  **Maishani mwako, umeshawaitumia hallucinogens?** | | No……………………………0 **Skip to Q1309**  Yes…………………………..1  No answer………………….99 | | | | | |
|  |  | | Never | Once or twice | Monthly | Weekly | Daily or almost daily | No Answer |
| a | In the past 3 months, how often have you used Hallucinogens?  **Kwa miezi mitatu iliyopita, umetumia hallucinogens mara ngapi?** | | 0  **skip to e** | 2 | 3 | 4 | 6 | 99 |
|  |  | | Never | Once or twice | Monthly | Weekly | Daily or almost daily | No Answer |
| b | During the past 3 months, how often have you had a strong desire to use Hallucinogens?  **Kwa miezi mitatu iliyopita, umehisi hamu kali ya kutumia hallucinogens mara ngapi?** | | 0 | 3 | 4 | 5 | 6 | 99 |
| c | During the past 3 months how often has your use of Hallucinogens led to health, social, legal or financial problems?  **Katika miezi 3 iliyopita, ni mara ngapi matumizi yako ya hallucinogens imesababisha matatizo ya afya, kijamii, kisheria au kifedha?** | | 0 | 4 | 5 | 6 | 7 | 99 |
| d | During the past 3 months how often have you failed to do what was normally expected of you because of your use of Hallucinogens, such as missing work or neglecting to look after your child or house properly?  **Kwa miezi mitatu iliyopita, ni mara ngapi umeshindwa kufanya kile ambacho ulitarajiwa kwa mfano kutoenda kazini, kutochunga nyumba ama mtoto wako vizuri kwa sababu ya matumizi yako ya hallucinogens?** | | 0 | 5 | 6 | 7 | 8 | 99 |
|  |  | | Never | Yes, but not in the past 3 months | | Yes, in the past 3 months | | No Answer |
| e | Has a friend or relative or anyone else *ever* expressed concern about your use of Hallucinogens?  **Rafiki au jamaa au mtu yeyote amewahi kuelezea kuwa ana wasiwasi juu ya matumizi yako ya hallucinogens?** | | 0 | 3 | | 6 | | 99 |
| f | Have you ever tried to cut down or stop using Hallucinogens but failed?  **Je, umewahi kujaribu na kushindwa kudhibiti, kupunguza au kuacha kutumia hallucinogens?** | | 0 | 3 | | 6 | | 99 |
| **Q1309** | **Opioids (heroin, morphine, methadone, brown sugar , etc.)** | | | | | | | |
|  | In your life have you ever used Opiods?  **Maishani mwako, ushawaitumia opioids?** | | No……………………………0 **Skip to Q1310**  Yes…………………………..1  No answer………………….99 | | | | | |
|  |  | | Never | Once or twice | Monthly | Weekly | Daily or almost daily | No Answer |
| a | In the past 3 months, how often have you used Opiods?  **Kwa miezi mitatu iliyopita, umetumia opioids mara ngapi?** | | 0  **skip to e** | 2 | 3 | 4 | 6 | 99 |
| b | During the past 3 months, how often have you had a strong desire to use Opiods?  **Kwa miezi mitatu iliyopita, umehisi hamu kali ya kutumia opioids mara ngapi?** | | 0 | 3 | 4 | 5 | 6 | 99 |
| c | During the past 3 months how often has your use of Opiods led to health, social, legal or financial problems?  **Katika miezi 3 iliyopita, ni mara ngapi matumizi yako ya opioids yamesababisha matatizo ya afya, kijamii, kisheria au kifedha?** | | 0 | 4 | 5 | 6 | 7 | 99 |
| d | During the past 3 months how often have you failed to do what was normally expected of you because of your use of Opiods, such as missing work or neglecting to look after your child or house properly?  **Katika kipindi cha miezi mitatu iliyopita, ni mara ngapi umeshindwa kufanya kile ambacho ulitarajiwa kama kutoenda kazini, kutochunga mtoto ama nyumba yako vizuri kwa sababu ya matumizi yako ya opioids?** | | 0 | 5 | 6 | 7 | 8 | 99 |
|  |  | | Never | Yes, but not in the past 3 months | | Yes, in the past 3 months | | No Answer |
| e | Has a friend or relative or anyone else *ever* expressed concern about your use of Opiods?  **Rafiki au jamaa au mtu yeyote amewahi kuelezea kuwa ana wasiwasi juu ya matumizi yako ya opioids?** | | 0 | 3 | | 6 | | 99 |
| f | Have you ever tried to cut down or stop using Opioids but failed?  **Je, umewahi kujaribu na kushindwa kudhibiti, kupunguza au kuacha kutumia opioids?** | | 0 | 3 | | 6 | | 99 |
| **Q1310** | Have you **ever** injected drugs for non-medical reasons?  **Umewahi kujidunga madawa kwa sababu zisizo za kimatibabu?** | Never……………………………...…….0 **SKIP TO 1312**  Yes, in the past 3 months……………..1  Yes, but not in the past 3 months…….2  No answer………………………………99 | | | | | | |
| **Q1311** | The last time you injected drugs, did you share the needle with any one?  **Mara ya mwisho ulipojidunga dawa za kulevya, ulitumia hiyo sindano na mtu mwingine?** | Yes 1  No 2  Don’t know 98  No answer 99 | | | | | | |
| **Q1312** | The last time you had sex with a client, were you under the influence of alcohol or drugs?  **Wakati wa mwisho ulipofanya ngono na mteja, ulikuwa umekunywa pombe au madawa ya kulevya?** | Yes 1  No 2  No answer 99 | | | | | | |
| **Q1313** | The last time you had sex with a non-paying partner, were you under the influence of alcohol or drugs?  **Wakati wa mwisho ulipofanya ngono na mpenzi asiye kulipa, ulikuwa umekunywa pombe au madawa ya kulevya?** | Yes 1  No 2  No answer 99 | | | | | | |
| **SECTION 14. COMMUNITY SUPPORT** | | | | | | | | |
|  | Do you have someone who you can talk to about your problems?  **Una mtu ambaye unaweza kuzungumza naye kuhusu matatizo yako?** | Yes…………………………………1  Sometimes…………………..….…2  No………………………..…….…..3  No answer………………….…….99 | | | | | | |
|  | Do you have any family who you can ask for emotional support from who are living in Nairobi (parents, grandparents, siblings, cousins etc)  **Una familia yoyote ambayo unaweza kuomba msaada wa kihisia kutoka kwao wanaoishi Nairobi (wazazi, babu, ndugu, binamu, nk)** | Yes…………………………………1  No…………………………………..2  No answer………….…………….99 | | | | | | |
|  | Do you feel you can seek emotional support from a religious place of worship?  **Unajisikia unaweza kutafuta msaada wa kihisia kutoka mahali pa dini i?** | Yes……………………..……………1  No……………………..……………..2  No because don’t have a place of workship…97  No answer………………………...99 | | | | | | |
|  | Are you a member of a female sex workers CBO?  **Je, wewe ni mwanachama wa CBO ya wafanyakazi wa ngono?** | Yes…………………………….……1  No…………………………………..2  No answer………………….…….99 | | | | | | |
|  | During the past 12 months, have you ever participated in any activities/programs organized by the national networks such as KESWA, KENPUD, GALCK, NYARWEK, etc?  **Katika miezi 12 iliyopita, umewahi kushiriki katika shughuli yoyote / mipango iliyoandaliwa na makundi ya walio kwenye hatari ya kupata VVU kama vile KESWA, KENPUD, GALCK, NYARWEK, nk?** | Yes…………………………………1  No…………………………………..2  No answer……………….……….99 | | | | | | |

| **SECTION 15. PARTICIPATION IN THE STUDY** | | |
| --- | --- | --- |
|  | I would like to know how answering the questions in this questionnaire made you feel.  **Ningependa kujua jinsi kujibu maswali katika dodoso hili kumekufanya uhisi?** |  |
|  | Are there any other questions you think we shouldn’t have asked?  **Kuna maswali mengine unafikiri hatukupaswa kuuliza?** |  |
|  | Are there any other questions you think we should have asked?  **Kuna maswali mengine unafikiri tulipaswa kuuliza?** |  |
|  | Is there anything else that you would like to tell us?  **Je, kuna kitu kingine chochote ungependa kutuambia?** |  |

Study Number: _________________ Date:____/___/_____

| **Question** | **Measure** | **Score / Assessment** | **Action Agreed with participant** |
| --- | --- | --- | --- |
| 807a  808a  809a  810a  811a  812a  813a | IPV | **≥ Once**  **Yes** | Zaina SWOP Nothing |
| 814 | IPV rape past 7 days | **Yes** | Chris / Ann Zaina  Nothing Swop  Nothing |
| 838 | Gang rape past 6 months | **≥ Once**  **Yes** | Zaina SWOP Nothing |
| 840 | Non-IPV rape past 7 days | **Yes** | Chris / Ann Zaina  Nothing Swop  Nothing |
| 1201 - 1209 | Depression score | 0-9 Ok  10-14 Mild    15-20 Mod    ≥20 Severe | Psycho-education leaflet  Zaina SWOP Leaflet  only |
| Q1218 | Self harm past 30 days | **Yes** | Zaina |
| Q1220 | Suicide ideation past 30 days | **Yes** | Zaina |
| Q1222 | Suicide attempt past 30 days | **Yes** | Zaina |
| 1302 | Alcohol use | Score **0-10 Low**  **11-26 Mod**  **≥ 27 Severe** | Psycho-education leaflet    Zaina SWOP Leaflet  only |

Date:____/___/_____

|  | **Measure** | **Score / Assessment** |  |
| --- | --- | --- | --- |
|  | Recent IPV | **≥ Once**  **Yes** |  |
|  | IPV rape past 7 days | **Yes** |  |
|  | Gang rape past 6 months | **≥ Once**  **Yes** |  |
|  | Non-IPV rape past 7 days | **Yes** |  |
|  | Depression score | 0-9 Ok  10-14 Mild    15-20 Mod    ≥20 Severe |  |
|  | Self harm past 30 days | **Yes** |  |
|  | Suicide ideation past 30 days | **Yes** |  |
|  | Suicide attempt past 30 days | **Yes** |  |
|  | Alcohol use score  (ASSIST) | Score **0-10 Low**  **11-26 Mod**  **≥ 27 Severe** |  |
